# Supplementary material for: Scoring Osteoarthritis Reliably in Large Joints and the Spine Using Whole-Body CT: OsteoArthritis Computed Tomography-Score (OACT-Score)
Source: J Pers Med. 2020 Dec 22;11(1):5. doi: 10.3390/jpm11010005 (PMC7822205; doi:10.3390/jpm11010005)

OsteoArthritis Computed Tomography-score (OACT-score)

W.P. Gielis
H. Weinans
F.J. Nap
F.W. Roemer
W. Foppen

University Medical Center Utrecht

Sections

- Shoulder
  - Acromioclavicular joints (AC)
  - Glenohumeral joints (GH)
- Spine
  - Intra-vertebral discs
  - Facet joints
- Hip
- Knee
  - Tibio femoral (TF)
  - Patella femoral (PF)
- Ankle

Acromioclavicular joint (AC)

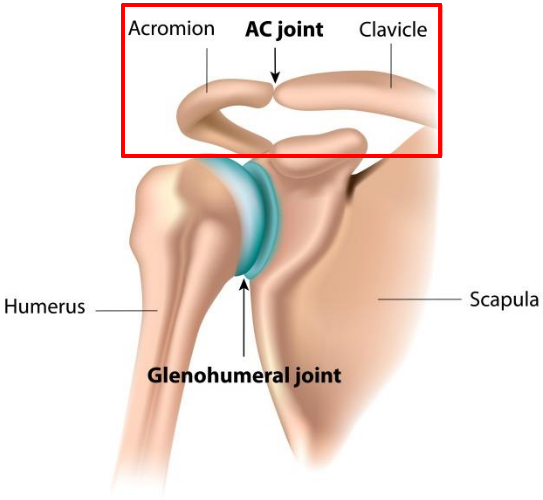

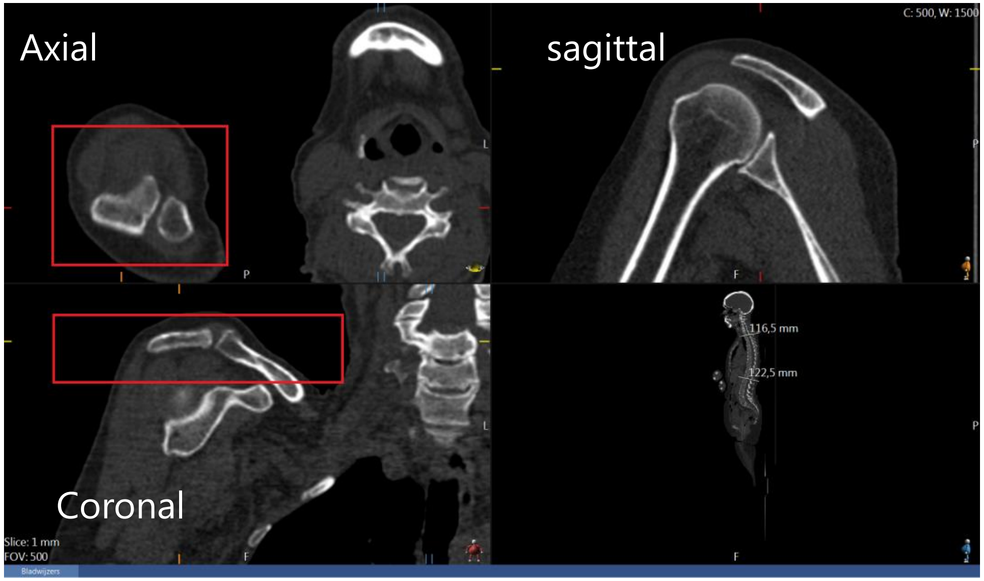

	Acromioclavicular joint (AC)
Predominant view	Coronal and axial
Settings	Multiplanar view, minimal slice thickness, bone window
Sections	Left and Right
Grading	Single grade per section
**AC**	**Definition**
0	No osteophytes or joint space narrowing (JSN)
1	Lipping and/or possible JSN
2	definite osteophytes and/or JSN
3	definite osteophytes and/or JSN and sclerosis and/or cysts and/or bony deformities

Illustration from Shutterstock

Example AC grade 0

AC	Definition
**0**	**No osteophytes or joint space narrowing (JSN)**
1	Lipping and/or possible JSN
2	definite osteophytes and/or JSN
3	definite osteophytes and/or JSN <u>and</u> sclerosis and/or cysts and/or bony deformities

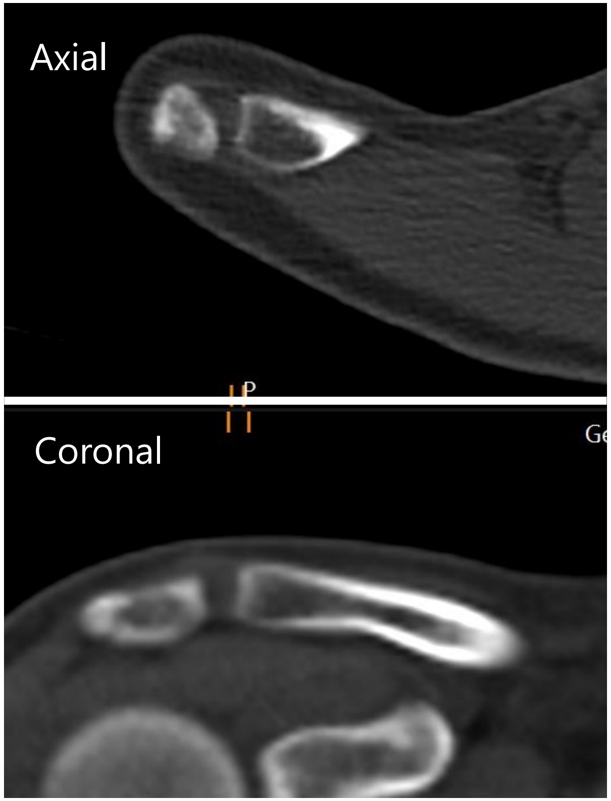

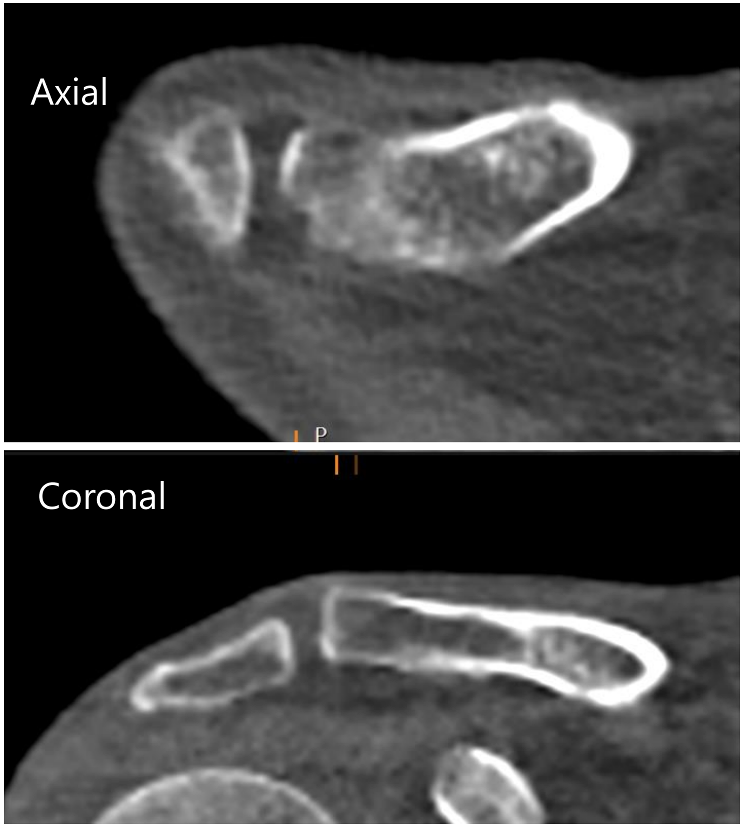

Example AC grade 1

AC	Definition
0	No osteophytes or joint space narrowing (JSN)
**1**	**Lipping and/or possible JSN**
2	definite osteophytes and/or JSN
3	definite osteophytes and/or JSN and<br>sclerosis and/or cysts and/or bony deformities

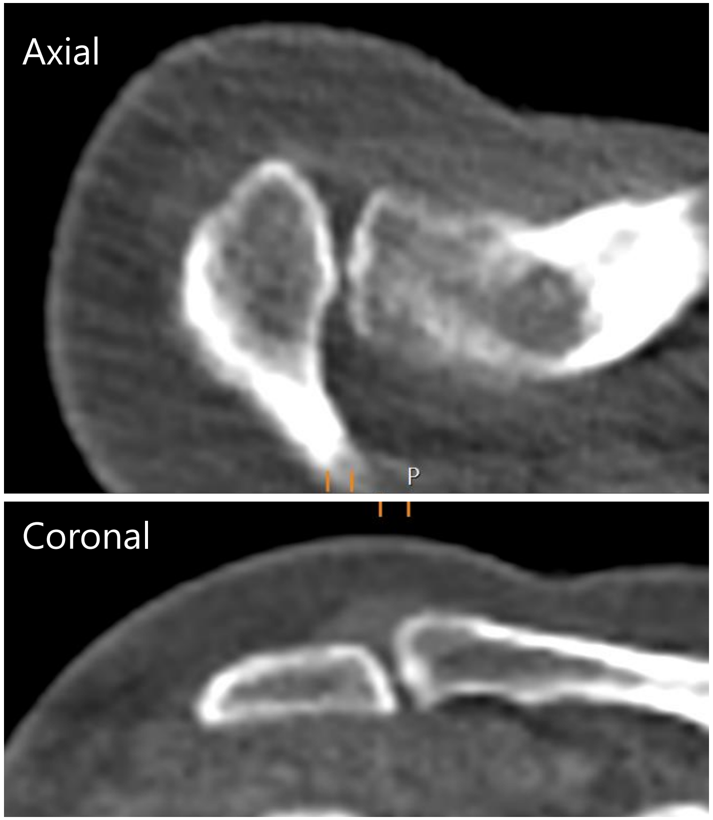

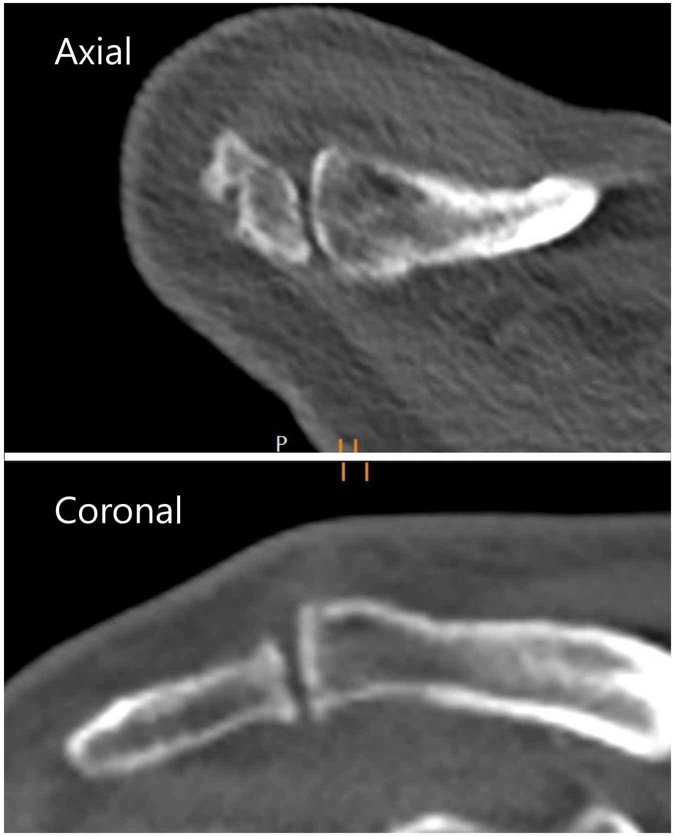

Example AC grade 2

AC	Definition
0	No osteophytes or joint space narrowing (JSN)
1	Lipping and/or possible JSN
**2**	**definite osteophytes and/or JSN**
3	definite osteophytes and/or JSN and sclerosis and/or cysts and/or bony deformities

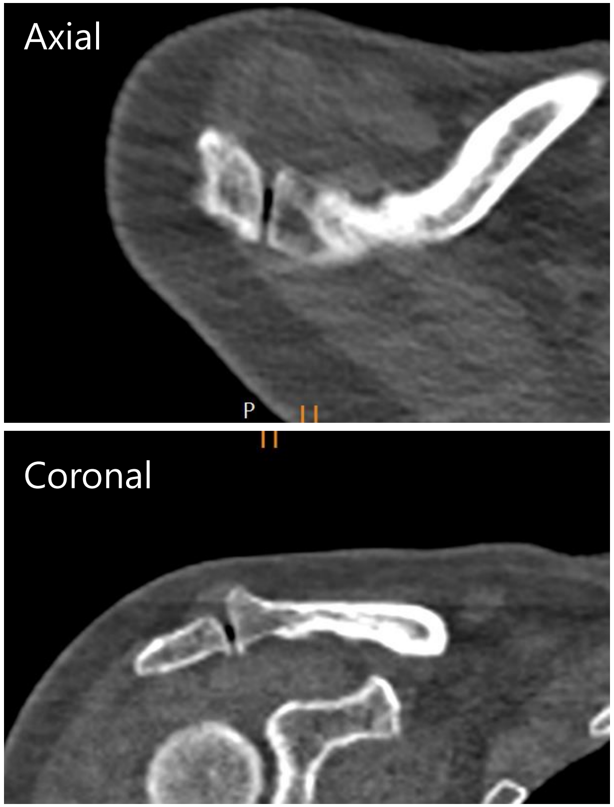

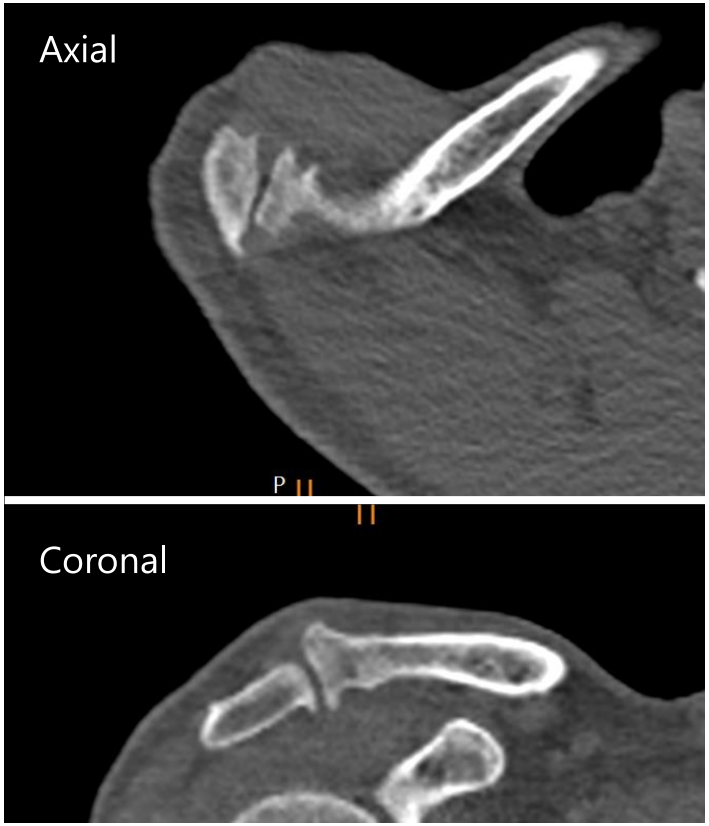

Example AC grade 3

AC	Definition
0	No osteophytes or joint space narrowing (JSN)
1	Lipping and/or possible JSN
2	definite osteophytes and/or JSN
**3**	**definite osteophytes and/or JSN and sclerosis and/or cysts and/or bony deformities**

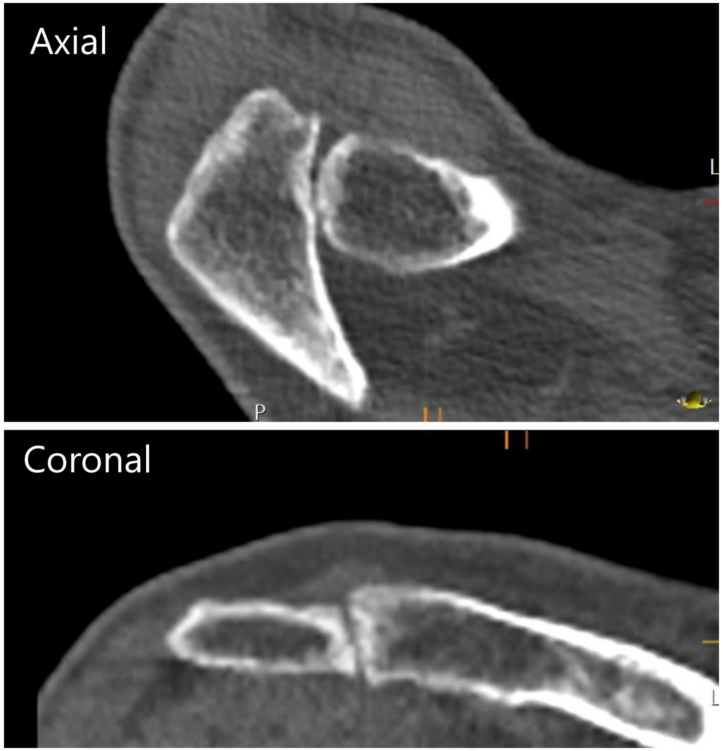

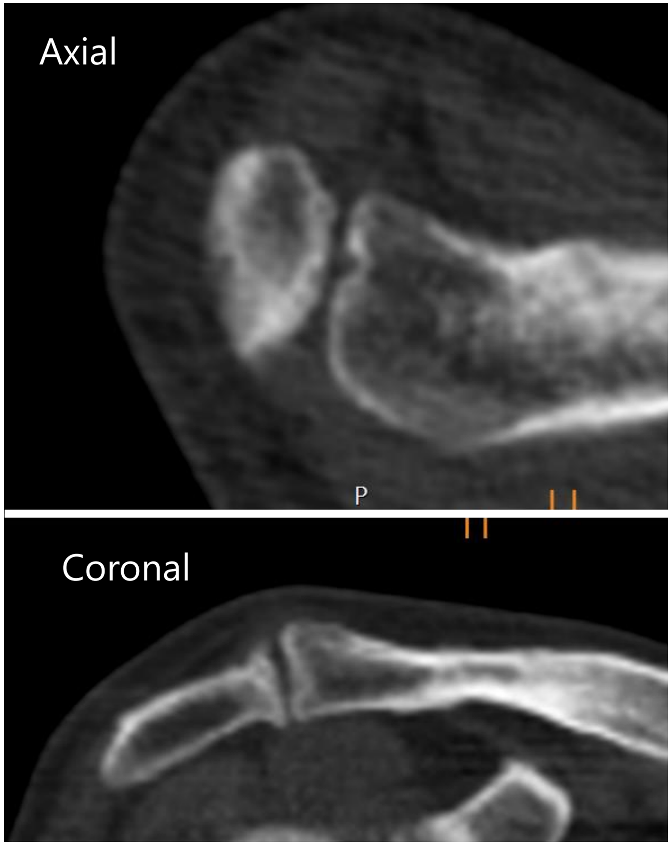

Glenohumeral joint (GH)

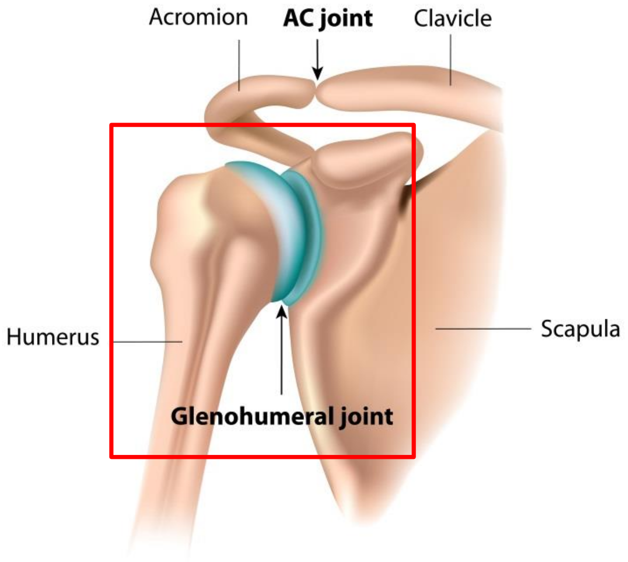

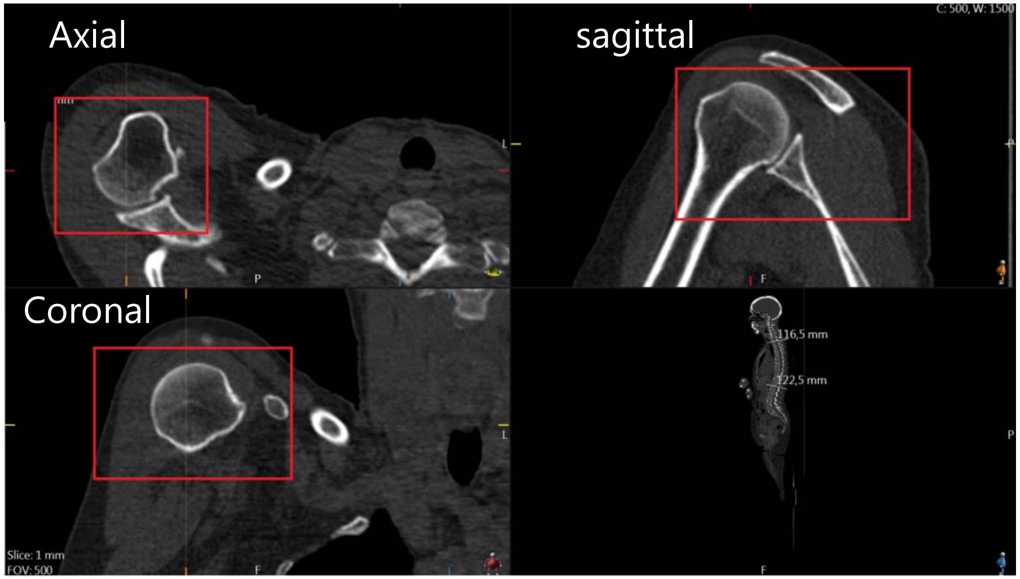

	Glenohumeral joint (GH)
Predominant view	Coronal, axial and sagittal
Settings	Multiplanar view, minimal slice thickness, bone window
Sections	Left and Right
Grading	Single grade per section
**GH**	**Definition**
0	No osteophytes or definite JSN
1	Osteophyte (inferior, anterior, posterior humeral and glenoidal) measured less than 3 mm
2	Osteophyte measured between 3 and 7 mm, slight joint irregularity
3	Osteophyte measured more than 7 mm, definite JSN and/or irregularity.

Illustration from Shutterstock

Example GH Grade 0

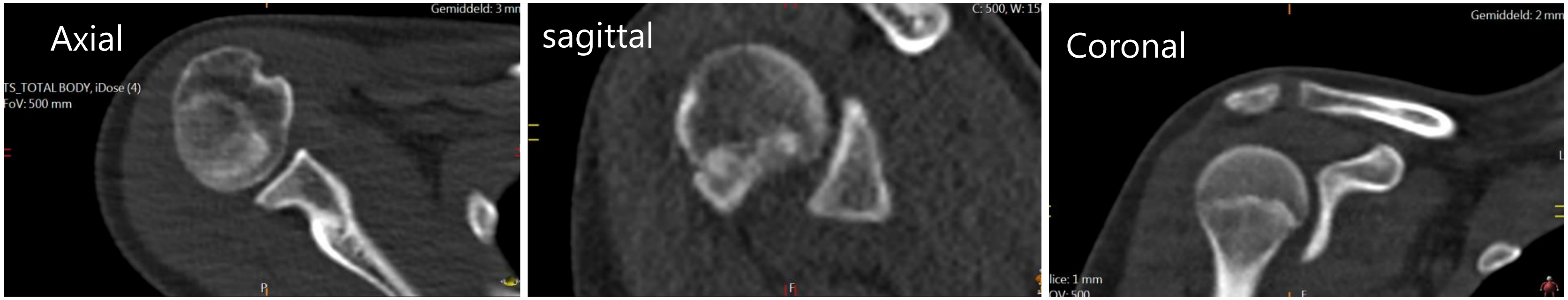

GH	Definition
**0**	**No osteophytes or definite JSN**
1	Osteophyte (inferior, anterior, posterior humeral and glenoidal) measured less than 3 mm
2	Osteophyte measured between 3 and 7 mm, slight joint irregularity
3	Osteophyte measured more than 7 mm, definite JSN and/or irregularity.

Example GH Grade 1

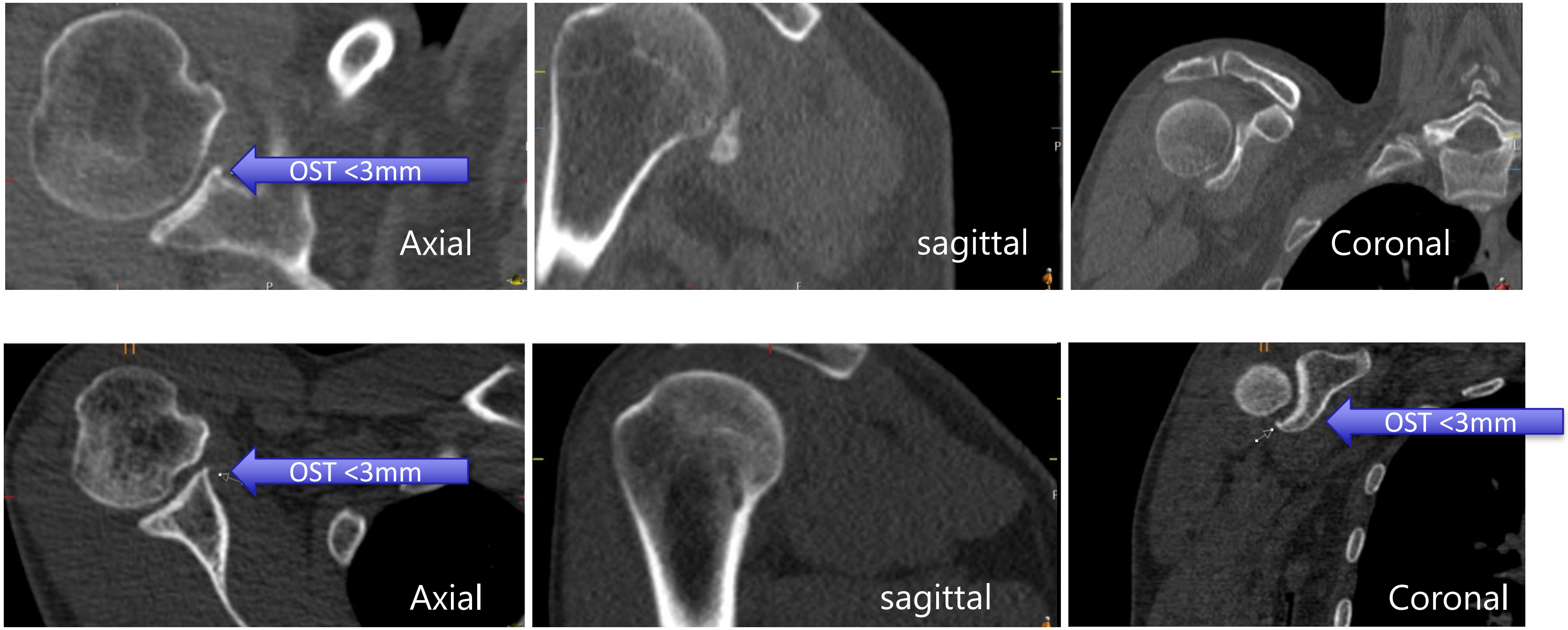

GH	Definition
0	No osteophytes or definite JSN
**1**	**Osteophyte (inferior, anterior, posterior humeral and glenoidal) measured less than 3 mm**
2	Osteophyte measured between 3 and 7 mm, slight joint irregularity
3	Osteophyte measured more than 7 mm, definite JSN and/or irregularity.

Example GH Grade 2

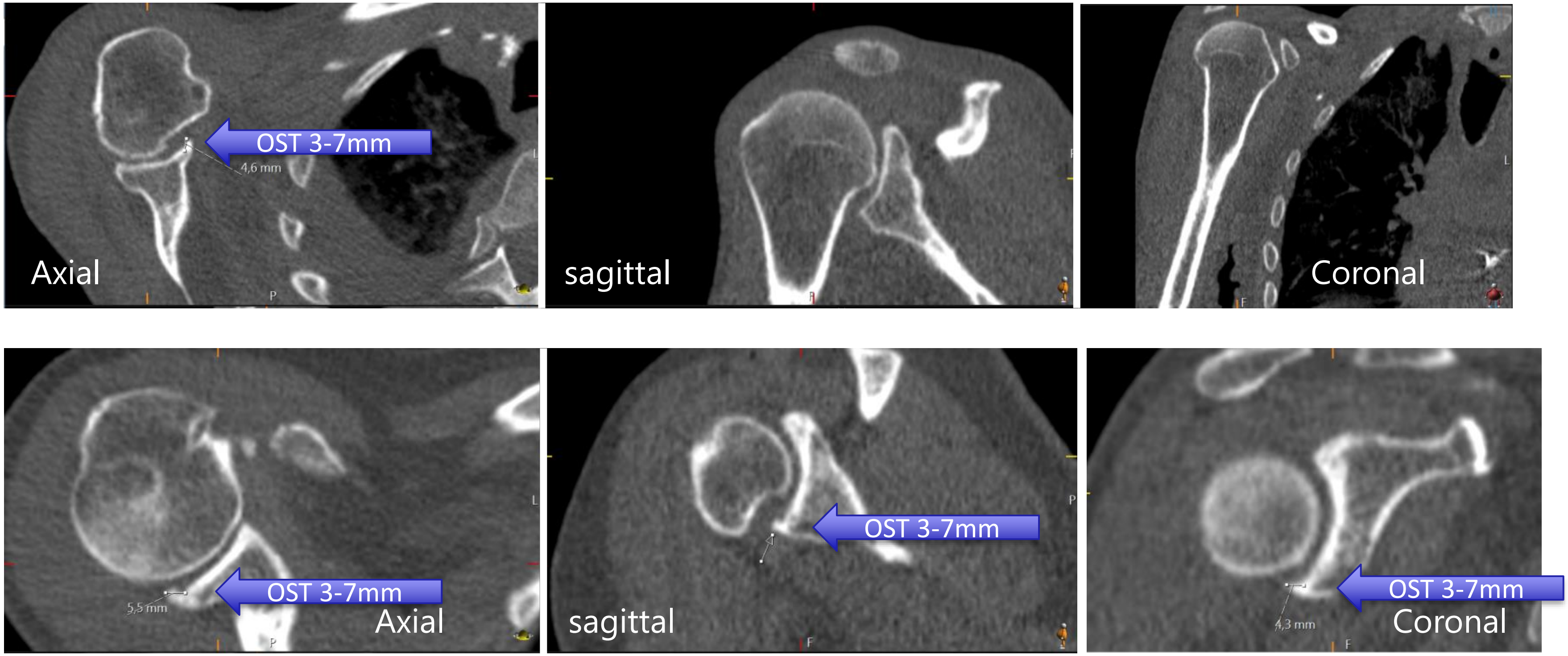

GH	Definition
0	No osteophytes or definite JSN
1	Osteophyte (inferior, anterior, posterior humeral and glenoidal) measured less than 3 mm
**2**	**Osteophyte measured between 3 and 7 mm, slight joint irregularity**
3	Osteophyte measured more than 7 mm, definite JSN and/or irregularity.

Example GH Grade 3

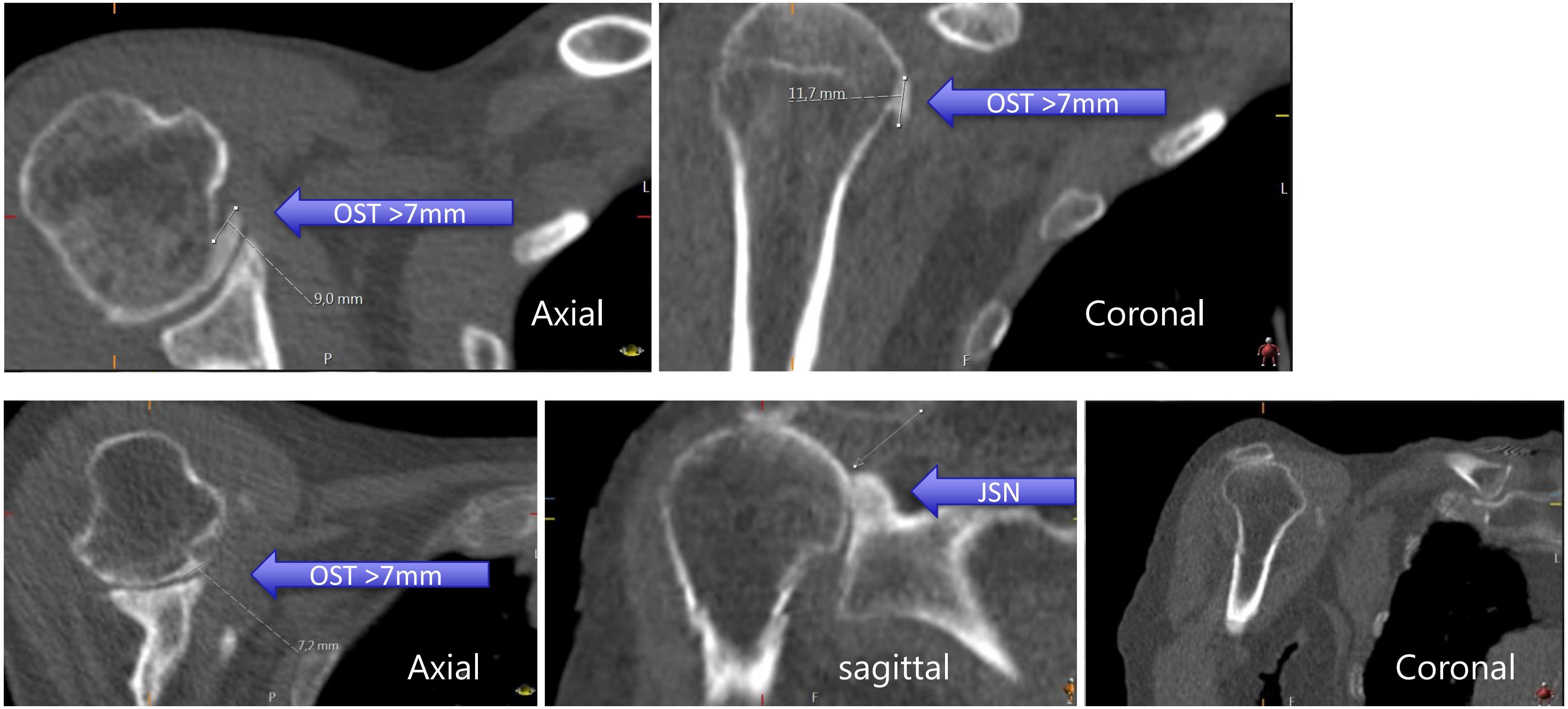

GH	Definition
0	No osteophytes or definite JSN
1	Osteophyte (inferior, anterior, posterior humeral and glenoidal) measured less than 3 mm
2	Osteophyte measured between 3 and 7 mm, slight joint irregularity
**3**	**Osteophyte measured more than 7 mm, definite JSN and/or irregularity.**

Degenerative disc disease (DDD)

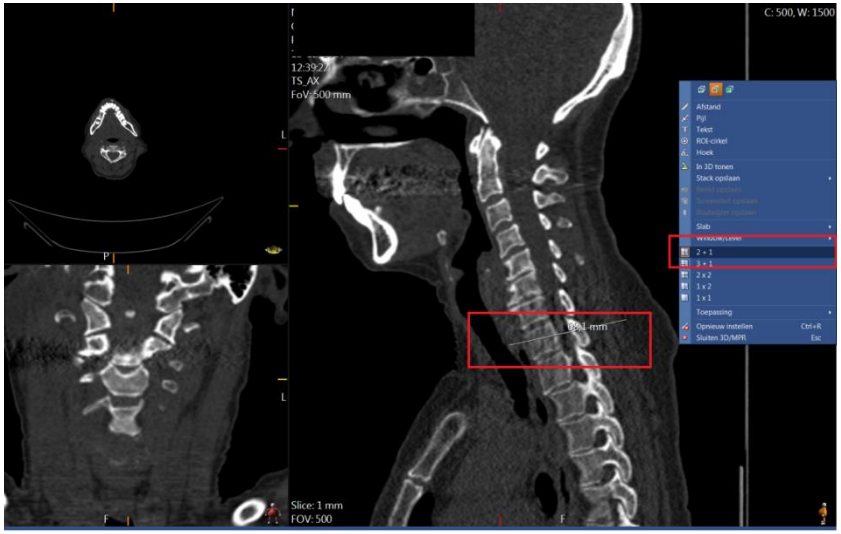

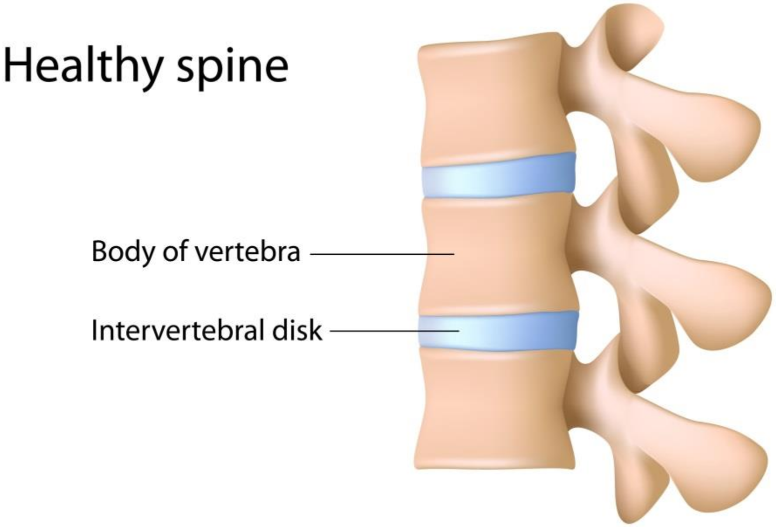

	Degenerative disc disease (DDD)
Predominant view	sagittal
Settings	Multiplanar view, minimal slice thickness, bone window
Sections	Cervical (C2-C3 to C7-T1)<br>Thoracic (T1-T2 to T12-L1)<br>Lumbar (L1-L2 to L5-S1)
Grading	Grade most affected and second most effected level per section for<br>Disc Space Narrowing (DSN)<br>Osteophytes (OST)<br>Sclerosis and endplate Irregularity (SCIR)
**DSN**	**Definition**
0	No Disc Space Narrowing
1	Definite mild narrowing (25-50%)
2	Moderate narrowing (50-75%)
3	Severe loss of disc space (>75%)
**OST**	**Definition**
0	No osteophyte
1	Lipping / small osteophyte
2	Clear moderate osteophyte
3	Clear large osteophyte
**SCIR**	**Definition**
0	No sclerosis or endplate irregularity
1	Visible sclerosis and/or endplate irregularity

Illustration from Shutterstock

Degenerative disc disease (DDD)

- Only the grades of the two most degenerated levels per section (cervical/thoracic/lumbar) will be registered

- To determine the most degenerated levels:
  - Assess DSN of all IVD levels and select two levels with highest DSN score
  - When more then two levels qualify:
    - Assess endplate irregularity and sclerosis of the levels with the highest DSN score
    - Select two levels with highest DSN and SCIR
  - When more then two levels qualify:
    - Assess osteophytosis of the levels with the highest DSN score and highest SCIR
    - Select two levels with highest DSN, SCIR and OST
  - When two levels show exactly the same semi-quantitative scores:
    - Choose most affected level based on qualitative assessment of degeneration from those two levels

All degenerative features are scored, also if they might be "physiological" considering subjects age!

Disc narrowing

- Normal disc height is influenced by position of the vertebral bodies
- Flexion may decrease the disc height on the inside of the bend and increase disc height on the outside of the bend
- See exaggerated example, red lines are of the same lengths

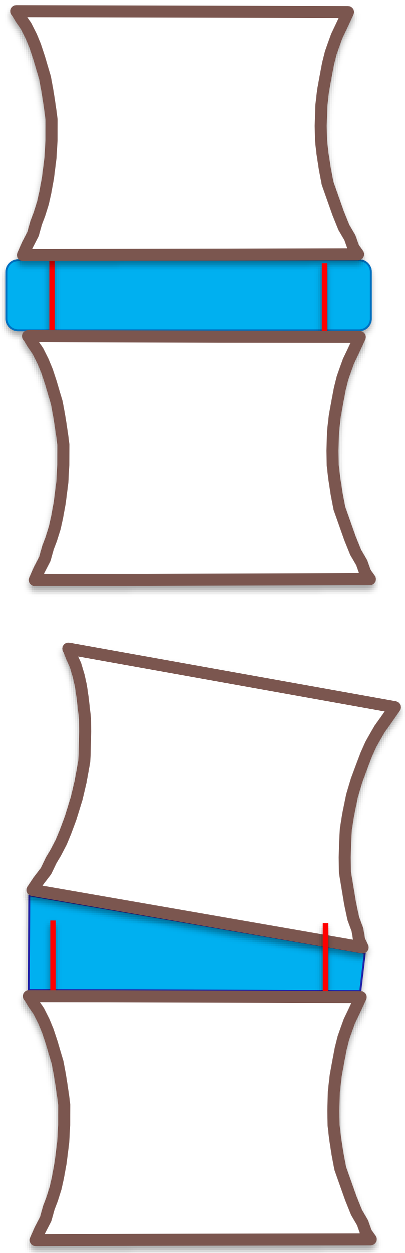

Disc narrowing

- Compare disc height in nine regions to healthy disc height

DSN	Definition	
0	No / possible narrowing	
1	Mild narrowing	25 - 50%
2	Moderate narrowing	≥ 50 - 80%
3	Severe loss of disc space	≥ 80-%

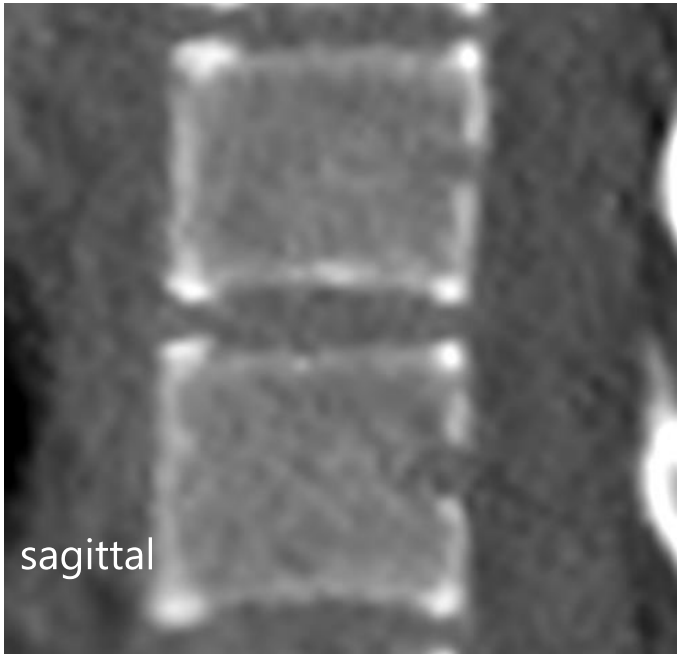

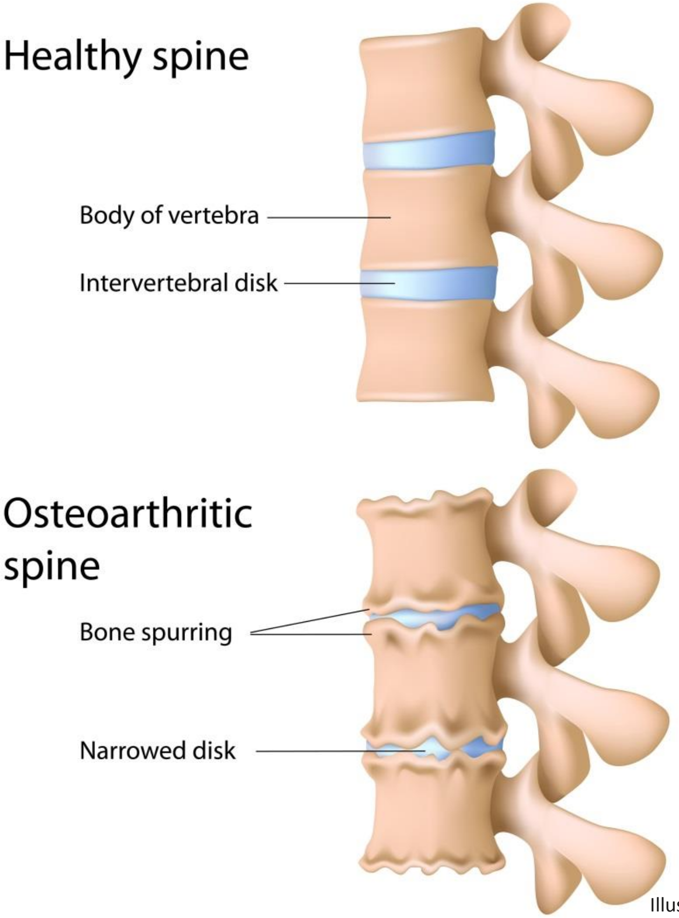

Illustration from Shutterstock

Disc narrowing

- Keep in mind physiological disc height distribution

- Next slides will show normal disc height of a 20 year old, which serves as a visual reference
  - Vertebral levels are numbered in white
  - Examples show 3 sagittal slices of the same patient next to each other

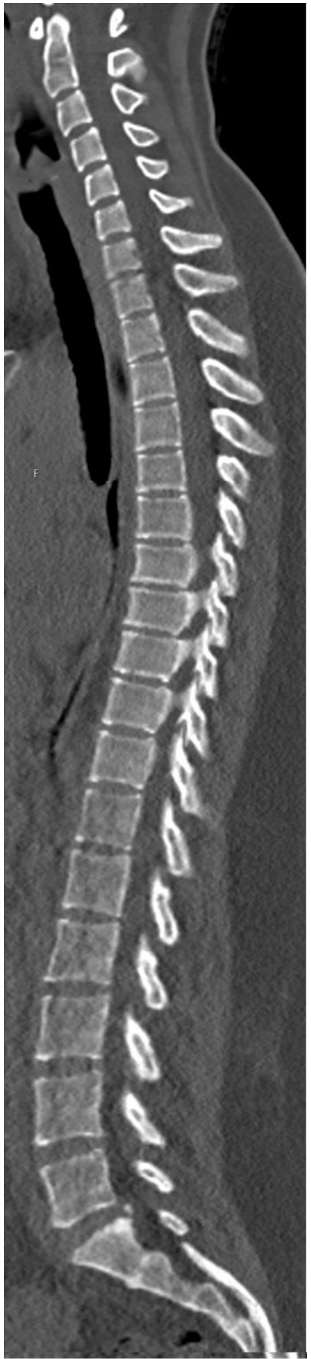

Normal disc height cervical

- 3 sagittal sections of the cervical spine from one patient

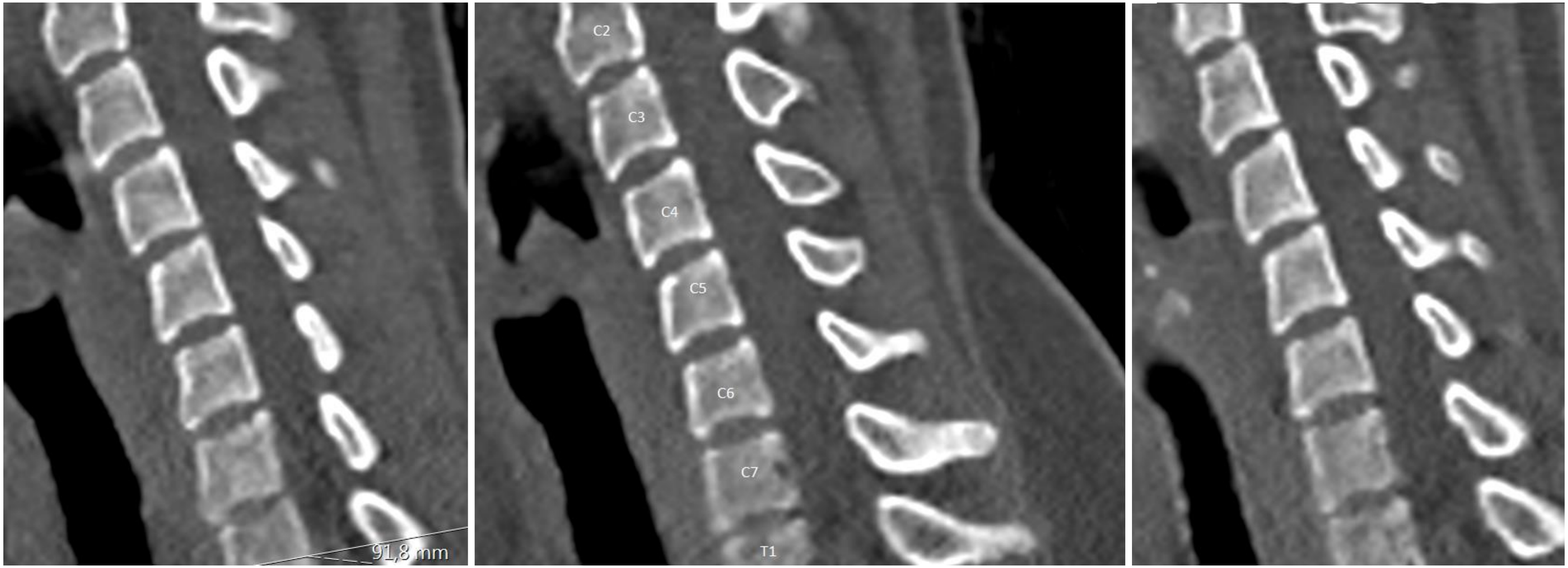

Normal disc upper thoracic

- 3 sagittal sections of the upper thoracic spine from one patient

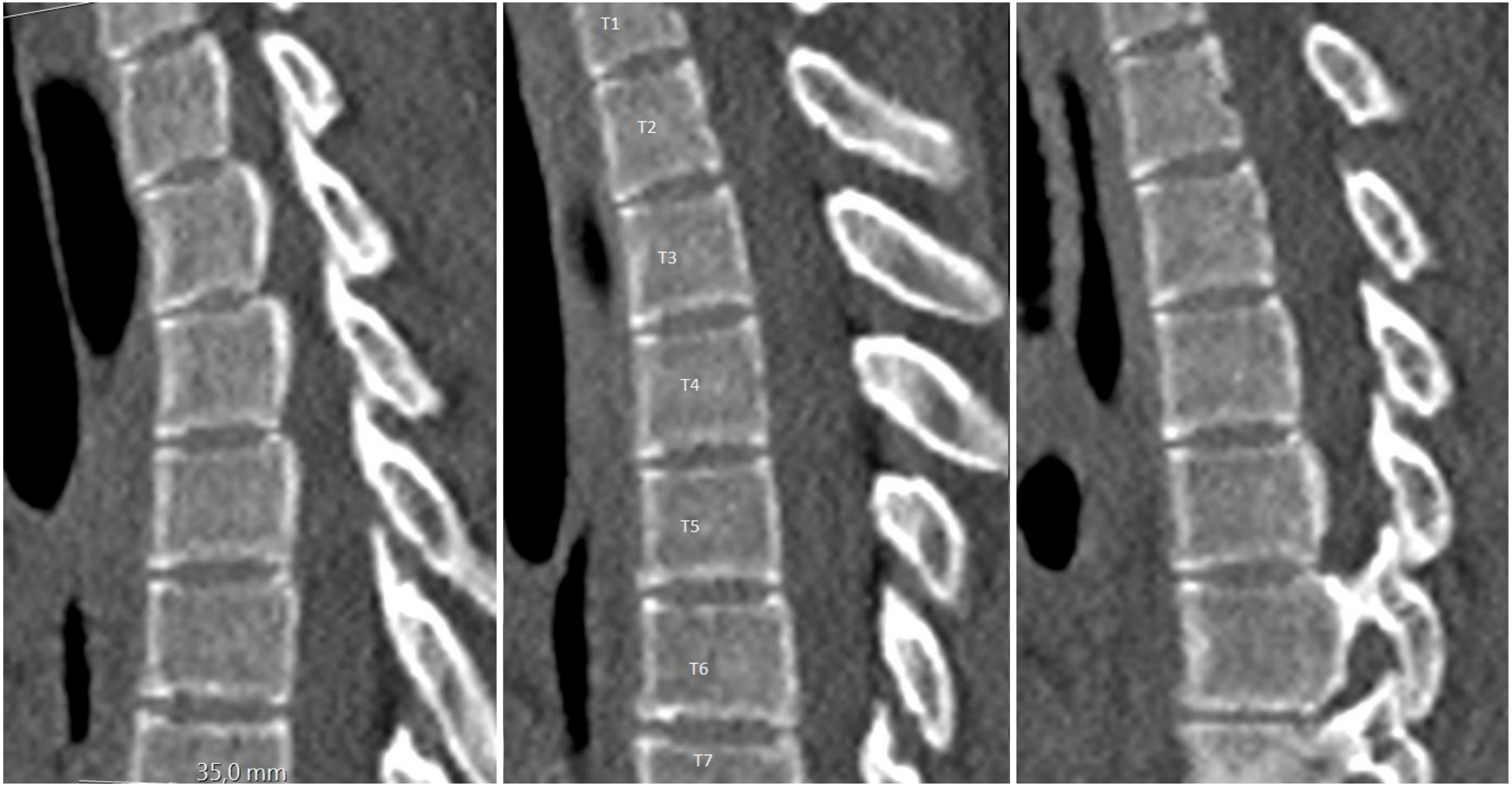

Normal disc height lower thoracic

- 3 sagittal sections of the lower thoracic spine from one patient

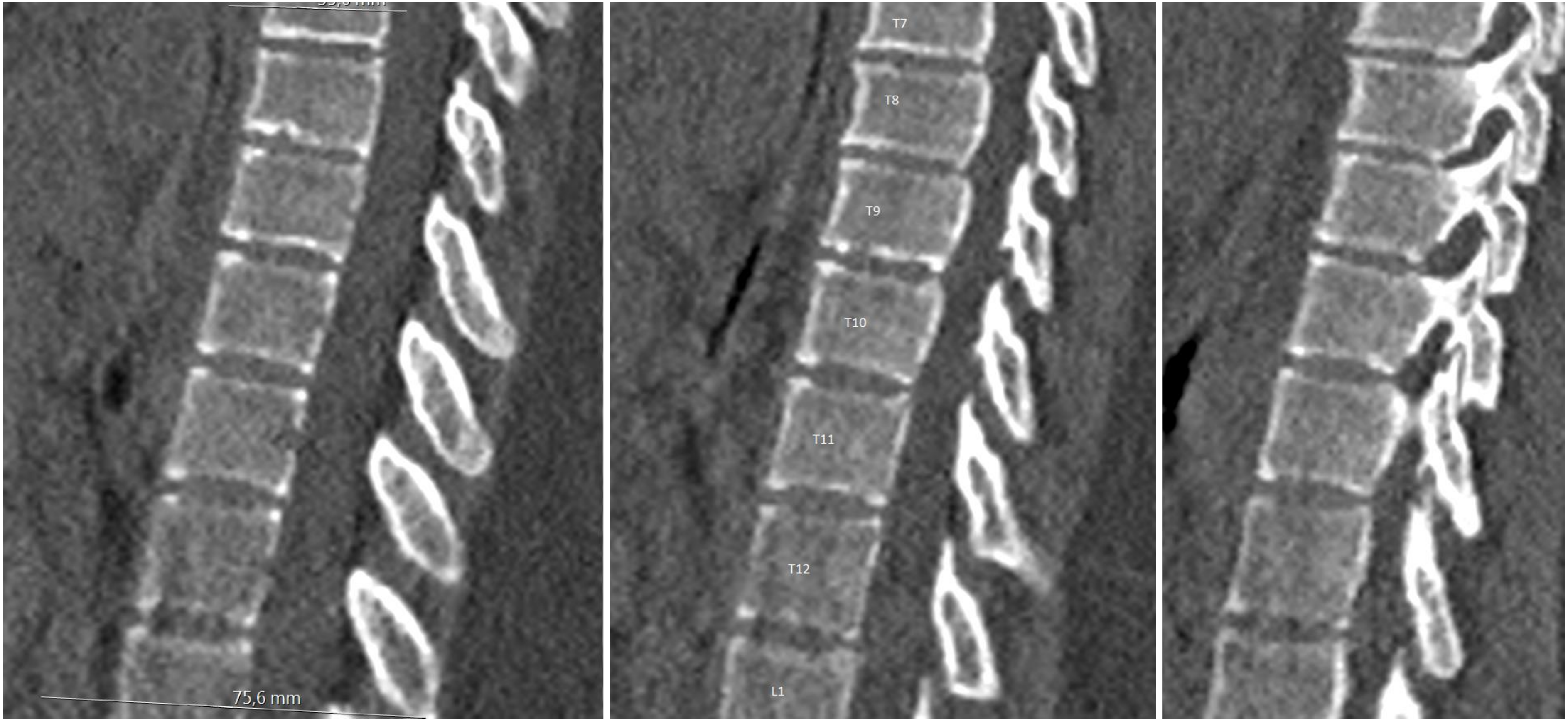

Normal disc height lumbar

- 3 sagittal sections of the lumbar spine from one patient

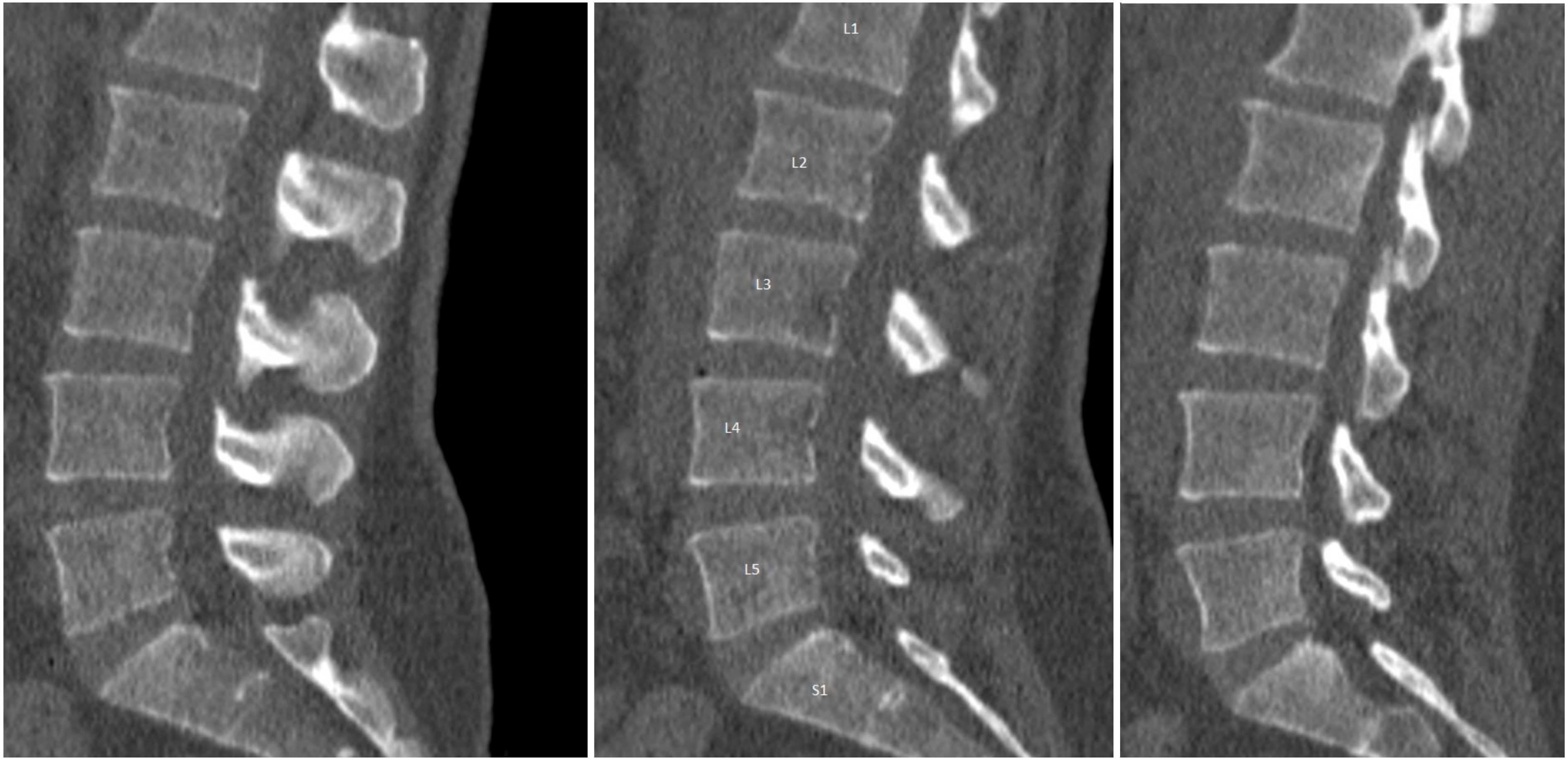

Example DSN grade 1

DSN	Definition	
0	No / possible narrowing	
**1**	**Mild narrowing**	**25 - 50%**
2	Moderate narrowing	≥ 50 - 80%
3	Severe loss of disc space	≥ 80-%

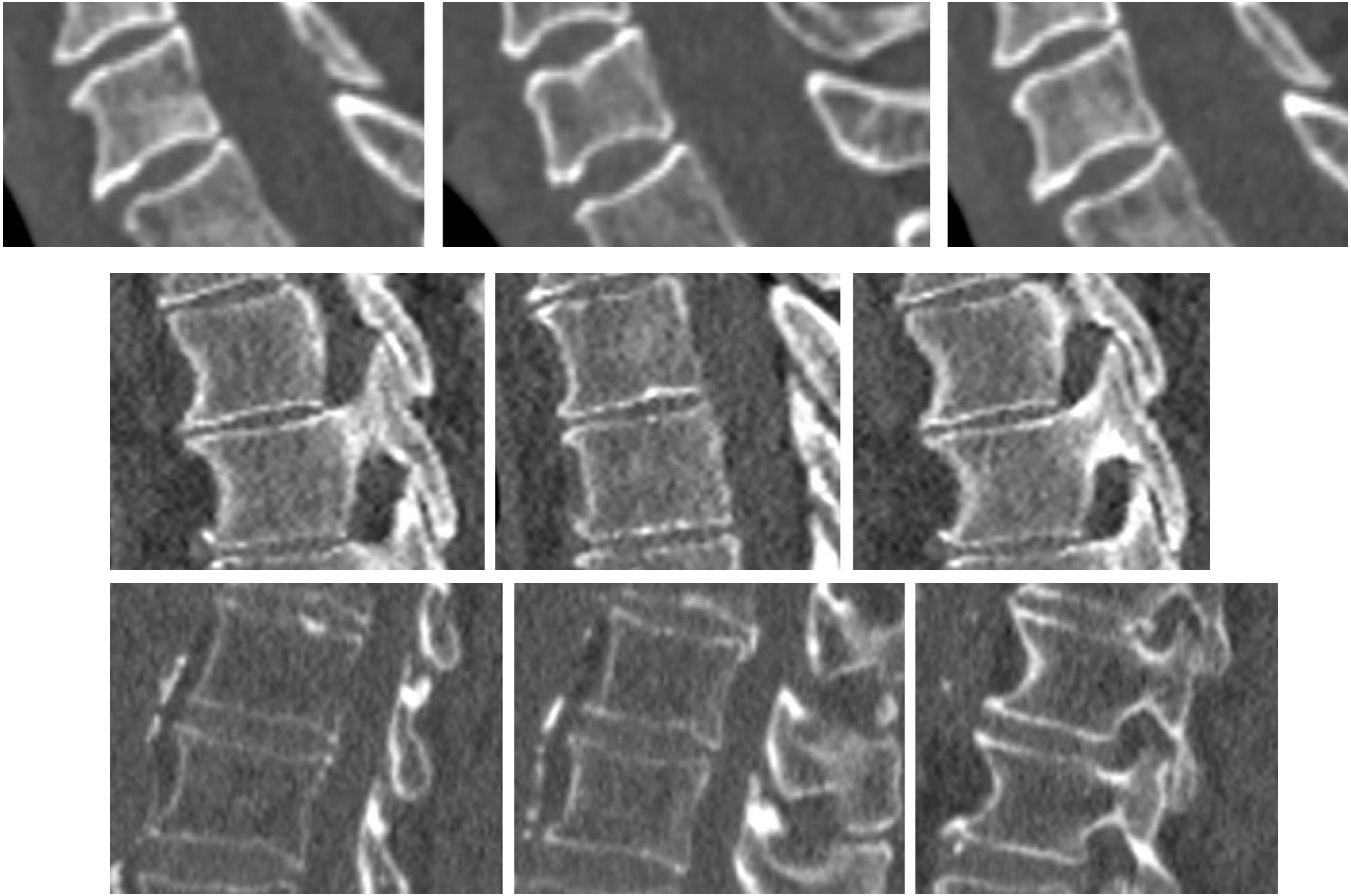

Example DSN grade 2

DSN	Definition	
0	No / possible narrowing	
1	Mild narrowing	25 - 50%
**2**	**Moderate narrowing**	**≥ 50 - 80%**
3	Severe loss of disc space	≥ 80-%

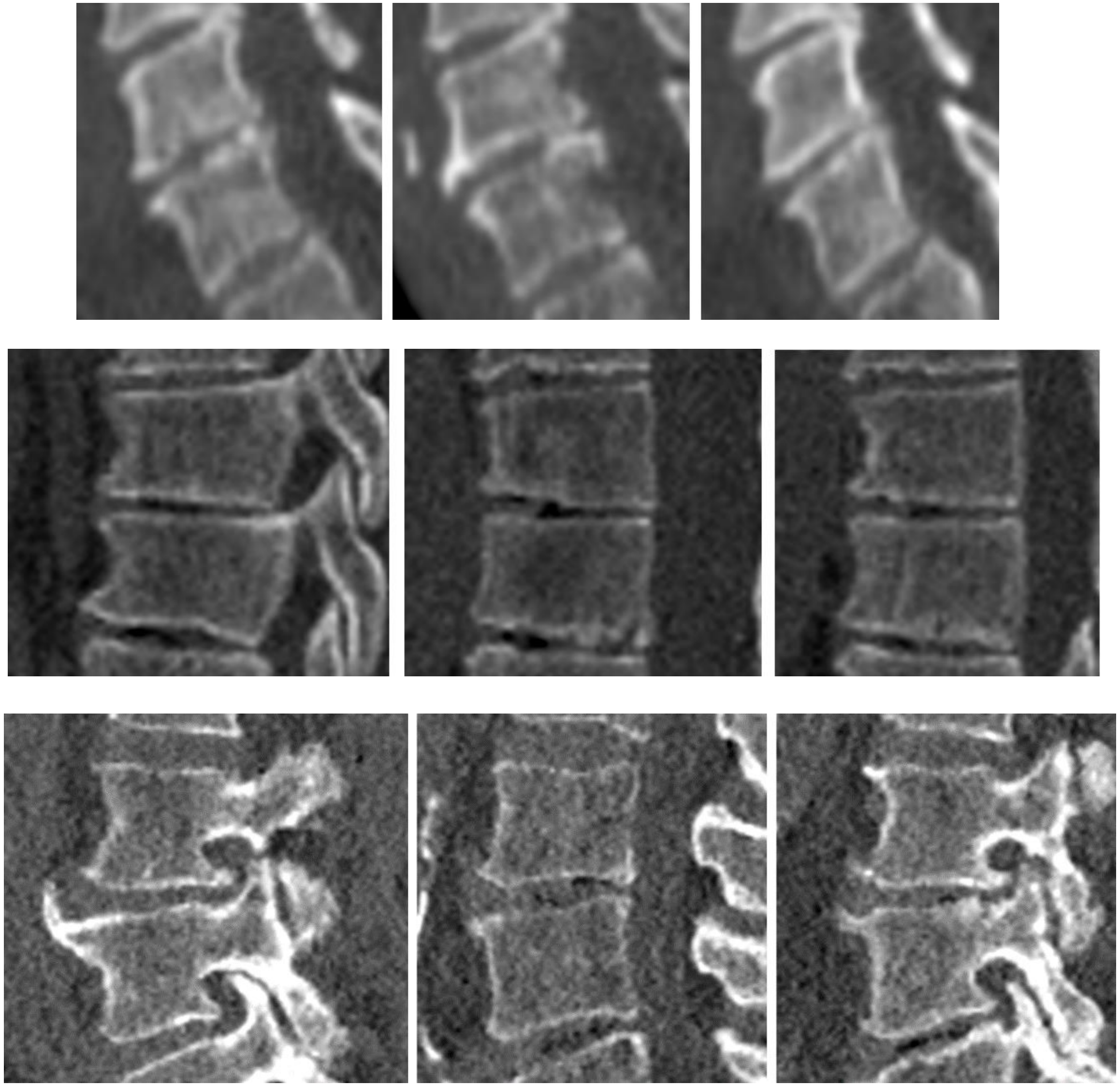

Example DSN grade 3

DSN	Definition	
0	No / possible narrowing	
1	Mild narrowing	25 - 50%
2	Moderate narrowing	≥ 50 - 80%
**3**	**Severe loss of disc space**	**≥ 80-%**

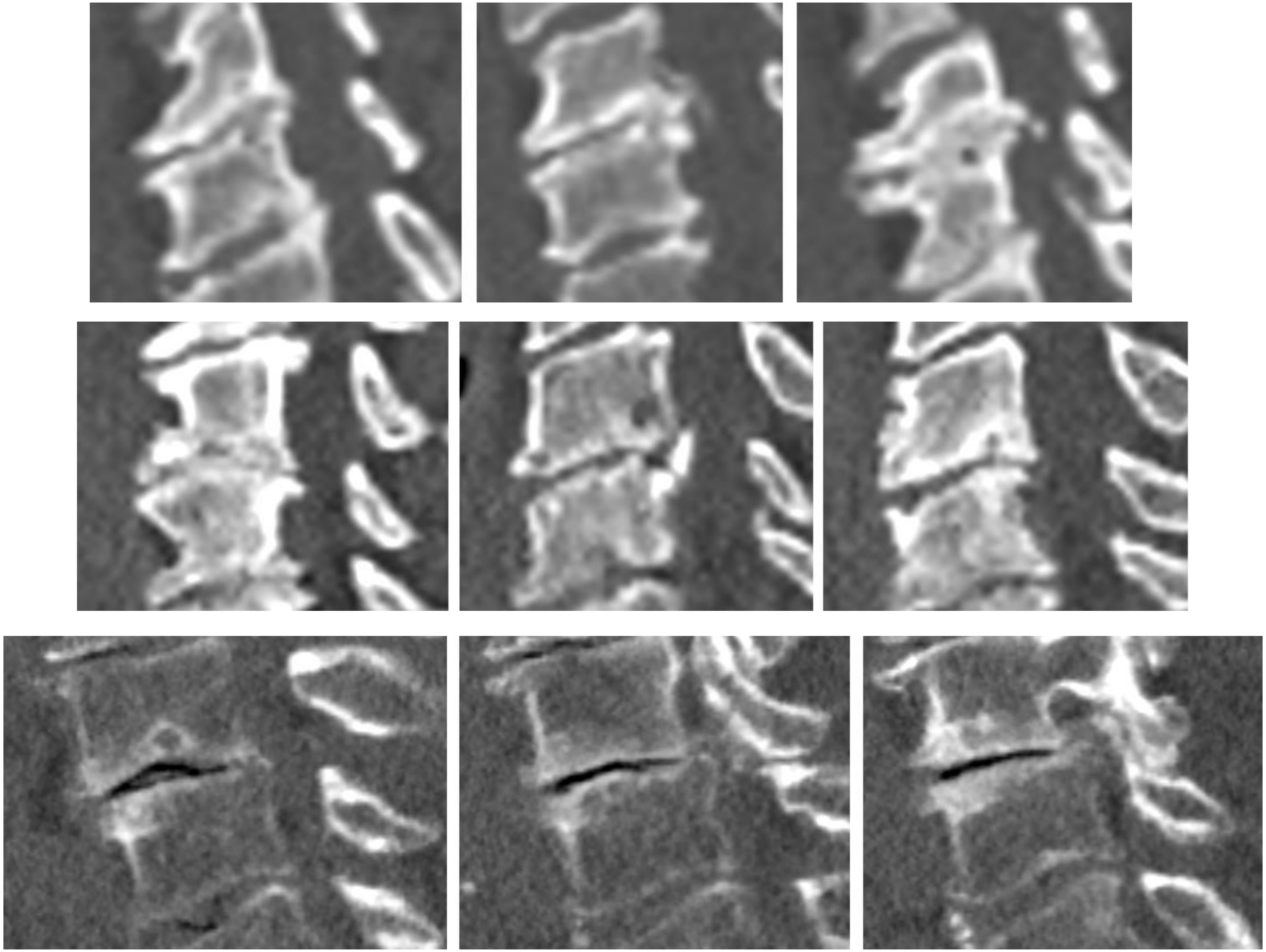

Mixed example DSN

- 3 sagittal sections of the cervical spine from one patient

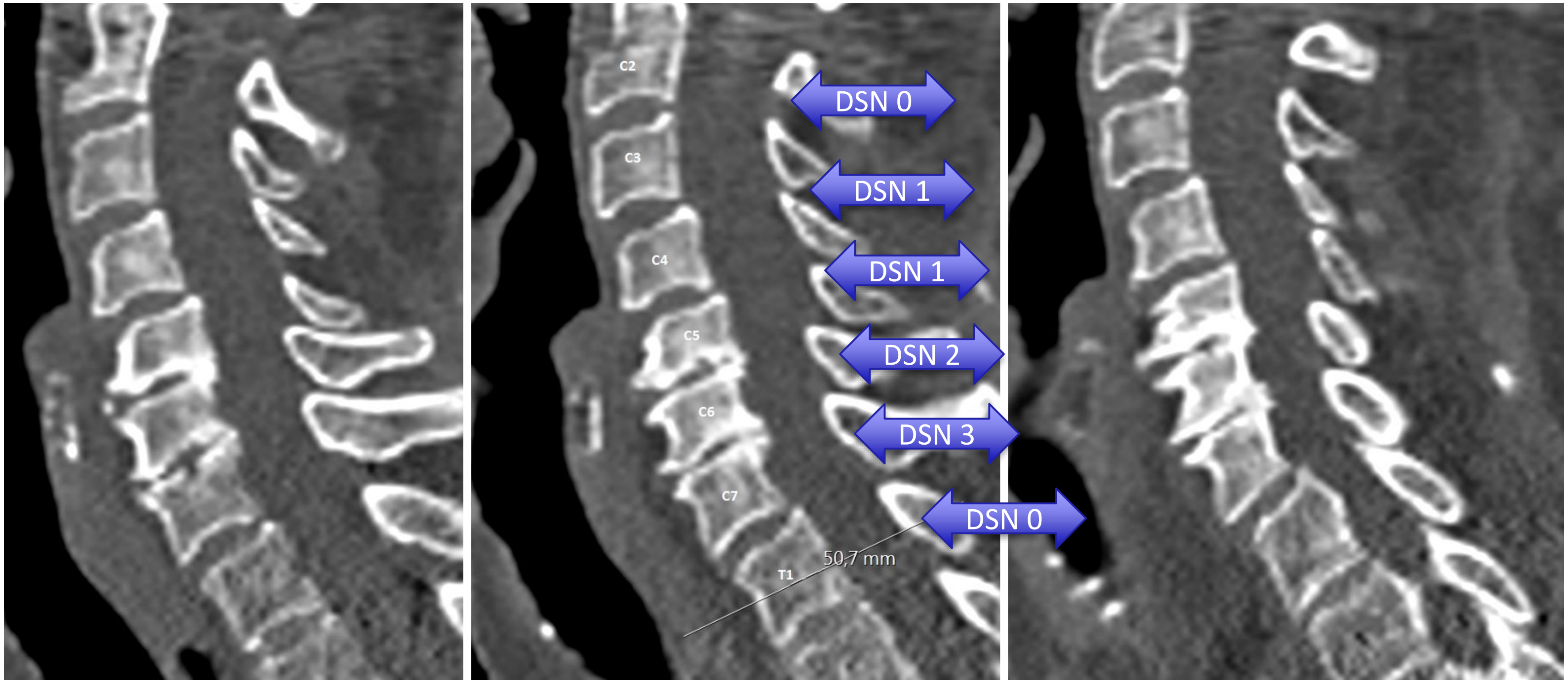

DSN	Definition	
0	No / possible narrowing	
1	Mild narrowing	25 - 50%
2	Moderate narrowing	≥ 50 - 80%
3	Severe loss of disc space	≥ 80-%

Mixed example DSN

- 4 sagittal sections of upper region of the thoracic spine from one patient
  - Normally the thoracic spine is scored as one, we now only show the upper region

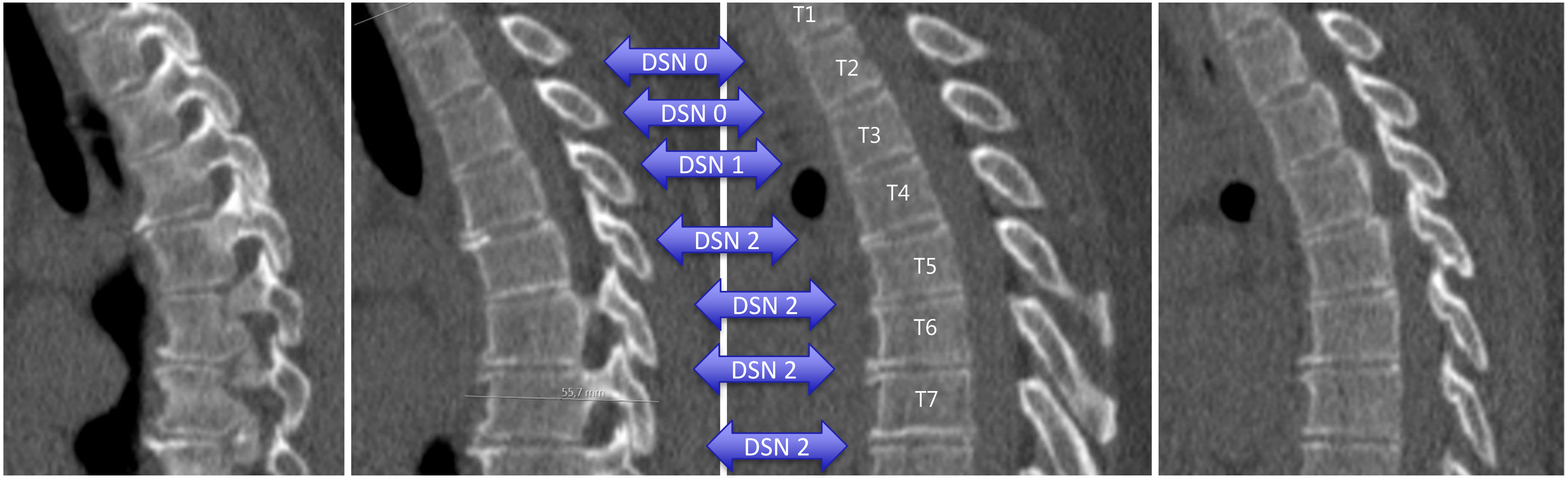

DSN	Definition	
0	No / possible narrowing	
1	Mild narrowing	25 - 50%
2	Moderate narrowing	≥ 50 - 80%
3	Severe loss of disc space	≥ 80-%

Mixed example DSN

- 3 sagittal sections of the lower section of the lumbar spine from one patient
  - Normally the lumbar spine is scored as one, we now only show the lower region

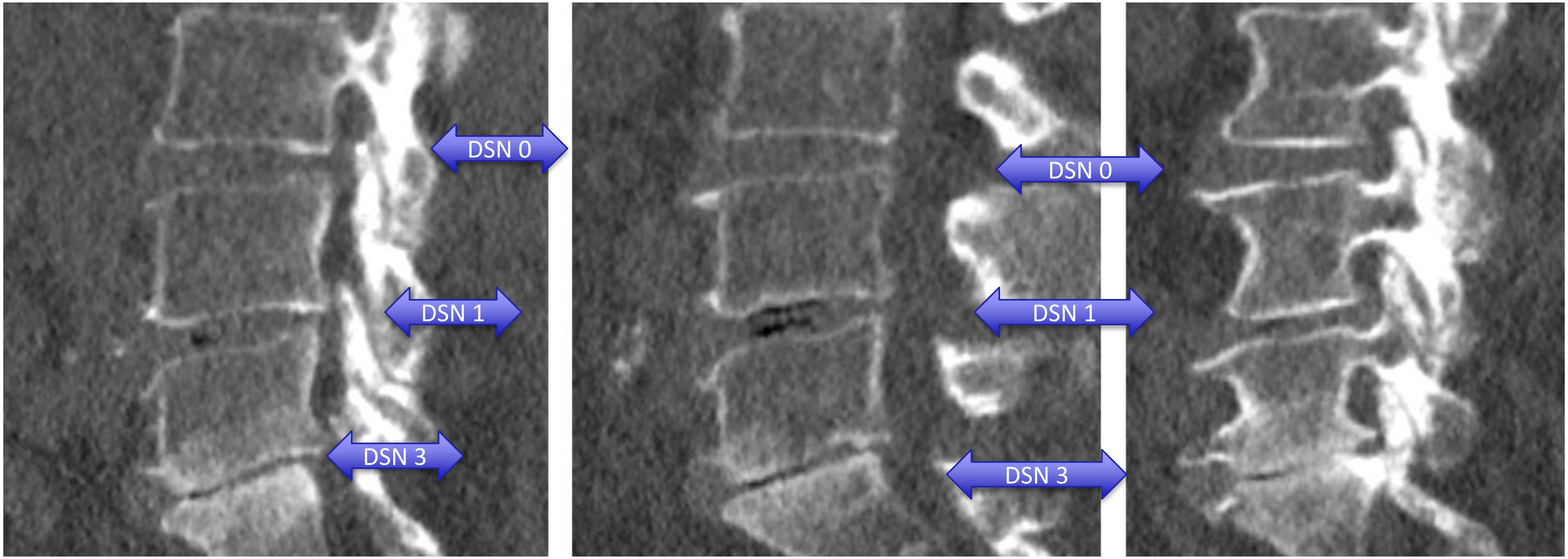

DSN	Definition	
0	No / possible narrowing	
1	Mild narrowing	25 - 50%
2	Moderate narrowing	≥ 50 - 80%
3	Severe loss of disc space	≥ 80-%

Sclerosis and endplate irregularity

SCIR	Definition
0	No sclerosis or irregularity
1	Visible sclerosis and/or irregularity

- Sclerosis visible in the vertebral body
- Endplate irregularity
  - Any visible irregularities in both endplates (cranial/caudal) are scored
    - Schmorl's nodules
    - Attrition (due to bone-to-bone contact)
- Examples show 3 sagittal slices of the same patient next to each other

Sclerosis without irregularity

SCIR	Definition
0	No sclerosis or irregularity
**1**	**Visible sclerosis** and/or irregularity

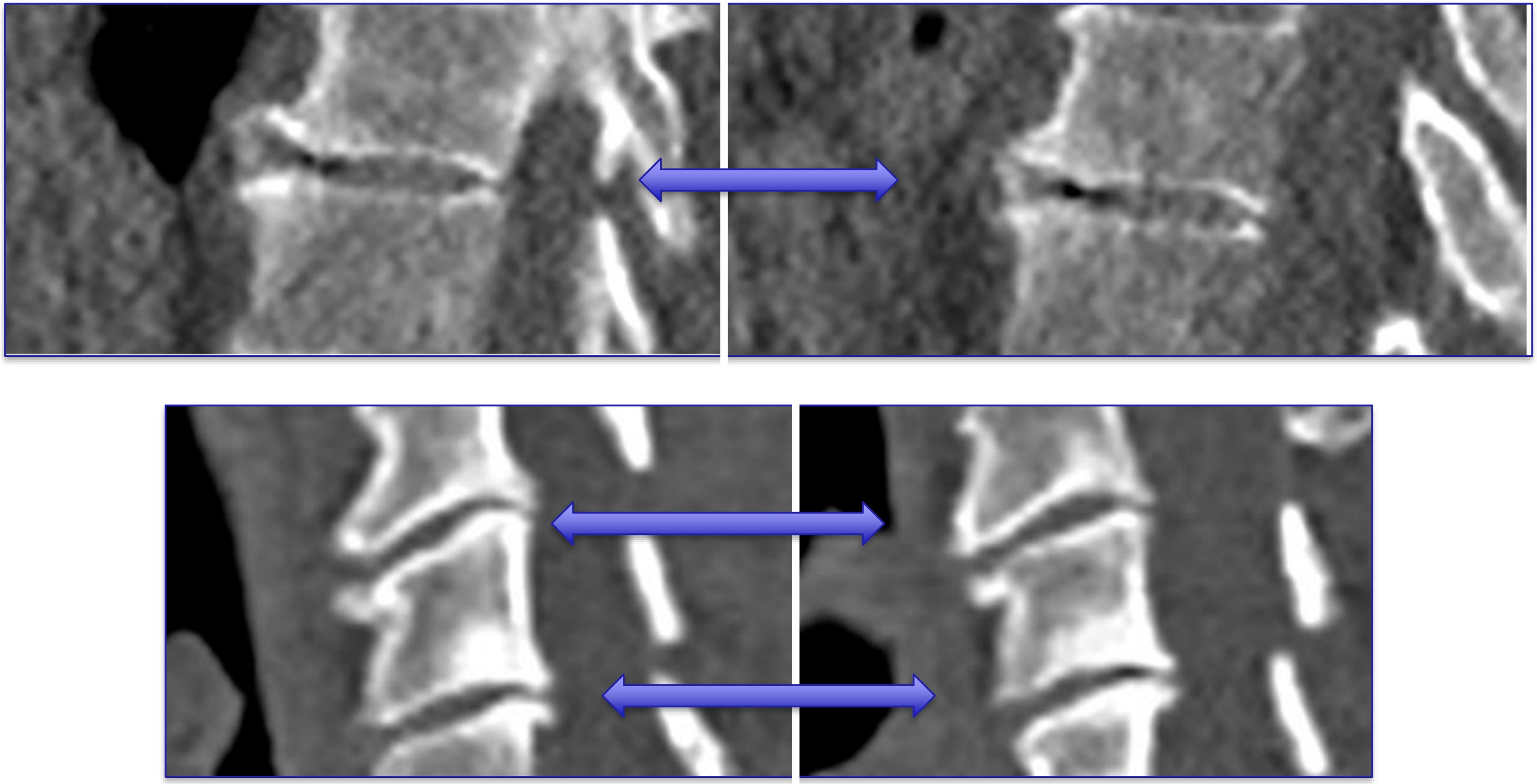

Endplate irregularity without sclerosis

SCIR	Definition
0	No sclerosis or irregularity
**1**	Visible sclerosis and/or **irregularity**

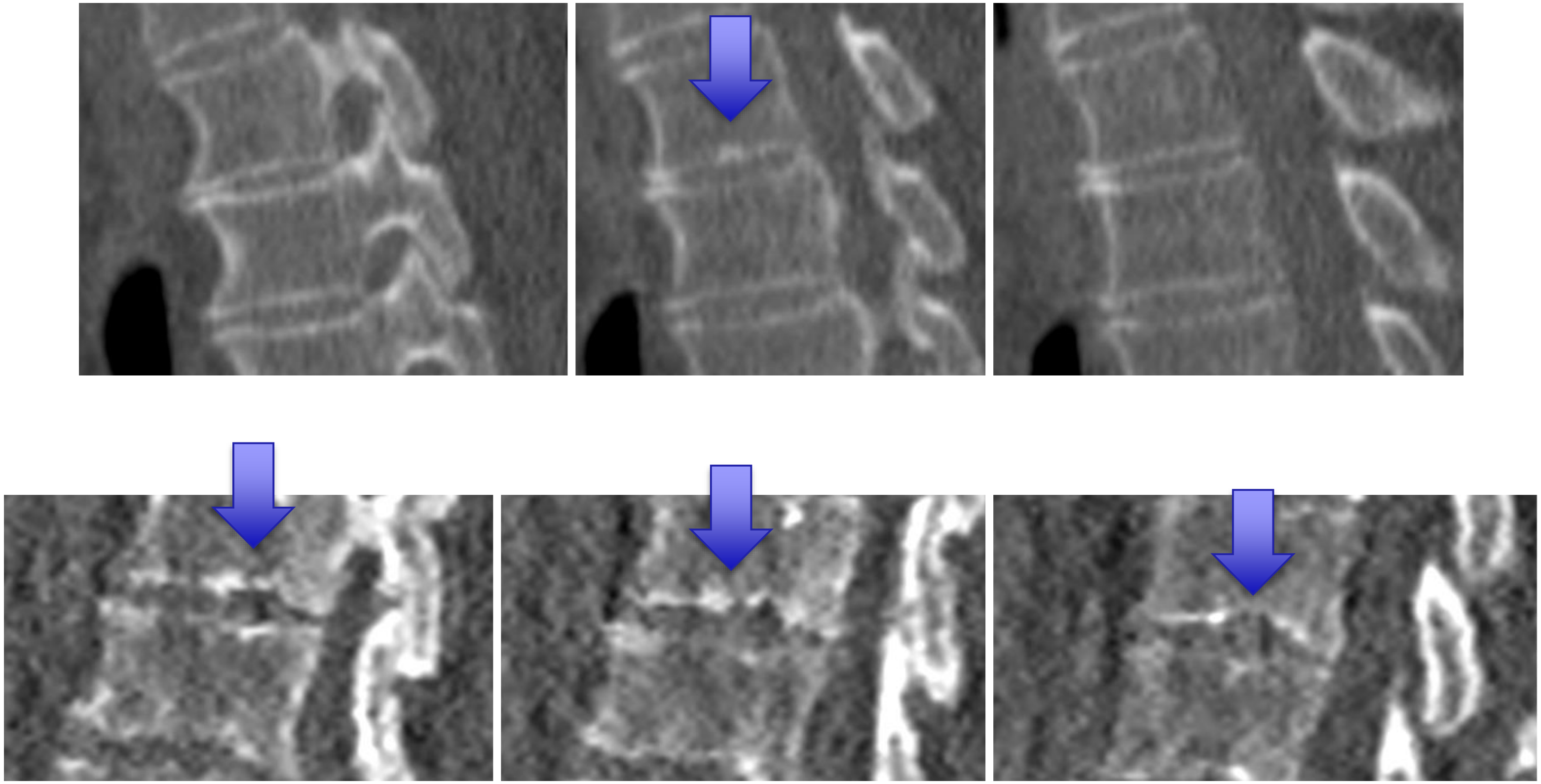

Irregularity with sclerosis

SCIR	Definition
0	No sclerosis or irregularity
**1**	**Visible sclerosis and/or irregularity**

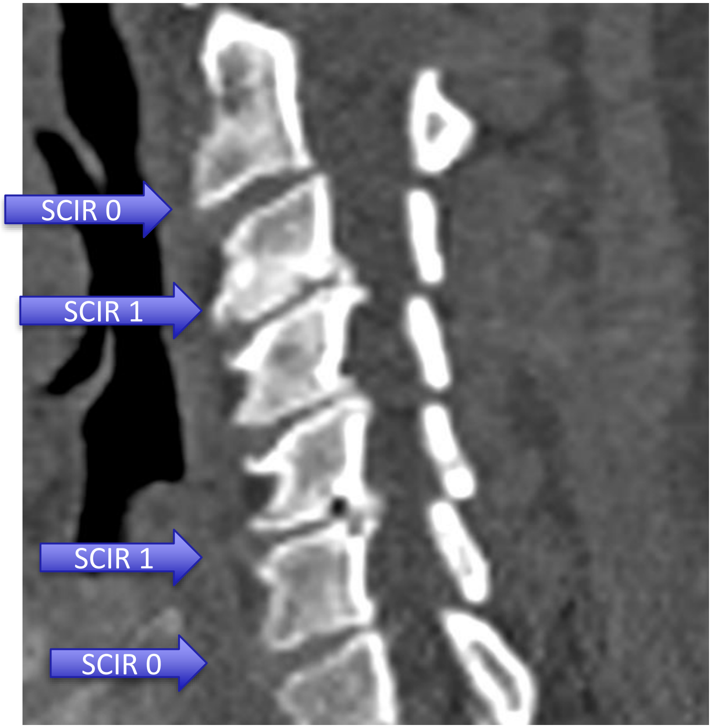

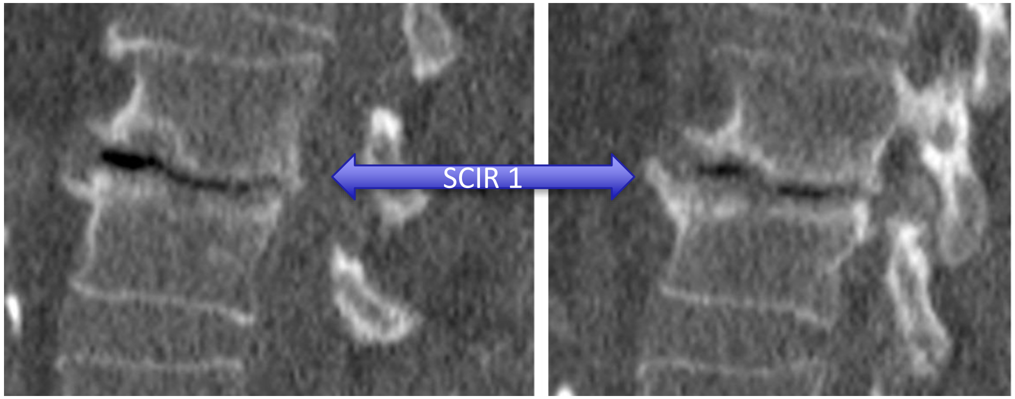

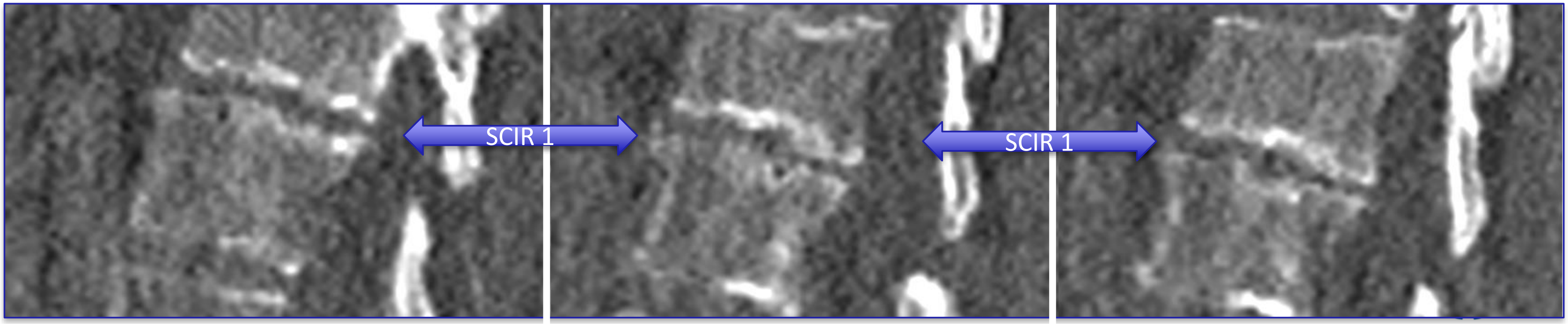

Vertebral osteophytes

- Score osteophytes based on sagittal image
- Score all osteophytes (also flowing/bridges)
- Consider ventral and dorsal osteophytes
- Do not score loose calcified bodies

OST	Definition
0	No osteophyte
1	Lipping / small osteophyte
2	Clear moderate osteophyte
3	Clear large osteophyte

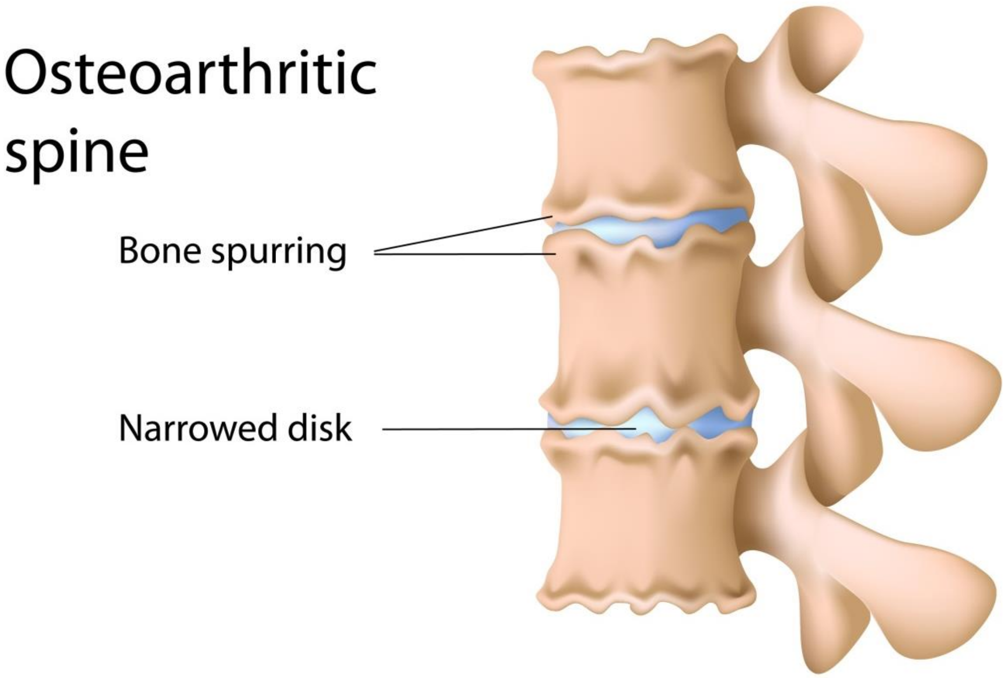

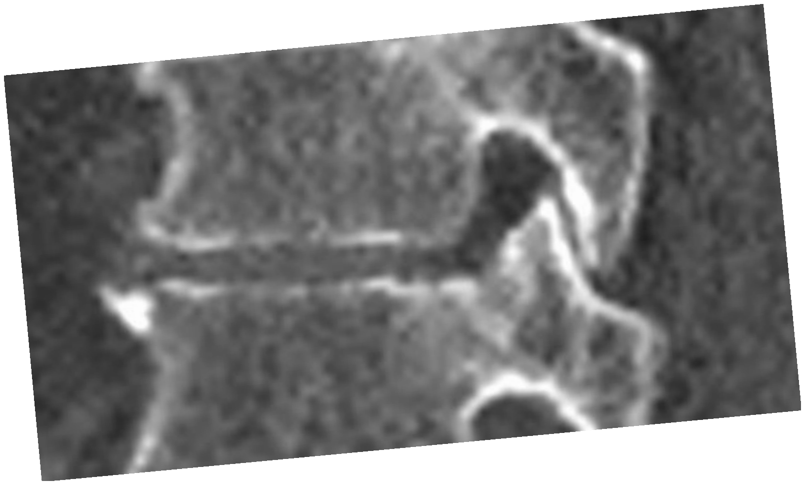

Illustration from Shutterstock

Calcific enthesopathy

- Do not score calcific enthesopathy, calcified structures not attached to the vertebral body (red arrows), only osteophytes (white arrows)
- Example shows 3 sagittal slices of the same patient next to each other

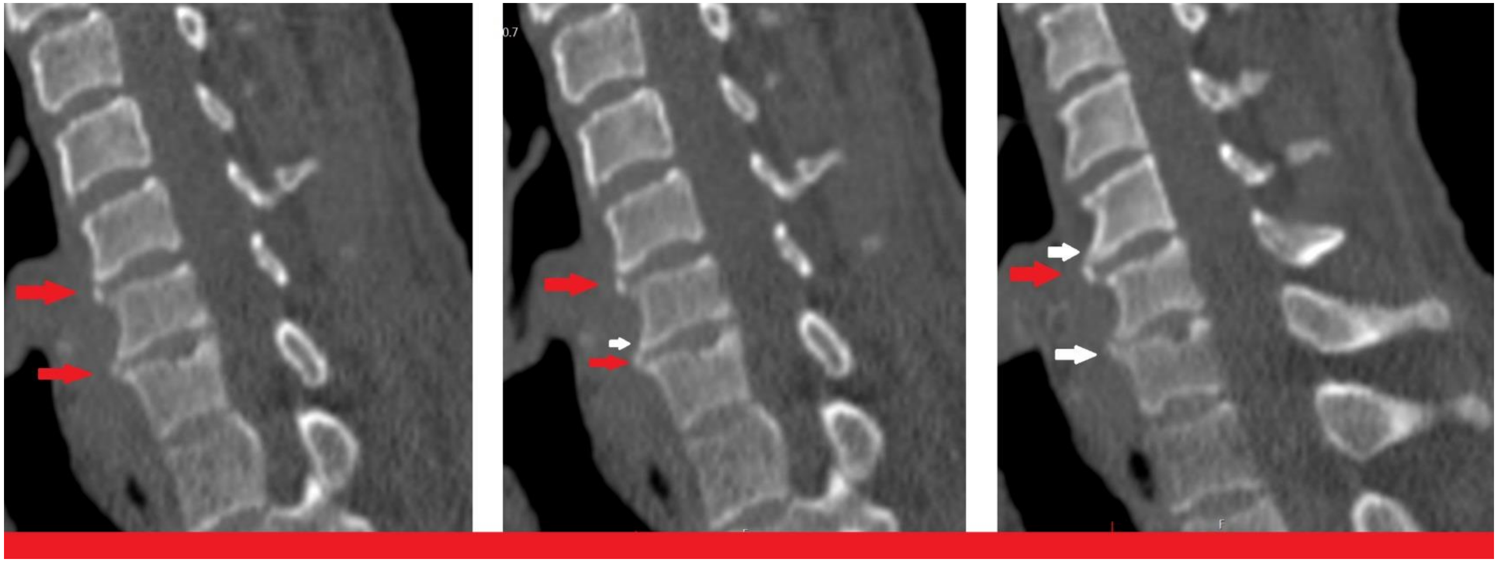

Vertebral osteophyte 0

Scoring is done in sagittal view, coronal view is given in examples to increase comprehension of 3d process

OST	Definition
**0**	**No osteophyte**
1	Lipping / small osteophyte
2	Clear moderate osteophyte
3	Clear large osteophyte

Consider anterior and posterior osteophytes

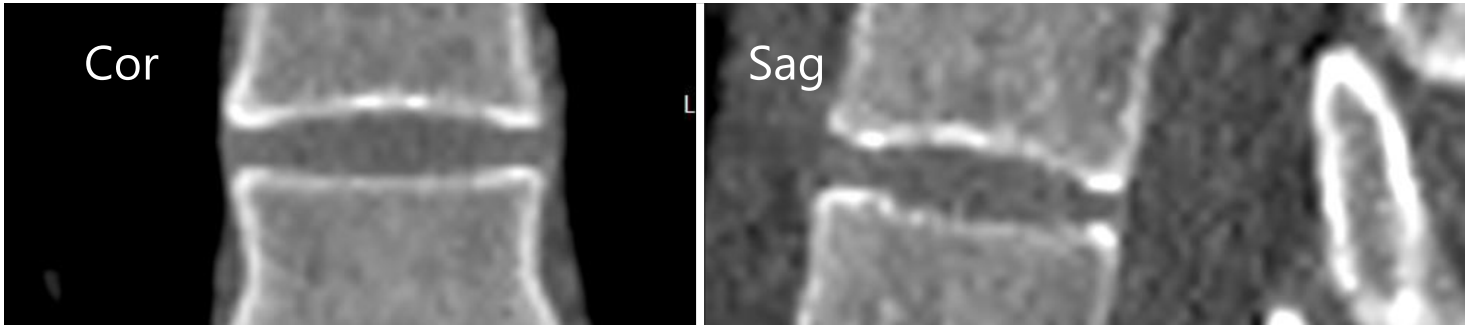

Vertebral osteophyte 1

OST	Definition
0	No osteophyte
**1**	**Lipping / small osteophyte**
2	Clear moderate osteophyte
3	Clear large osteophyte

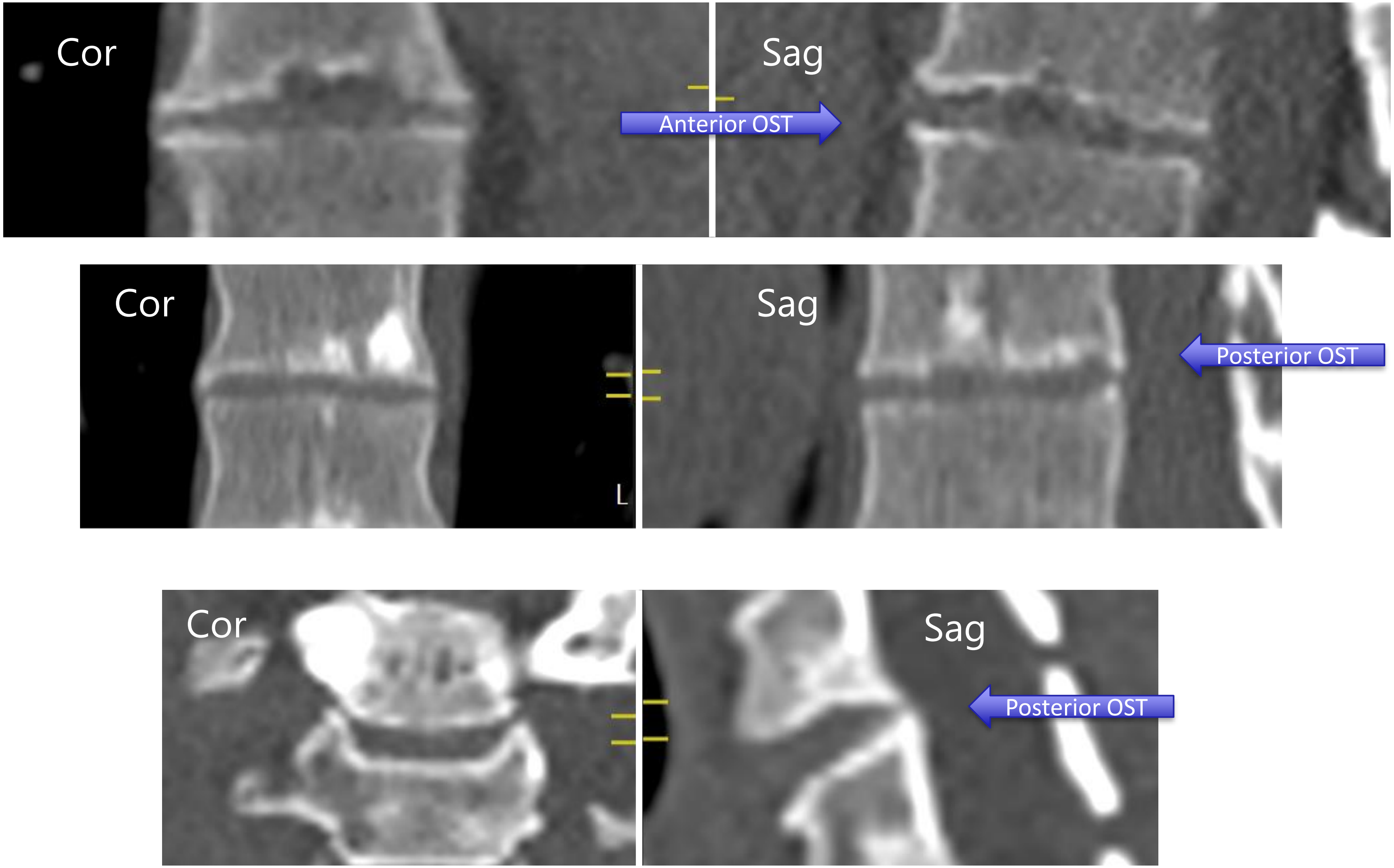

Vertebral osteophyte 2

OST	Definition
0	No osteophyte
1	Lipping / small osteophyte
**2**	**Clear moderate osteophyte**
3	Clear large osteophyte

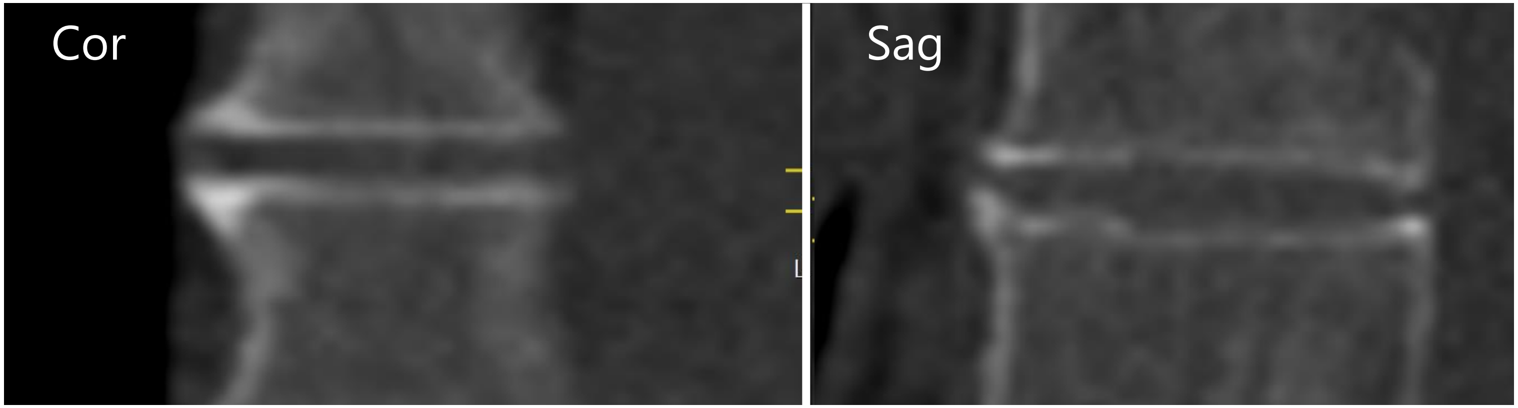

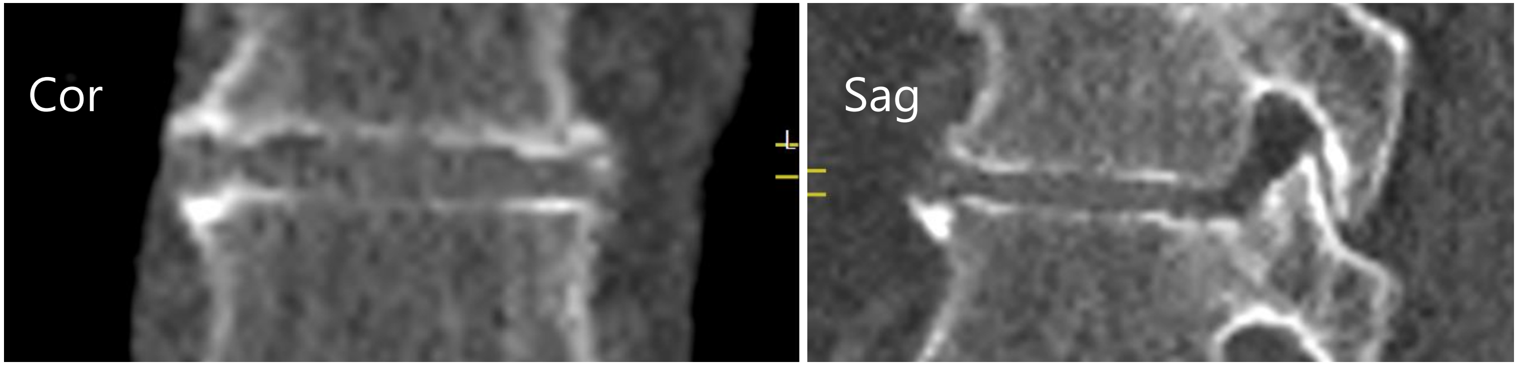

Vertebral osteophyte 3

OST	Definition
0	No osteophyte
1	Lipping / small osteophyte
2	Clear moderate osteophyte
**3**	**Clear large osteophyte**

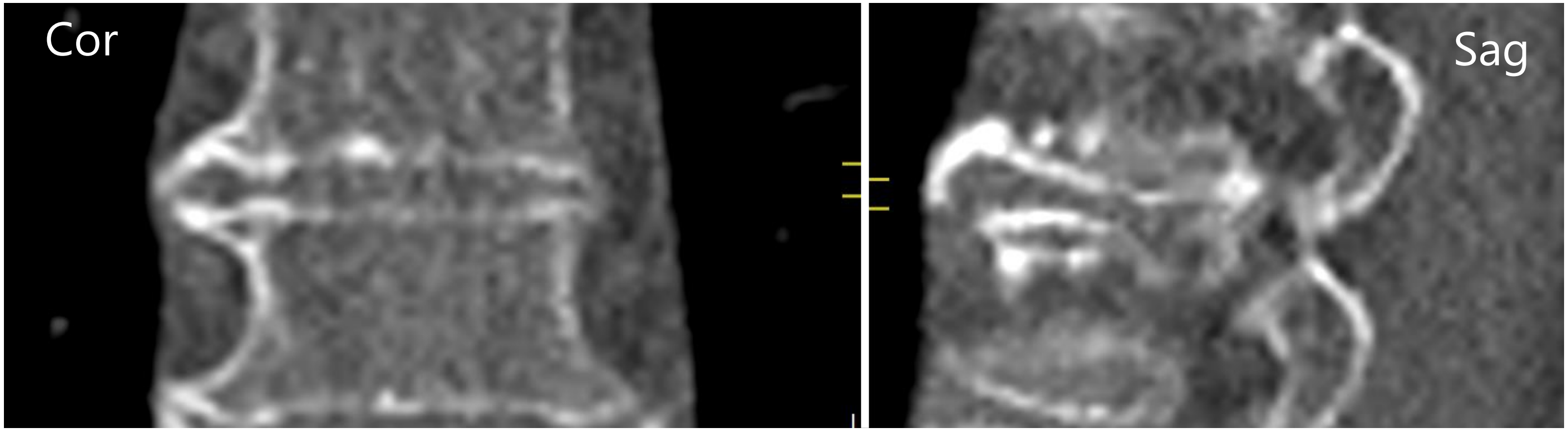

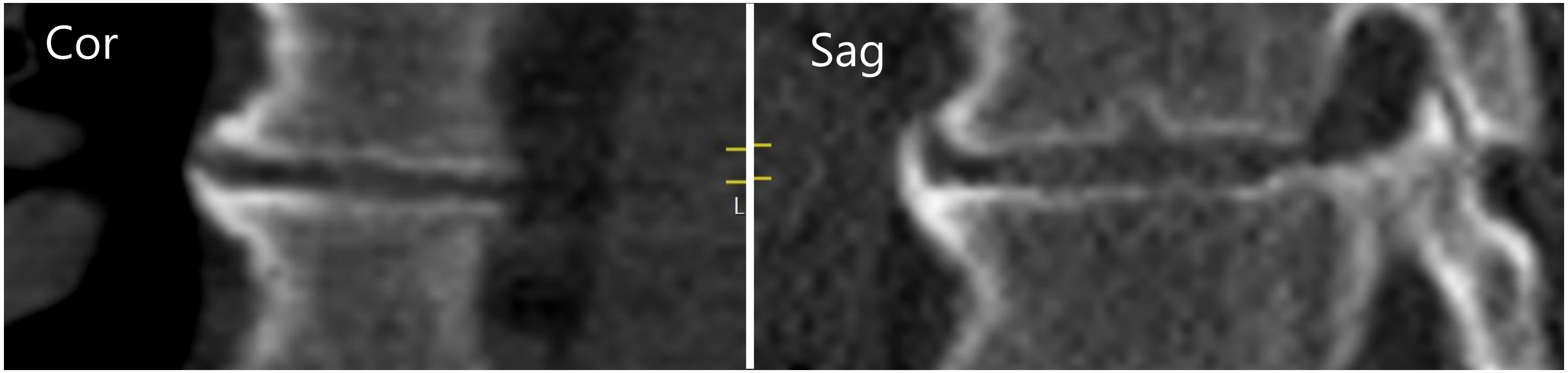

Degenerative disc disease (DDD)

- Only the grades of the two most degenerated levels per section (cervical/thoracic/lumbar) will be registered

- To determine the most degenerated levels:

  - Assess DSN of all IVD levels and select two levels with highest DSN score

  - When more then two levels qualify:
    - Assess endplate irregularity and sclerosis of the levels with the highest DSN score
    - Select two levels with highest DSN and SCIR

  - When more then two levels qualify:
    - Assess osteophytosis of the levels with the highest DSN score and highest SCIR
    - Select two levels with highest DSN, SCIR and OST

  - When two levels show exactly the same semi-quantitative scores:
    - Choose most affected level based on qualitative assessment of degeneration from those two levels

All degenerative features are scored, also if they might be "physiological" considering subjects age!

Mixed example 1 (DSN SCIR OST)

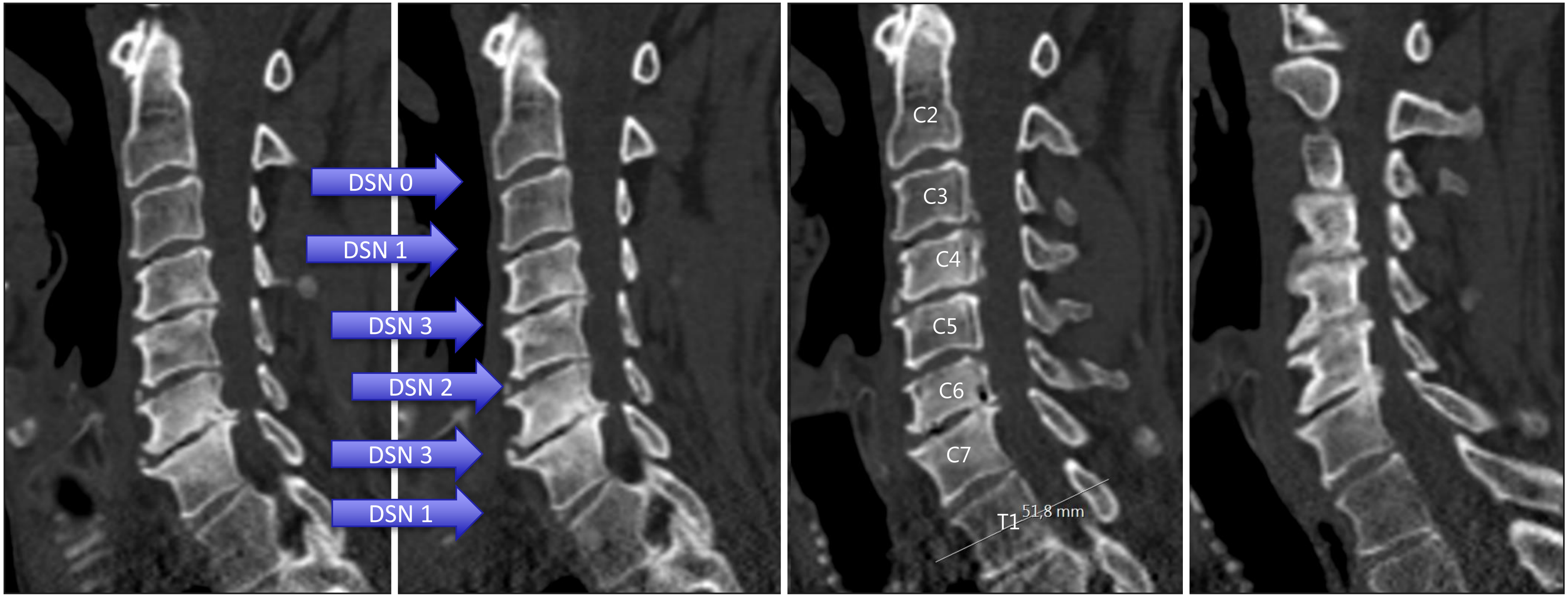

- Worst Level C6-7: Based on DSN 3
- Second worst C4-5: Based on DSN 3

Mixed example 1 (DSN SCIR OST)

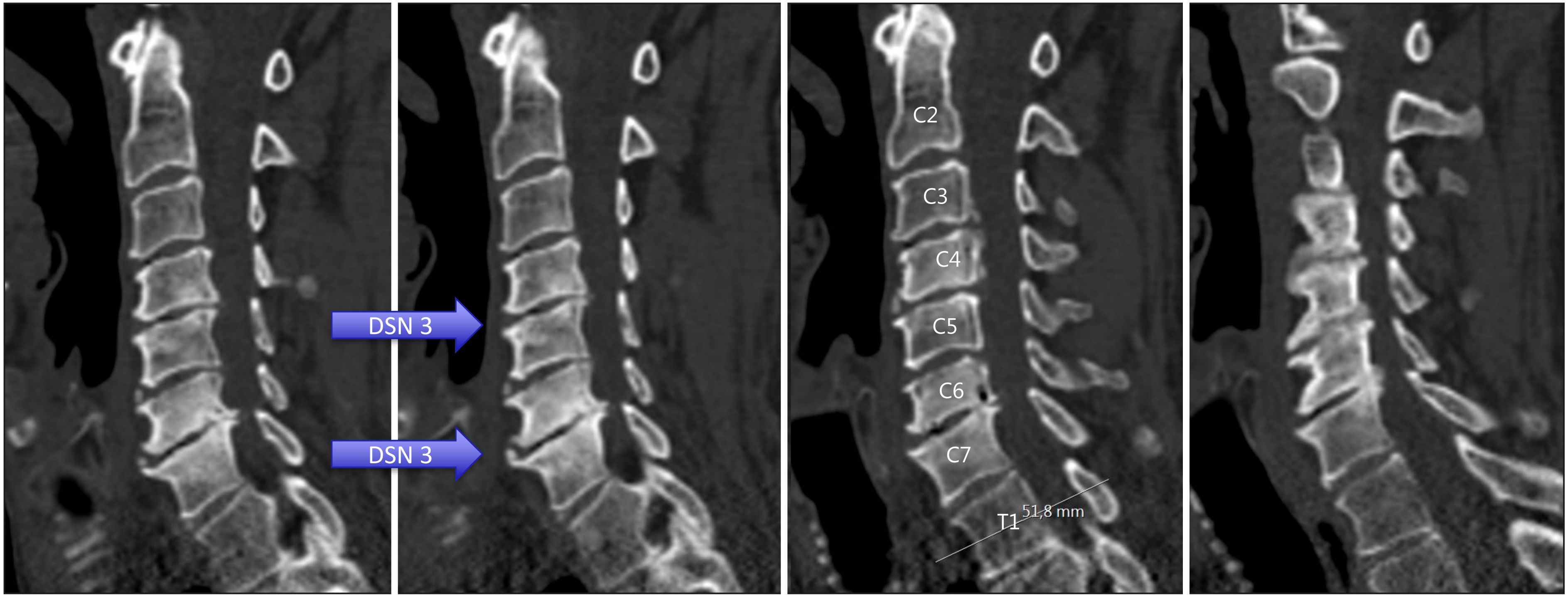

- Worst Level C6-7: Based on DSN 3
- Second worst C4-5: Based on DSN 3

Mixed example 1 (DSN SCIR OST)

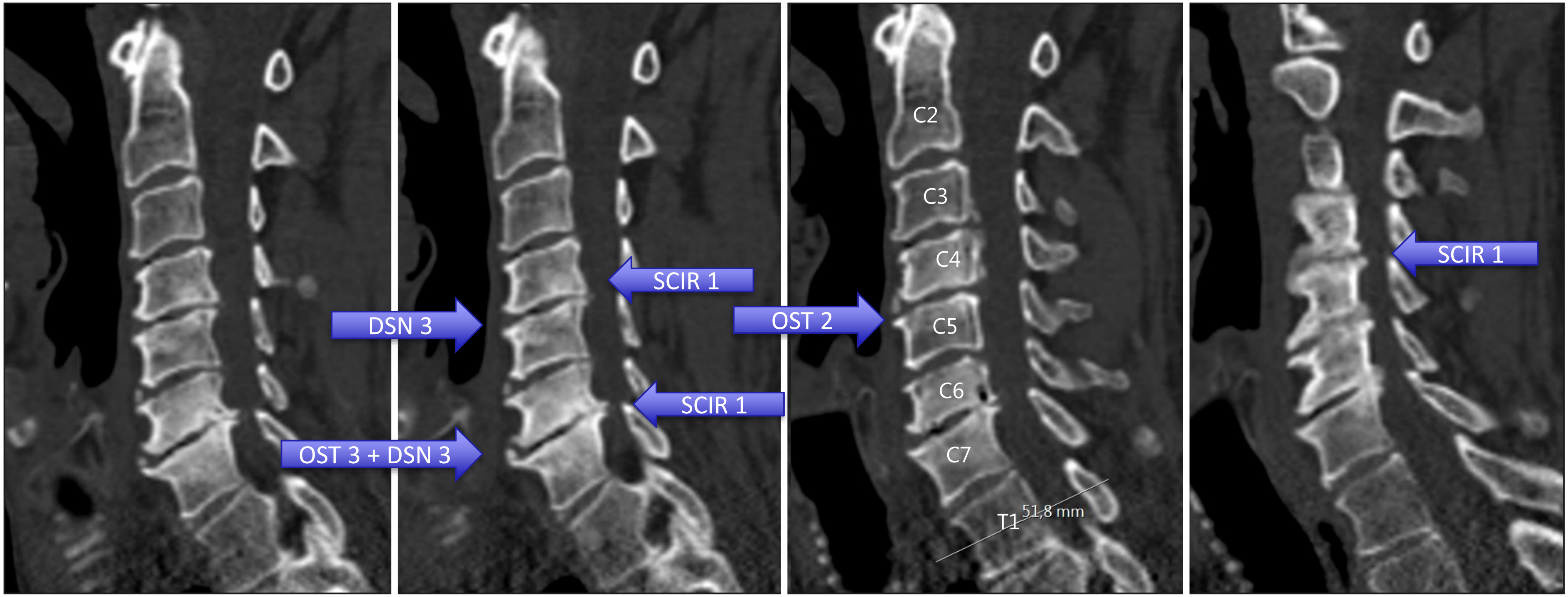

- Worst Level C6-7 (DSN 3, OST 3, SCIR 1)
- Second worst C4-5 (DSN 3, OST 2, SCIR 1)

Mixed example 2 (DSN SCIR OST)

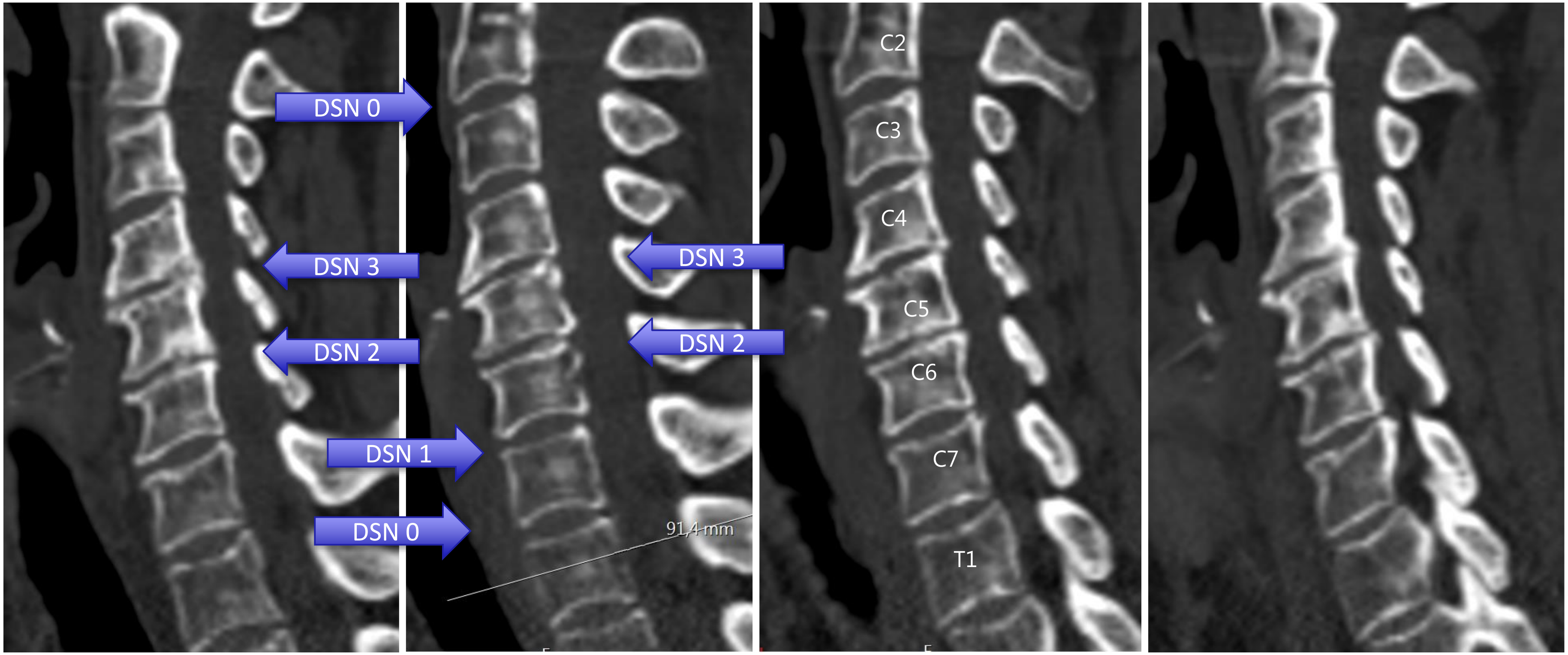

- Worst Level C4-5: Based on DSN 3
- Second worst C5-6: Based on DSN 2

Mixed example 2 (DSN SCIR OST)

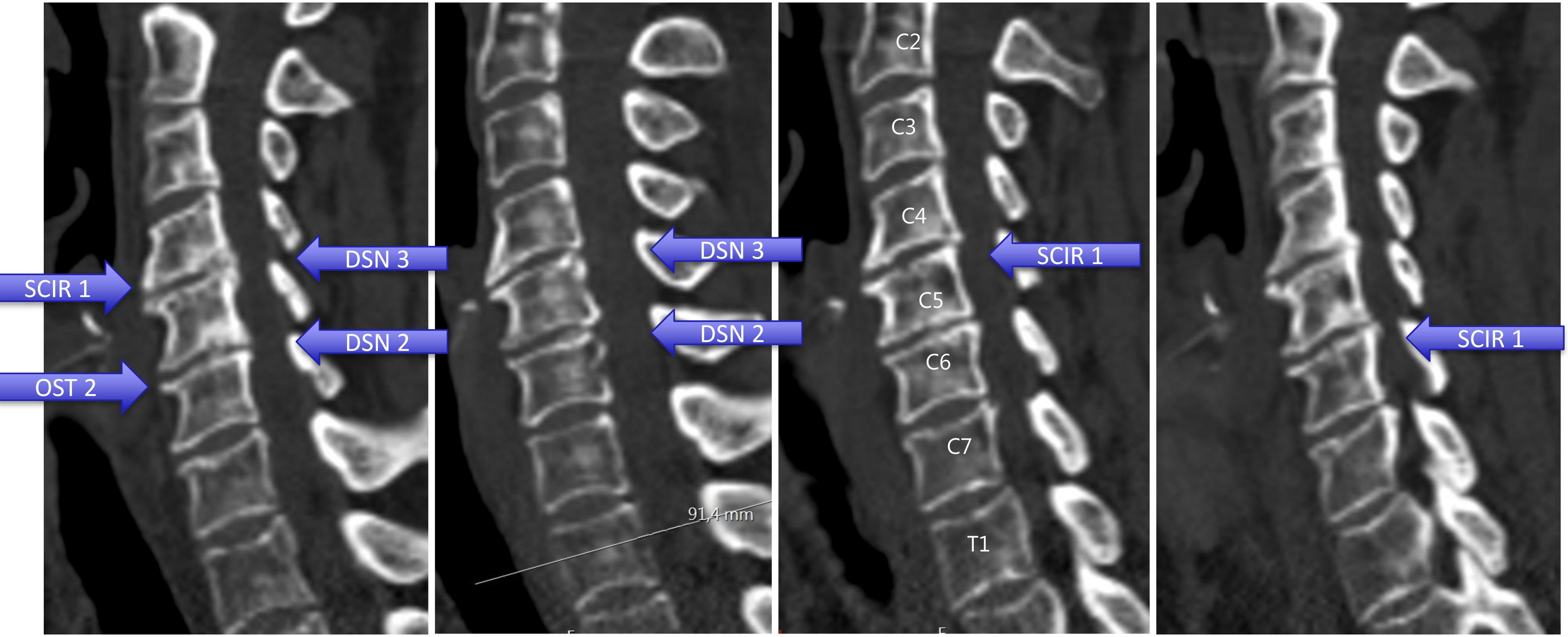

- Worst Level C4-5 (DSN 3, SCIR 1, OST 2)
- Second worst C5-6 (DSN 2, SCIR 1, OST 1)

Mixed example 3 (DSN SCIR OST)

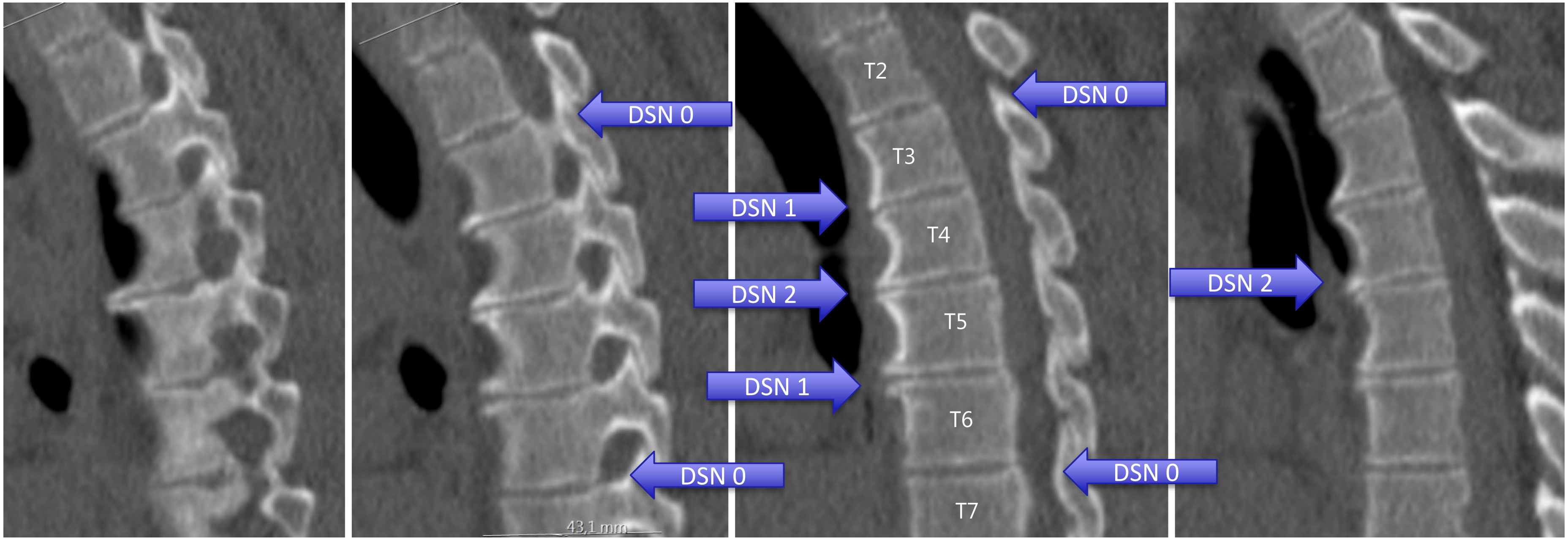

- Because of static viewing, only upper thoracic (T1-T7) is shown
- Worst Level T4-T5: Based on DSN 2
- Second worst T3-T4 or T5-T6: Based on DSN 1

Mixed example 3 (DSN SCIR OST)

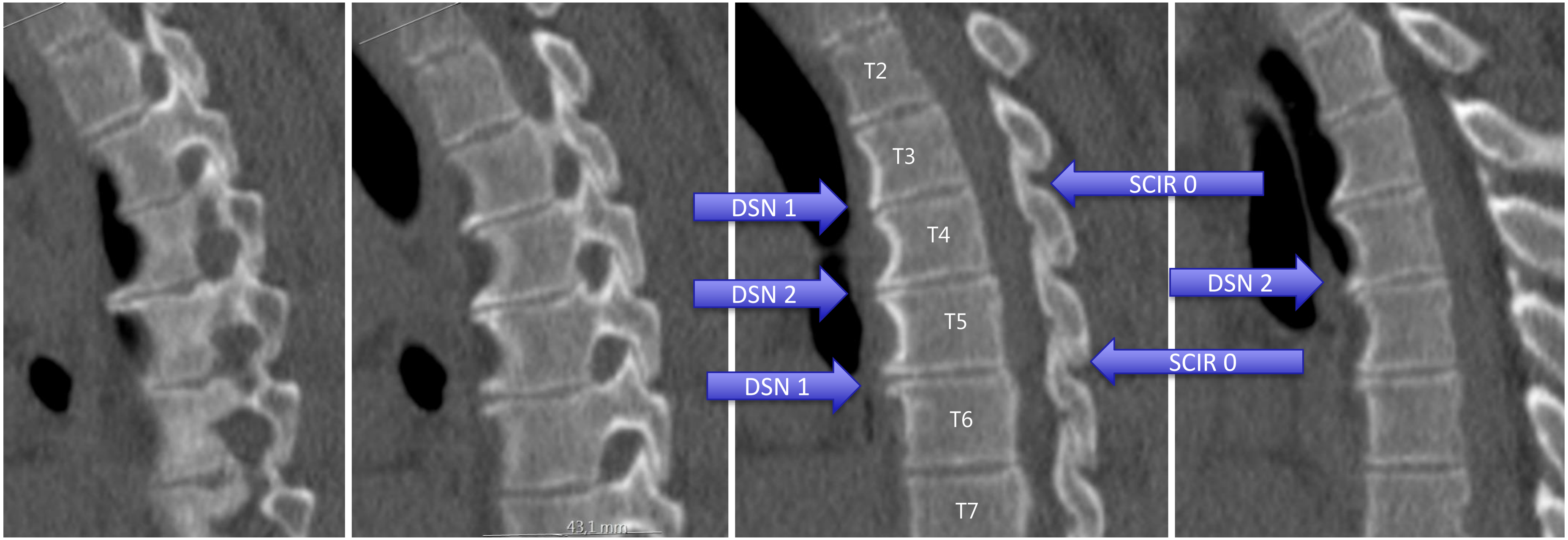

- Because of static viewing, only upper thoracic (T1-T7) is shown
- Worst Level T4-T5: Based on DSN 2
- Second worst T3-T4 or T5-T6: Based on DSN 1, SCIR 0

Mixed example 3 (DSN SCIR OST)

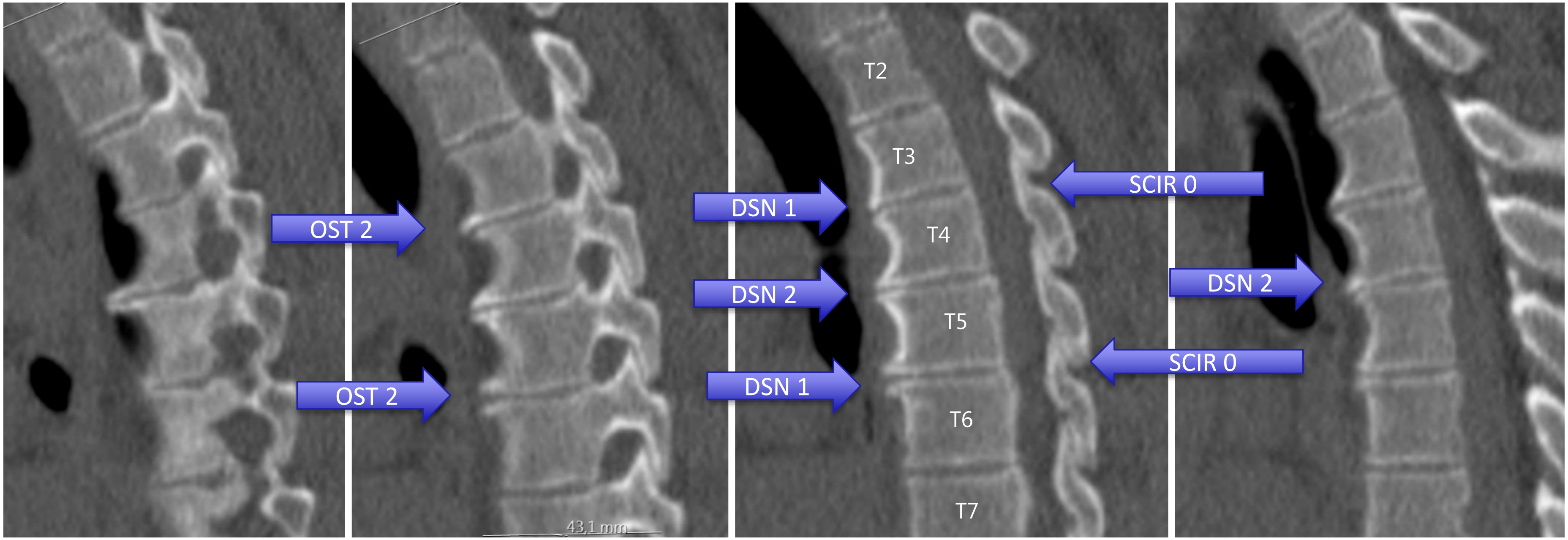

- Because of static viewing, only upper thoracic (T1-T7) is shown
- Worst Level T4-T5: Based on DSN 2
- Second worst T3-T4 or T5-T6: Based on DSN 1, SCIR 0 and OST 2
  - T5-T6 chosen as second worst, based on qualitative assessment

Mixed example 4 (DSN SCIR OST)

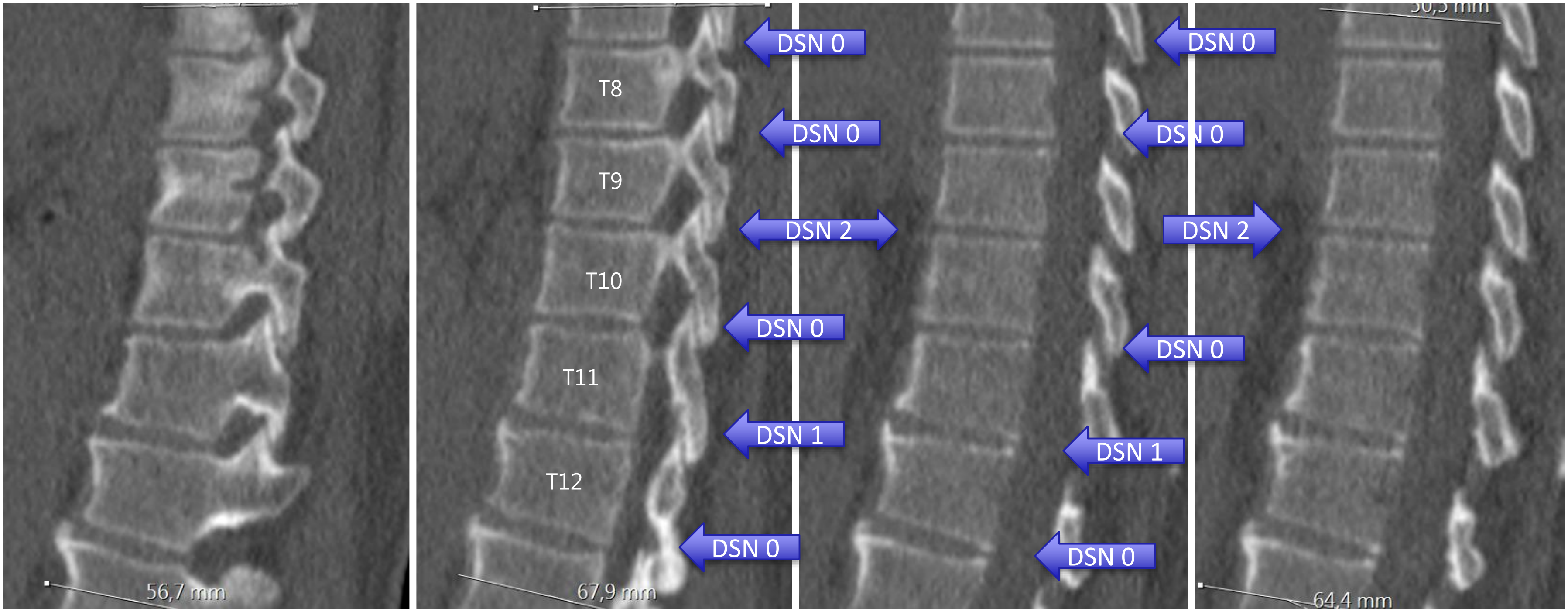

- Because of static viewing, only low thoracic (T7-L1) is shown
- Worst Level T9-10: Based on DSN 2
- Second worst T11-12: Based on DSN 1

Mixed example 4 (DSN SCIR OST)

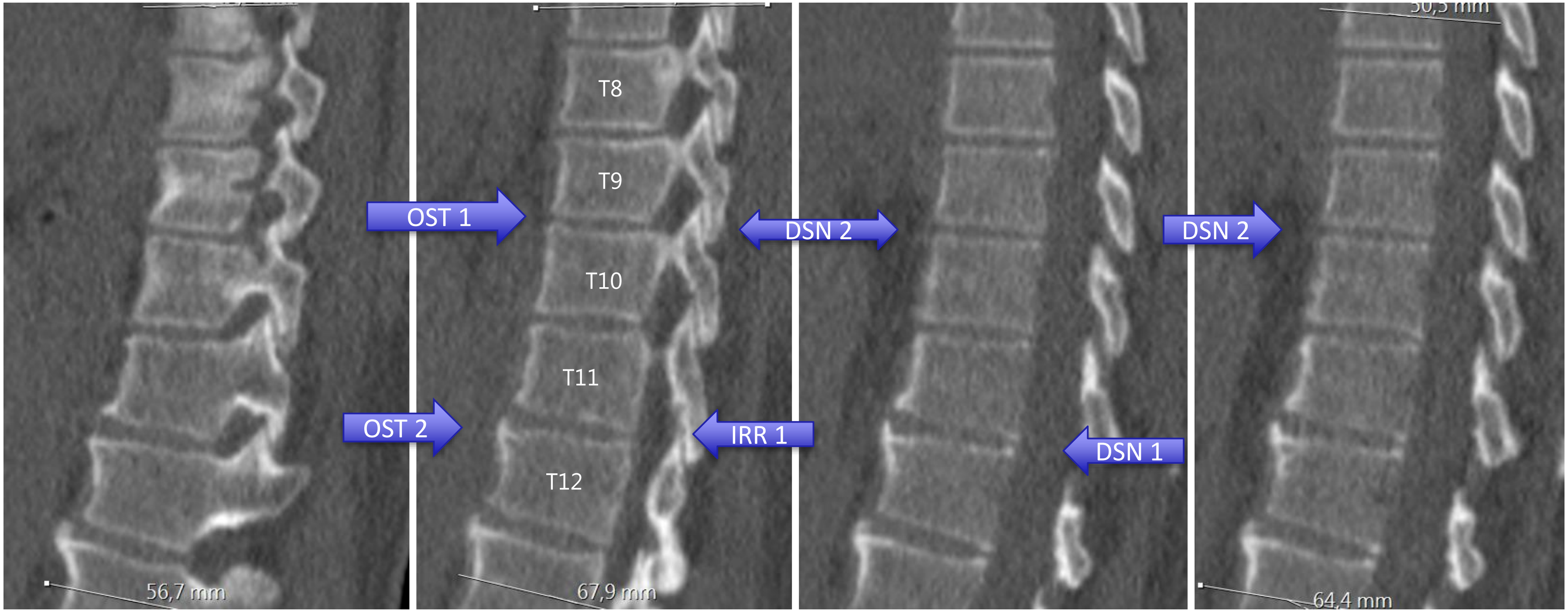

- Because of static viewing, only low thoracic (T7-L1) is shown
- Worst Level T9-10 (DSN 2, SCIR 0, OST 1)
- Second worst T11-12 (DSN 1, SCIR0, OST 2)

Intervertebral discs (IVD) total score

A separate grade is calculated for each level

- DSN score x 2 0-6
- Osteophyte score 0-3
- Sclerosis and/or Irregularity 0-1 +
- Total 0-10

- Grade 0 0-2
- Grade 1 3-5
- Grade 2 6-8
- Grade 3 9-10

Facet joint OA

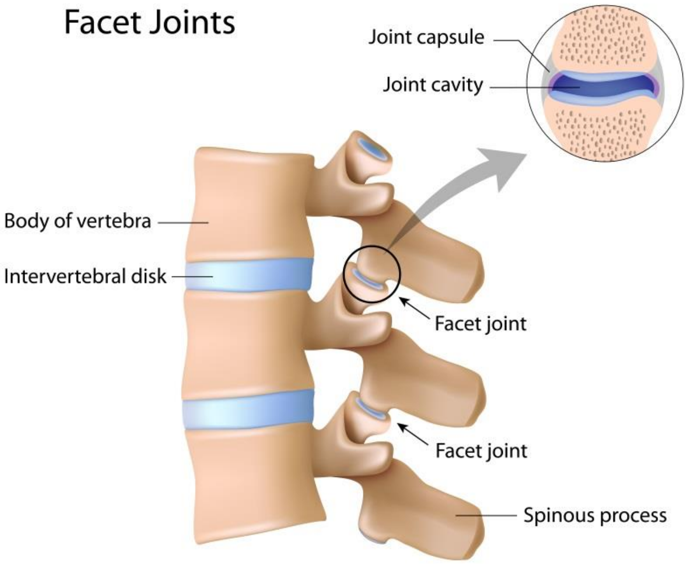

	Facet joints
Predominant view	sagittal
Settings	Multiplanar view, minimal slice thickness, bone window
Sections	Cervical (C2-C3 to C7-T1)<br>Thoracic (T1-T2 to T12-L1)<br>Lumbar (L1-L2 to L5-S1)
Grading	Grade most affected and second most effected level per section, only grade most affected side
**Facet**	**Definition**
0	Normal facet JSW (2-4mm)
1	Narrowing of facet JSW (<2mm) and small osteophytes and/or mild hypertrophy of the articular process
2	Narrowing of facet JSW (<2mm) and moderate osteophytes and/or moderate hypertrophy of the articular process and/or mild subarticular bone erosions
3	Narrowing of facet JSW (<2mm) and large osteophytes and/or severe hypertrophy of the articular process and/or severe subarticular bone erosions and/or subchondral cysts

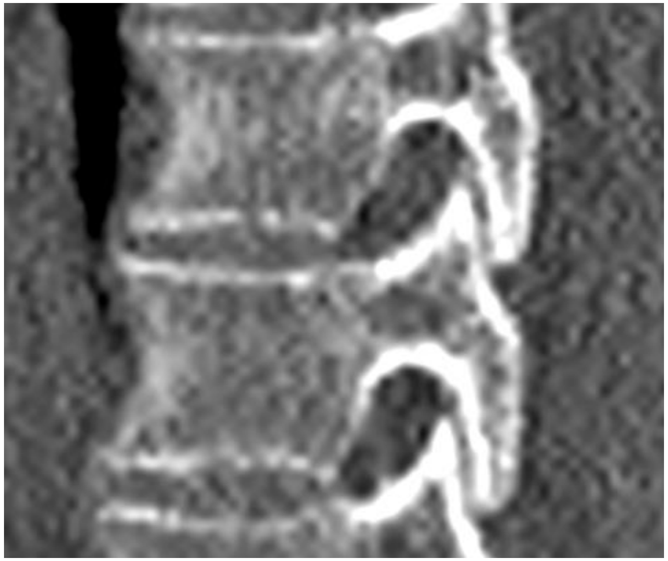

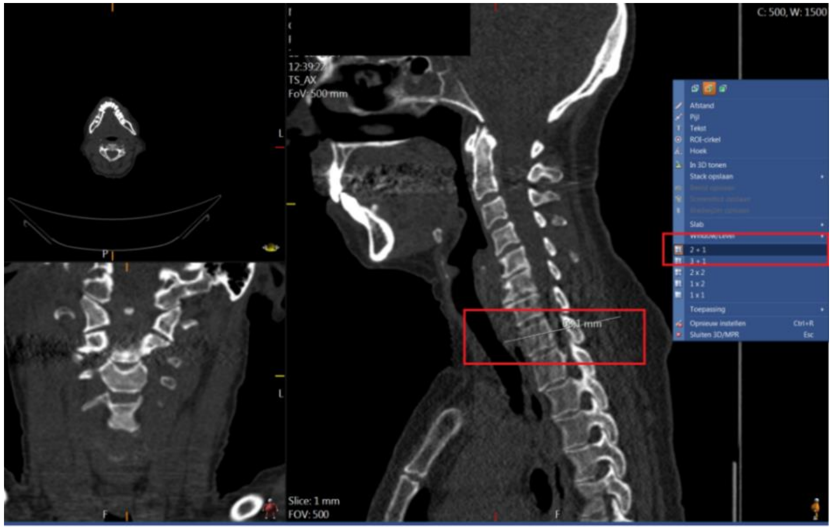

Illustration from Shutterstock

Facet cervical 0

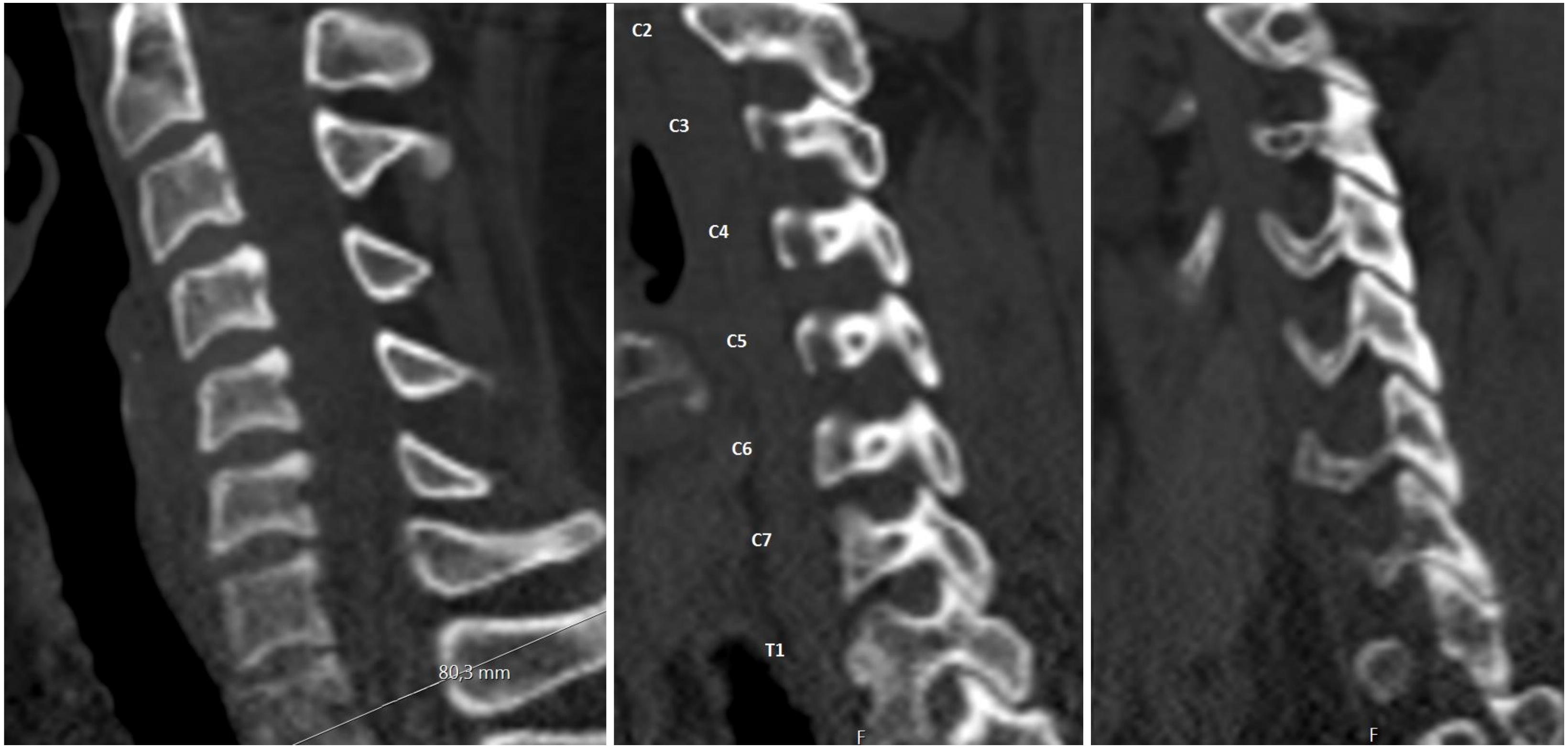

Facet	
**0**	**Normal facet JSW (2-4mm)**
1	Narrowing of facet JSW (<2mm) and small osteophytes and/or mild hypertrophy of the articular process
2	Narrowing of facet JSW (<2mm) and moderate osteophytes and/or moderate hypertrophy of the articular process and/or mild subarticular bone erosions
3	Narrowing of facet JSW (<2mm) and large osteophytes and/or severe hypertrophy of the articular process and/or severe subarticular bone erosions and/or subchondral cysts

Facet cervical 1

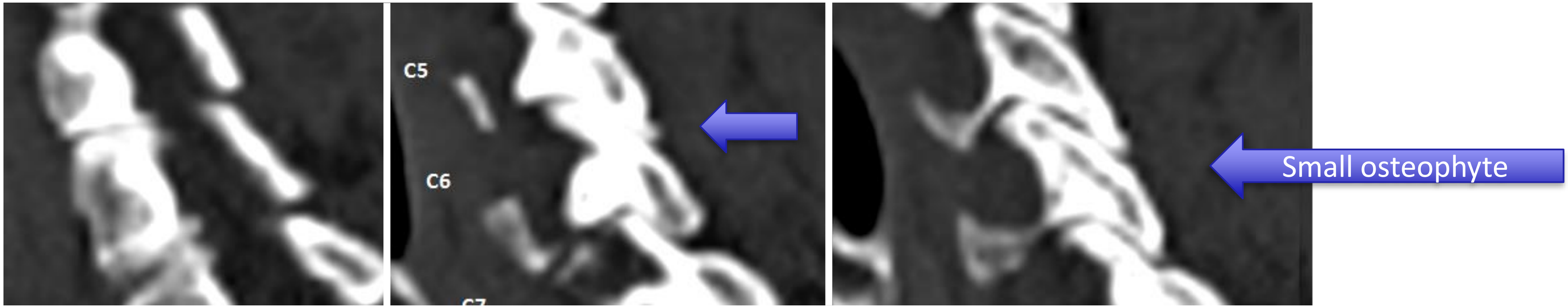

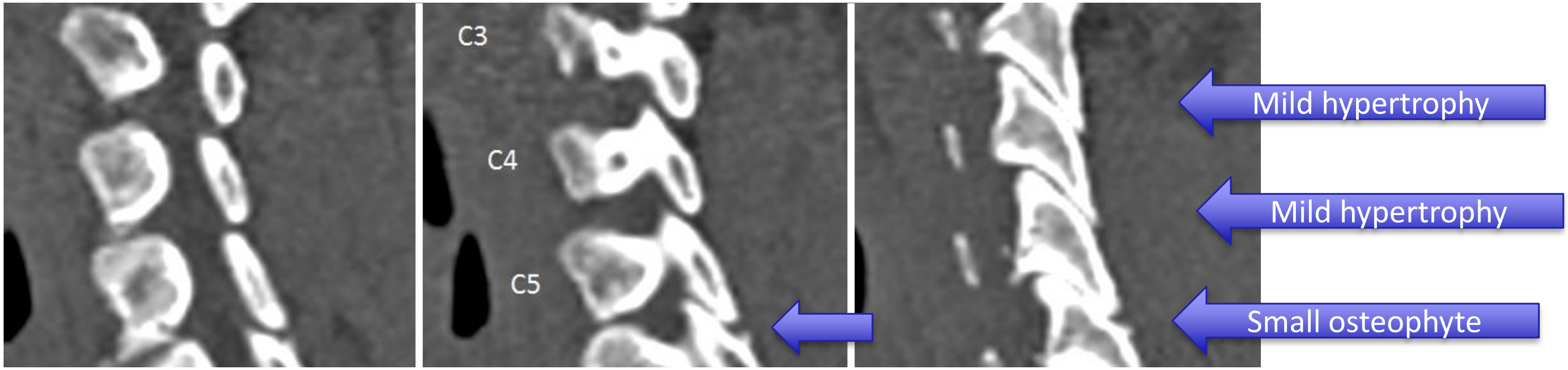

Facet	
0	Normal facet JSW (2-4mm)
**1**	**Narrowing of facet JSW (<2mm) <u>and</u> small osteophytes and/or mild hypertrophy of the articular process**
2	Narrowing of facet JSW (<2mm) and moderate osteophytes and/or moderate hypertrophy of the articular process and/or mild subarticular bone erosions
3	Narrowing of facet JSW (<2mm) and large osteophytes and/or severe hypertrophy of the articular process and/or severe subarticular bone erosions and/or subchondral cysts

Facet cervical 2

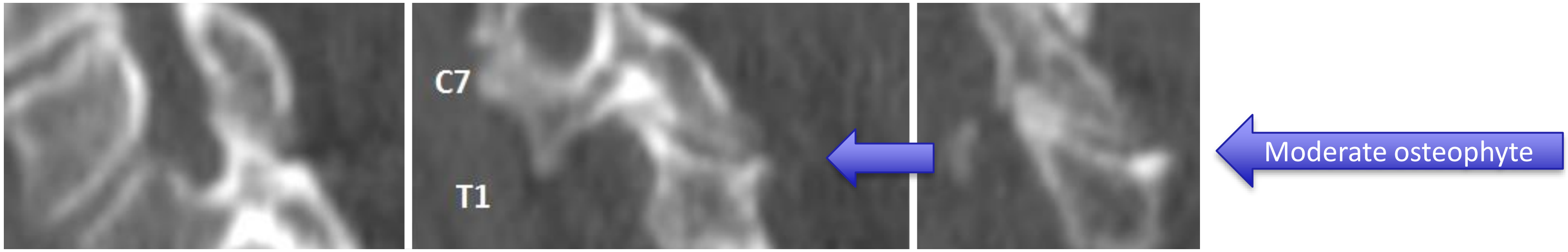

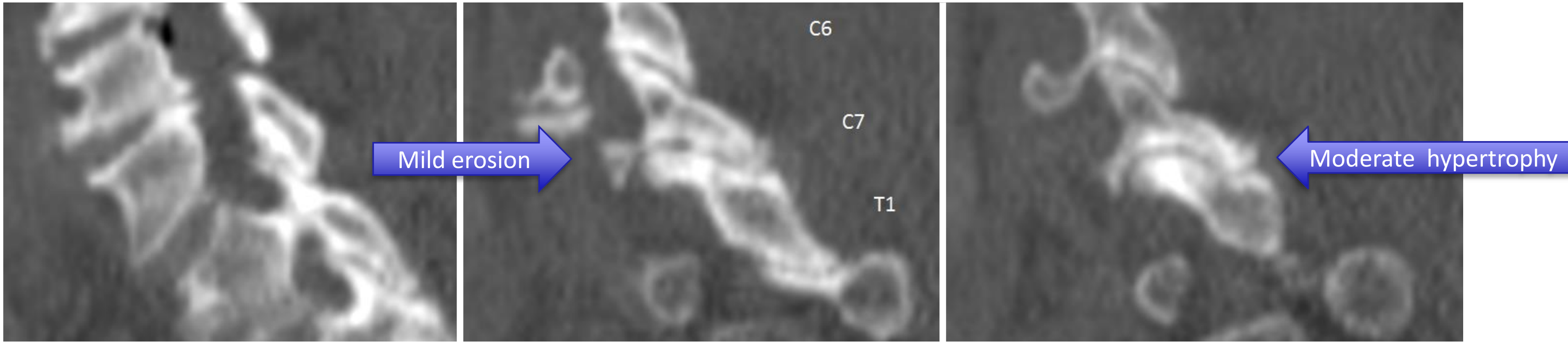

Facet	
0	Normal facet JSW (2-4mm)
1	Narrowing of facet JSW (<2mm) and small osteophytes and/or mild hypertrophy of the articular process
**2**	**Narrowing of facet JSW (<2mm) <u>and</u> moderate osteophytes and/or moderate hypertrophy of the articular process and/or mild subarticular bone erosions**
3	Narrowing of facet JSW (<2mm) and large osteophytes and/or severe hypertrophy of the articular process and/or severe subarticular bone erosions and/or subchondral cysts

Facet cervical 3

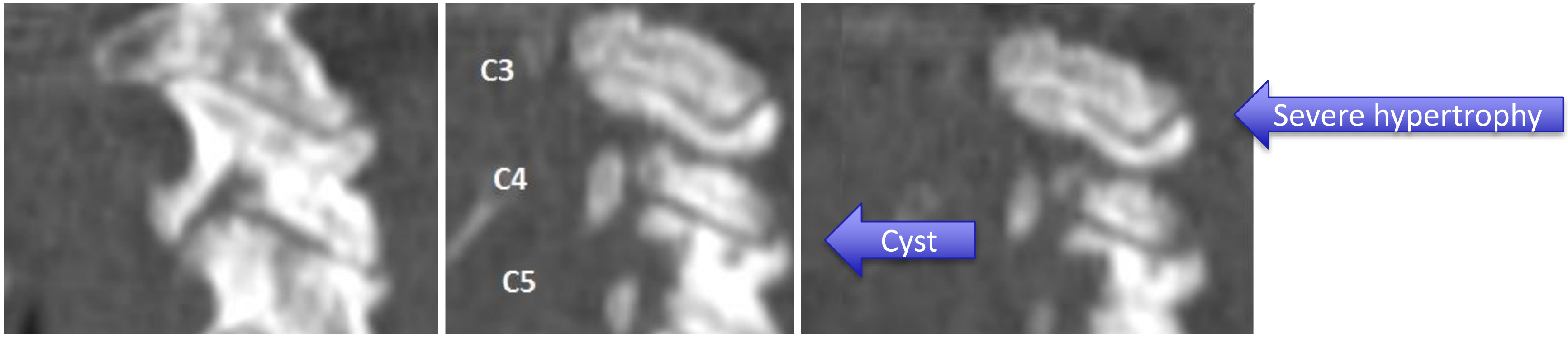

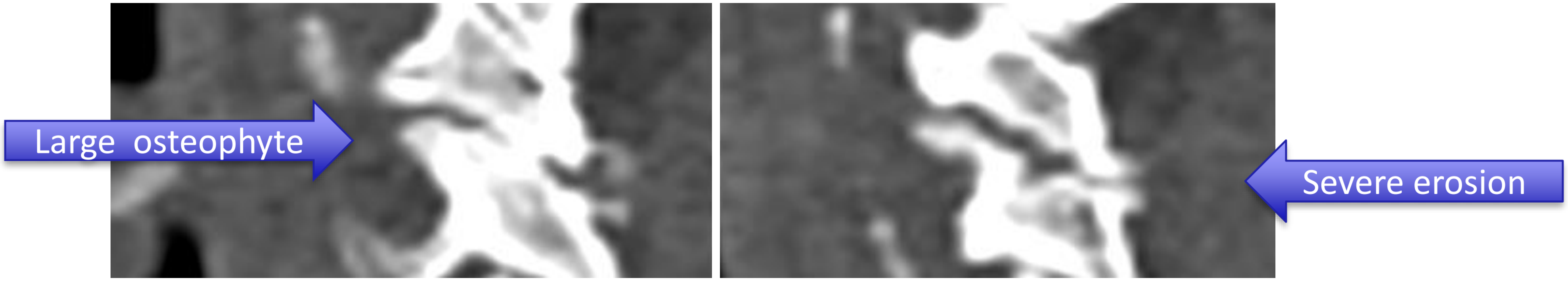

Facet	
0	Normal facet JSW (2-4mm)
1	Narrowing of facet JSW (<2mm) and small osteophytes and/or mild hypertrophy of the articular process
2	Narrowing of facet JSW (<2mm) and moderate osteophytes and/or moderate hypertrophy of the articular process and/or mild subarticular bone erosions
**3**	**Narrowing of facet JSW (<2mm) and large osteophytes and/or severe hypertrophy of the articular process and/or severe subarticular bone erosions and/or subchondral cysts**

Facet upper thoracic 0

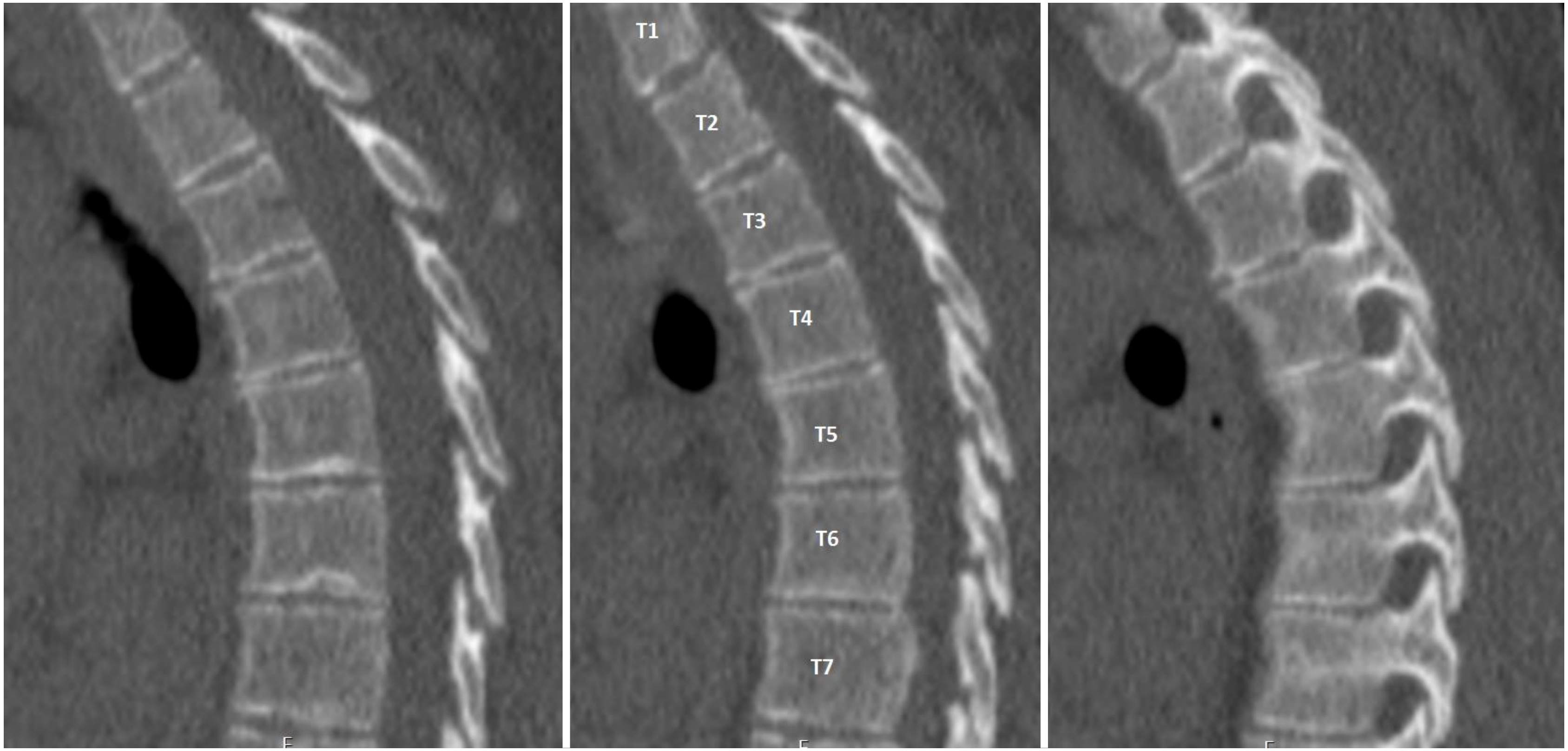

Facet	
0	**Normal facet JSW (2-4mm)**
1	Narrowing of facet JSW (<2mm) and small osteophytes and/or mild hypertrophy of the articular process
2	Narrowing of facet JSW (<2mm) and moderate osteophytes and/or moderate hypertrophy of the articular process and/or mild subarticular bone erosions
3	Narrowing of facet JSW (<2mm) and large osteophytes and/or severe hypertrophy of the articular process and/or severe subarticular bone erosions and/or subchondral cysts

Facet lower thoracic 0

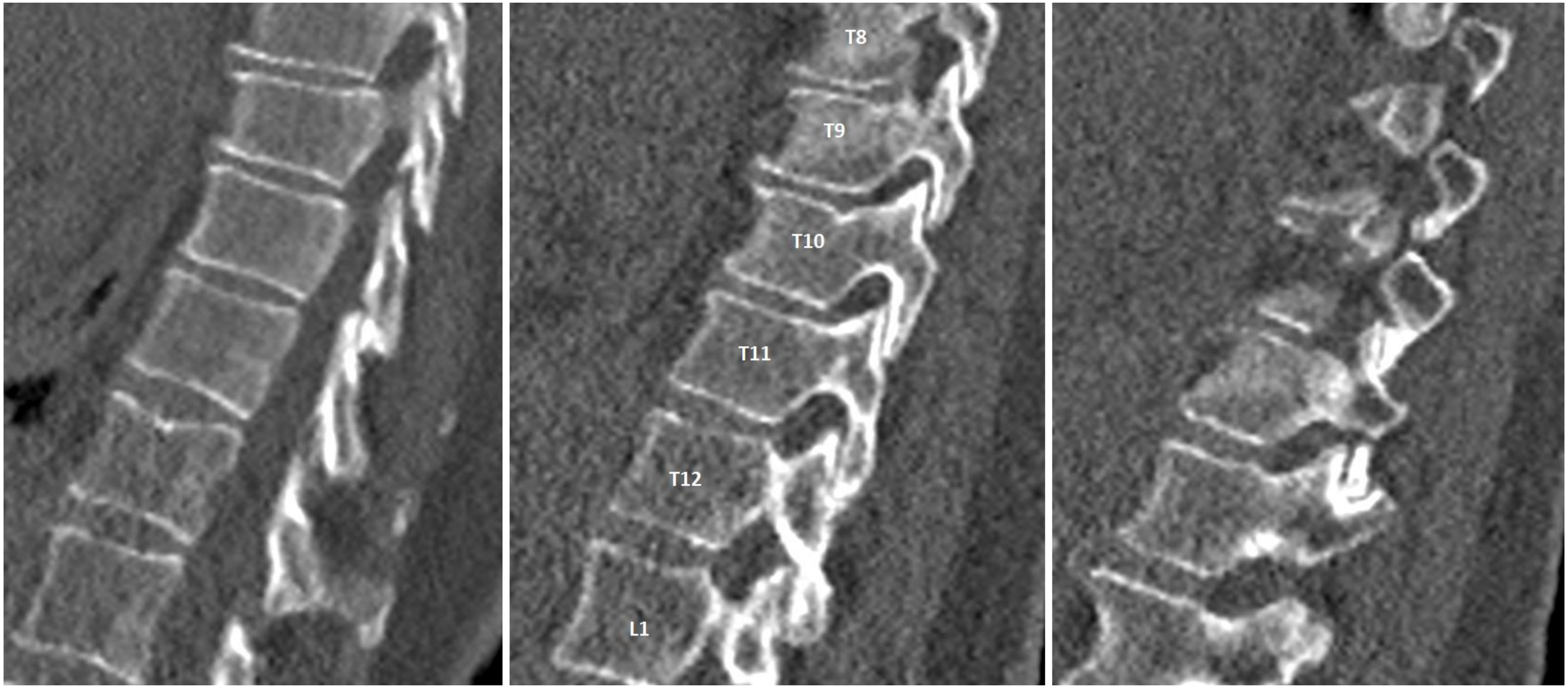

Facet	
**0**	**Normal facet JSW (2-4mm)**
1	Narrowing of facet JSW (<2mm) and small osteophytes and/or mild hypertrophy of the articular process
2	Narrowing of facet JSW (<2mm) and moderate osteophytes and/or moderate hypertrophy of the articular process and/or mild subarticular bone erosions
3	Narrowing of facet JSW (<2mm) and large osteophytes and/or severe hypertrophy of the articular process and/or severe subarticular bone erosions and/or subchondral cysts

Facet thoracic 1

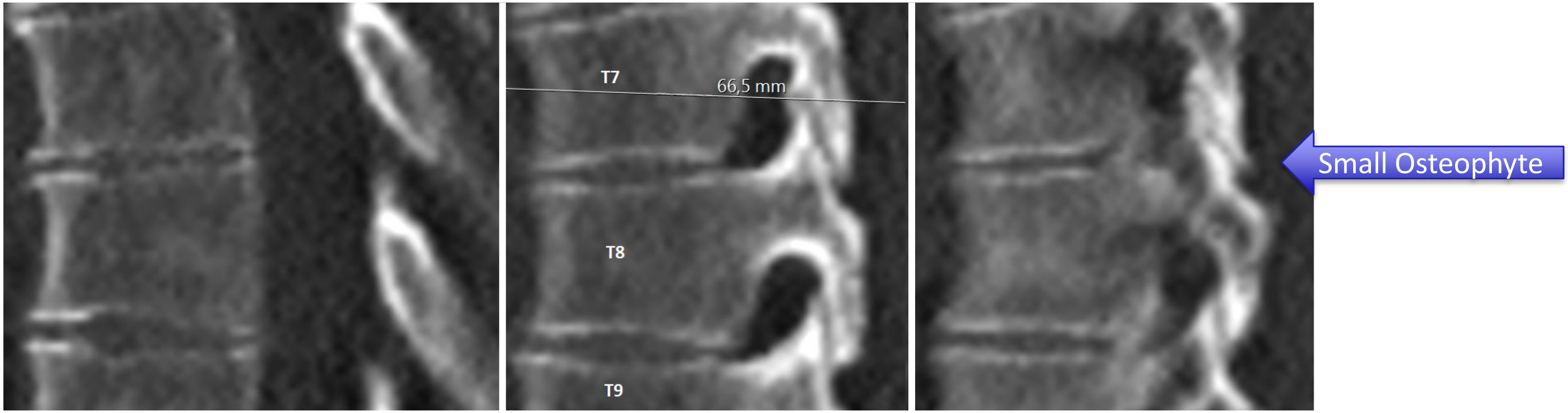

Facet	
0	Normal facet JSW (2-4mm)
**1**	**Narrowing of facet JSW (<2mm) <u>and</u> small osteophytes and/or mild hypertrophy of the articular process**
2	Narrowing of facet JSW (<2mm) and moderate osteophytes and/or moderate hypertrophy of the articular process and/or mild subarticular bone erosions
3	Narrowing of facet JSW (<2mm) and large osteophytes and/or severe hypertrophy of the articular process and/or severe subarticular bone erosions and/or subchondral cysts

Facet thoracic 2

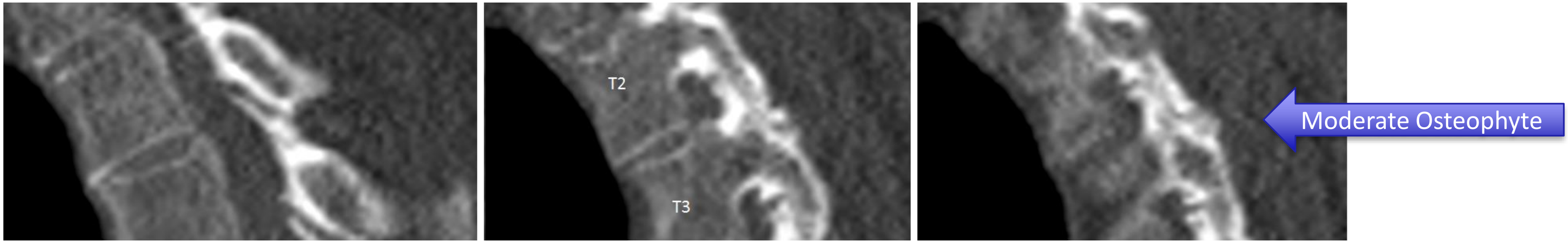

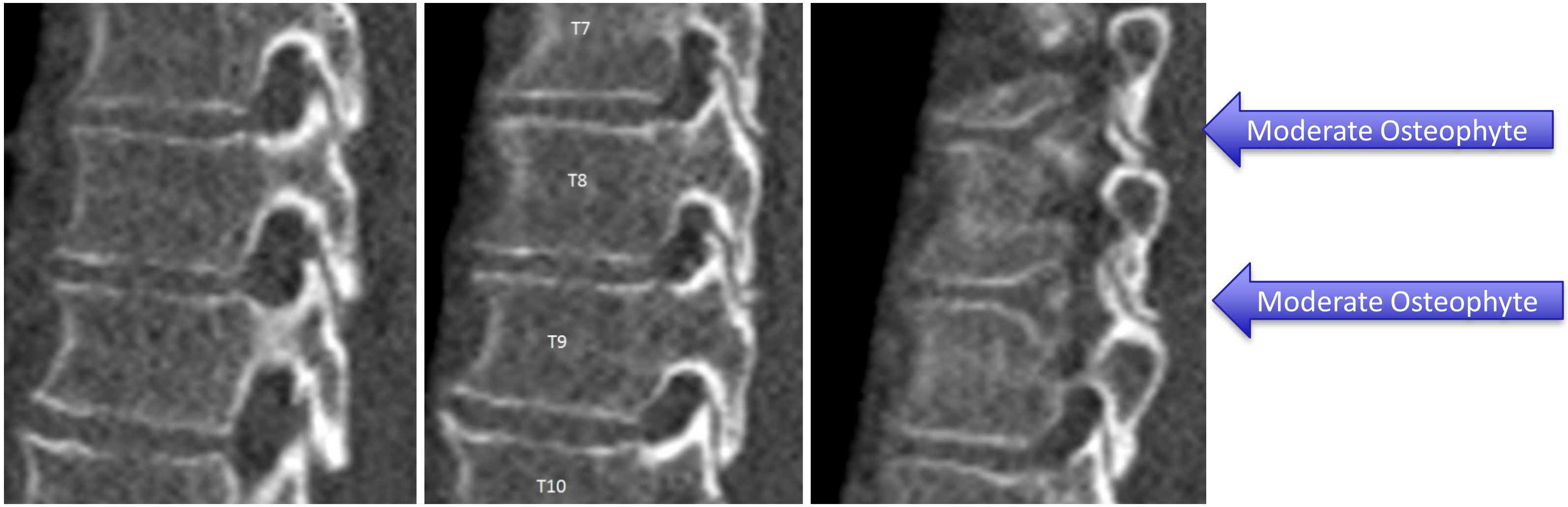

Facet	
0	Normal facet JSW (2-4mm)
1	Narrowing of facet JSW (<2mm) and small osteophytes and/or mild hypertrophy of the articular process
**2**	**Narrowing of facet JSW (<2mm) <u>and</u> moderate osteophytes and/or moderate hypertrophy of the articular process and/or mild subarticular bone erosions**
3	Narrowing of facet JSW (<2mm) and large osteophytes and/or severe hypertrophy of the articular process and/or severe subarticular bone erosions and/or subchondral cysts

Facet thoracic 2 – 1 – 0

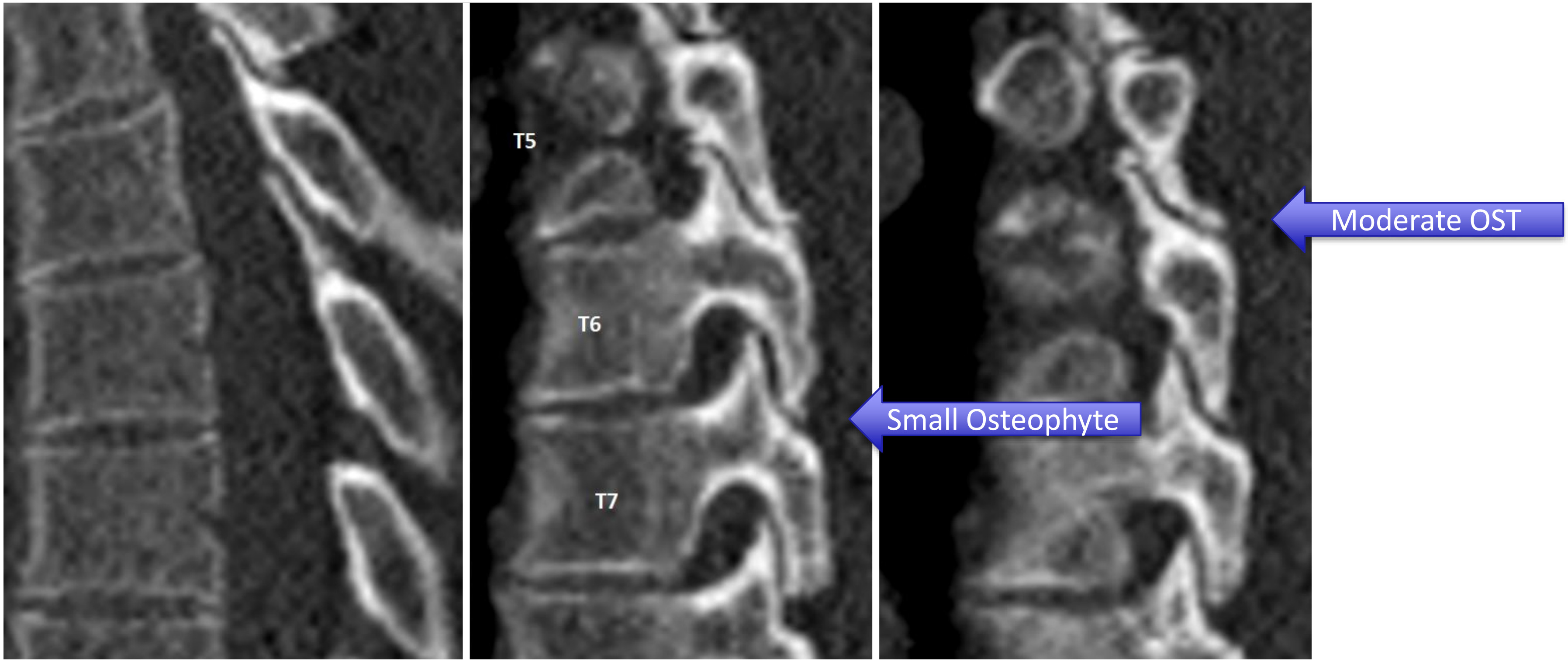

Facet	
0	Normal facet JSW (2-4mm)
1	Narrowing of facet JSW (<2mm) and small osteophytes and/or mild hypertrophy of the articular process
2	Narrowing of facet JSW (<2mm) and moderate osteophytes and/or moderate hypertrophy of the articular process and/or mild subarticular bone erosions
3	Narrowing of facet JSW (<2mm) and large osteophytes and/or severe hypertrophy of the articular process and/or severe subarticular bone erosions and/or subchondral cysts

Facet thoracic 3

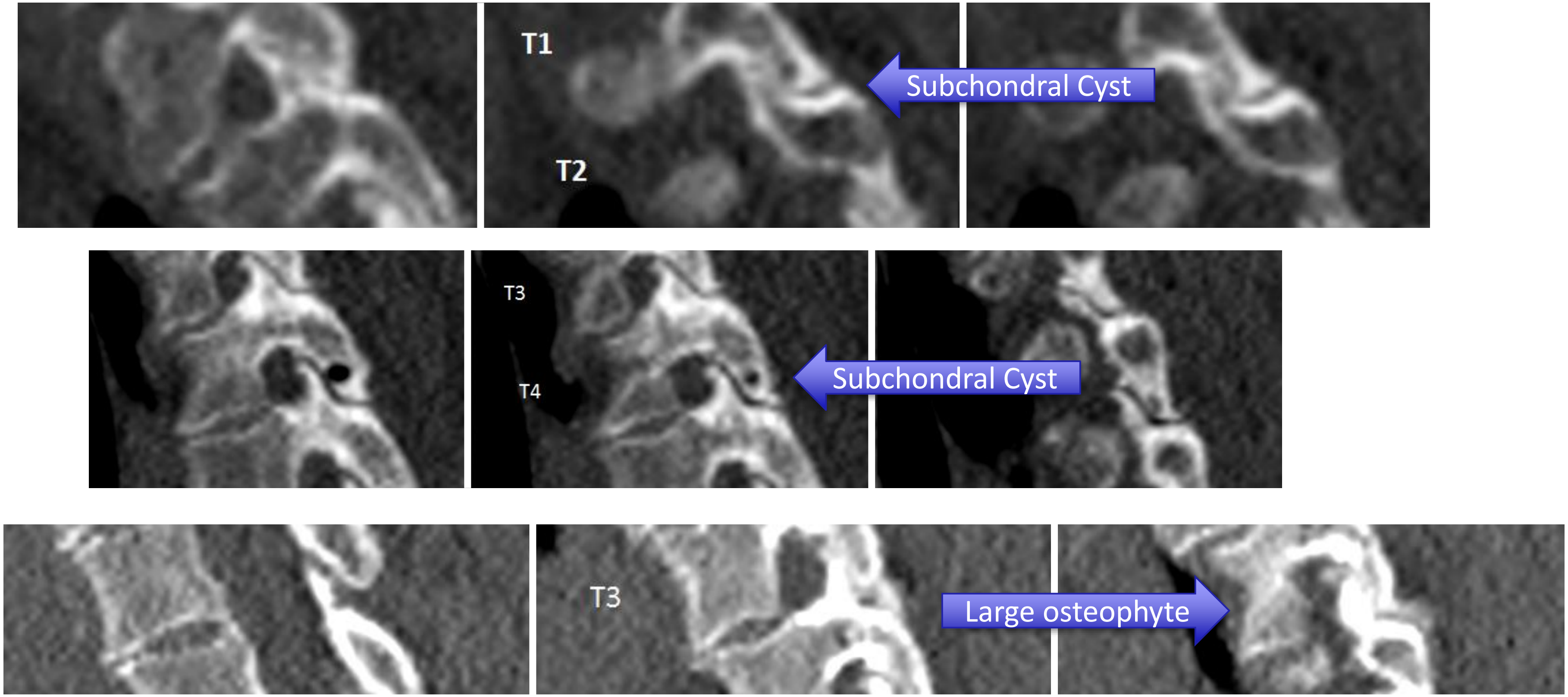

Facet	
0	Normal facet JSW (2-4mm)
1	Narrowing of facet JSW (<2mm) and small osteophytes and/or mild hypertrophy of the articular process
2	Narrowing of facet JSW (<2mm) and moderate osteophytes and/or moderate hypertrophy of the articular process and/or mild subarticular bone erosions
**3**	**Narrowing of facet JSW (<2mm) and large osteophytes and/or severe hypertrophy of the articular process and/or severe subarticular bone erosions and/or subchondral cysts**

Facet lumbar 0

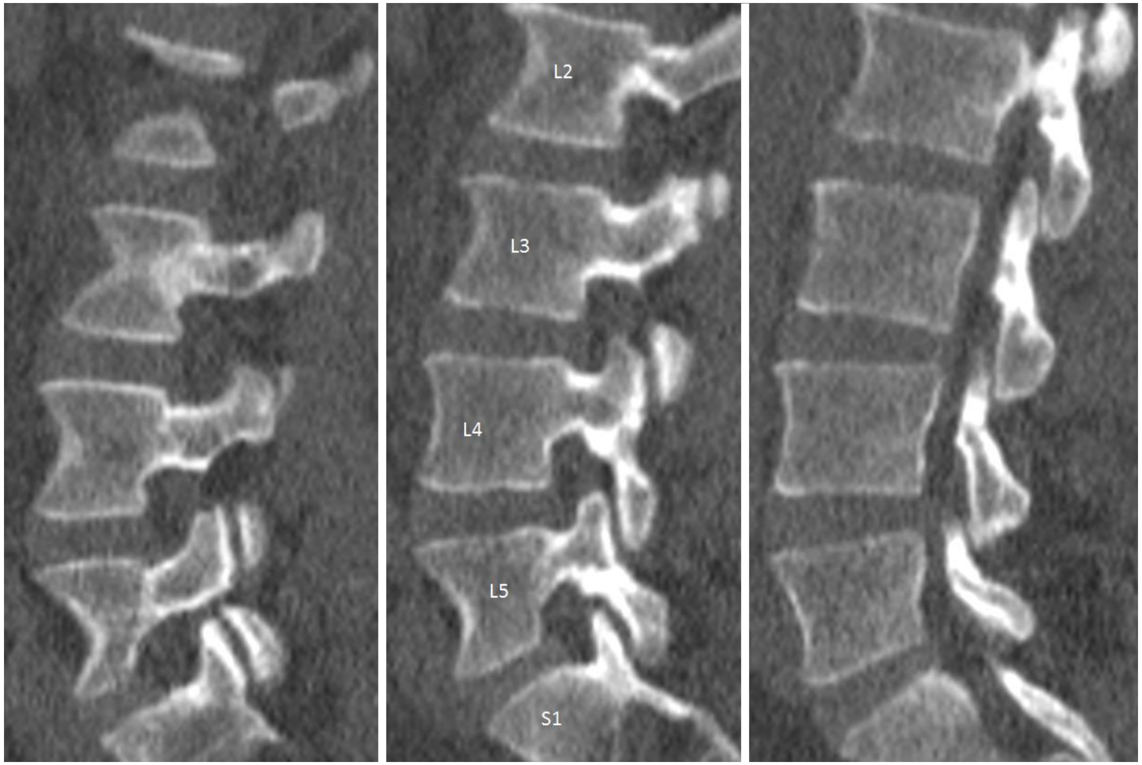

Facet	
**0**	**Normal facet JSW (2-4mm)**
1	Narrowing of facet JSW (<2mm) and small osteophytes and/or mild hypertrophy of the articular process
2	Narrowing of facet JSW (<2mm) and moderate osteophytes and/or moderate hypertrophy of the articular process and/or mild subarticular bone erosions
3	Narrowing of facet JSW (<2mm) and large osteophytes and/or severe hypertrophy of the articular process and/or severe subarticular bone erosions and/or subchondral cysts

Facet lumbar 0

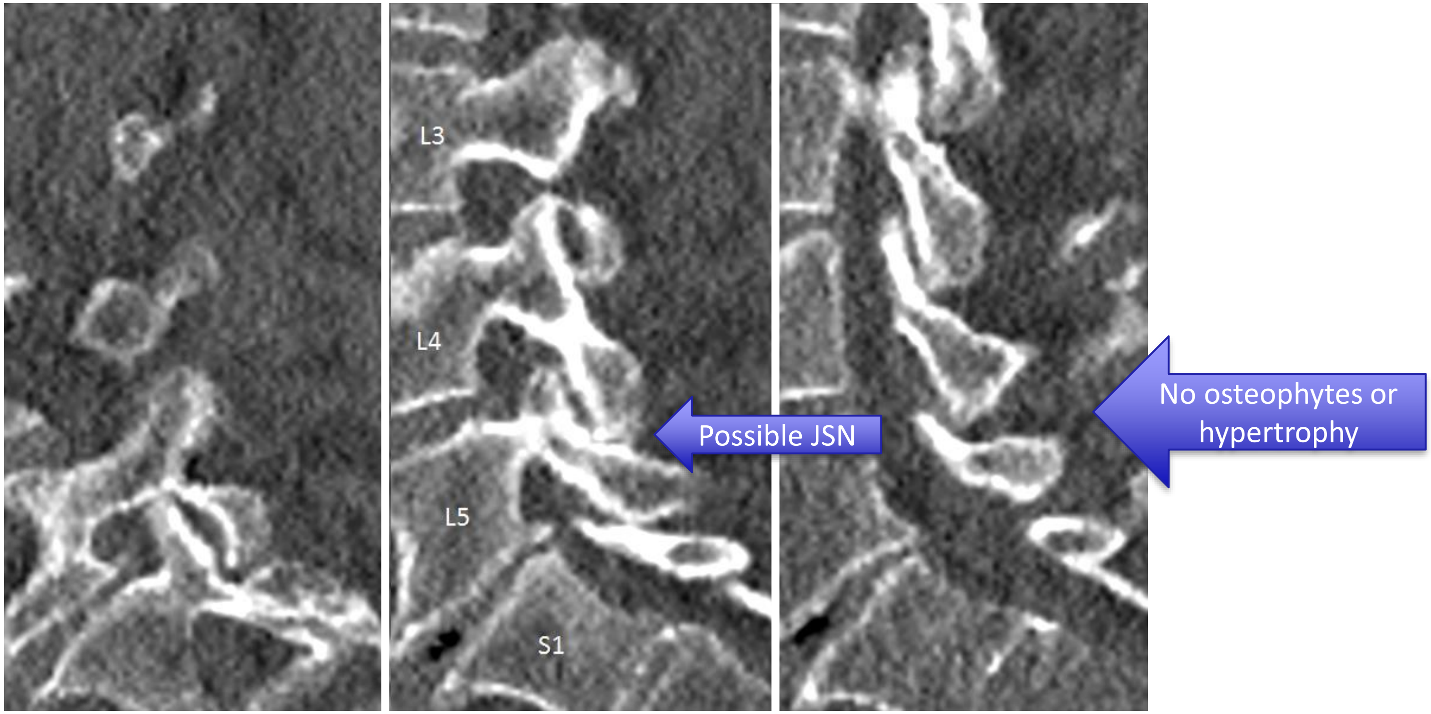

Facet	
**0**	**Normal facet JSW (2-4mm)**
1	Narrowing of facet JSW (<2mm) and small osteophytes and/or mild hypertrophy of the articular process
2	Narrowing of facet JSW (<2mm) and moderate osteophytes and/or moderate hypertrophy of the articular process and/or mild subarticular bone erosions
3	Narrowing of facet JSW (<2mm) and large osteophytes and/or severe hypertrophy of the articular process and/or severe subarticular bone erosions and/or subchondral cysts

Facet lumbar 1

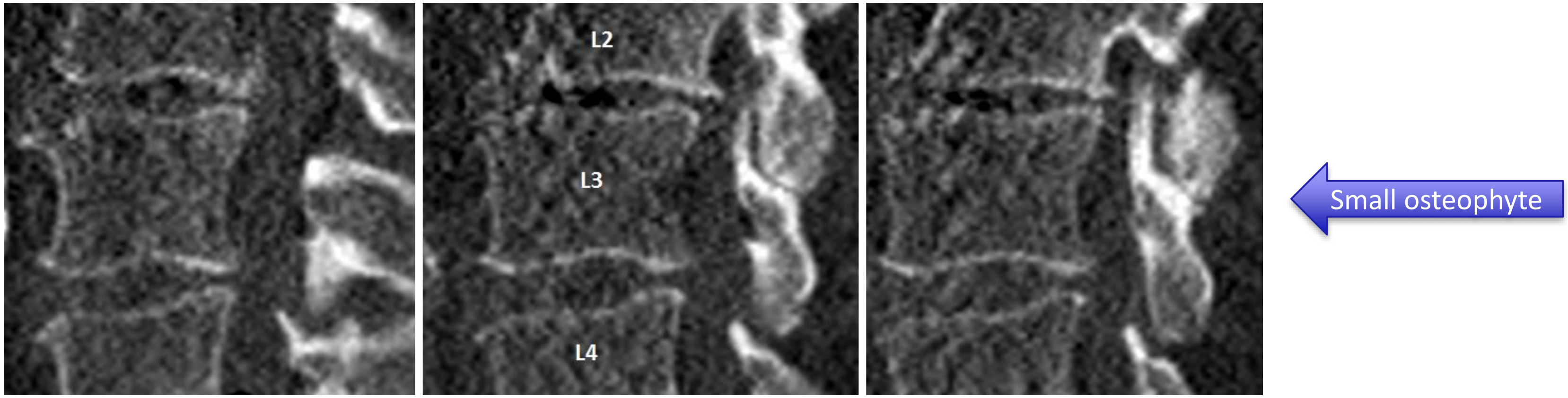

Facet	
0	Normal facet JSW (2-4mm)
**1**	**Narrowing of facet JSW (<2mm) and small osteophytes and/or mild hypertrophy of the articular process**
2	Narrowing of facet JSW (<2mm) and moderate osteophytes and/or moderate hypertrophy of the articular process and/or mild subarticular bone erosions
3	Narrowing of facet JSW (<2mm) and large osteophytes and/or severe hypertrophy of the articular process and/or severe subarticular bone erosions and/or subchondral cysts

Facet lumbar 1

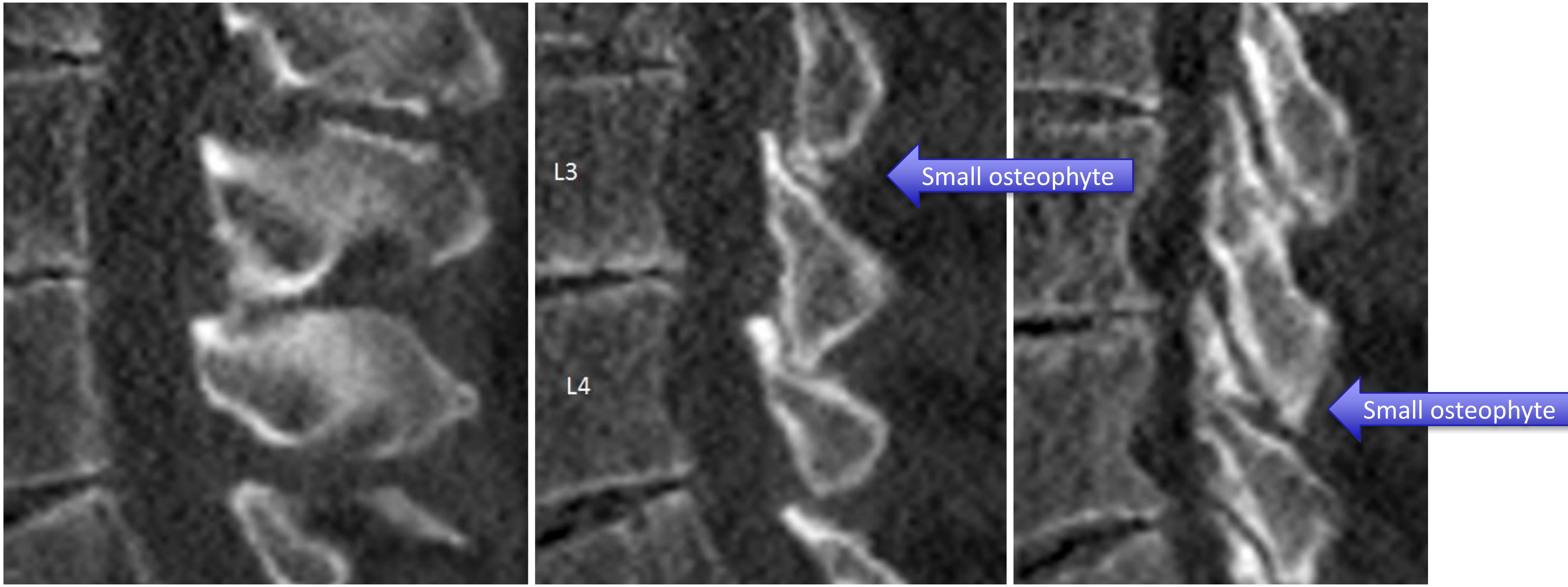

Facet	
0	Normal facet JSW (2-4mm)
**1**	**Narrowing of facet JSW (<2mm) and small osteophytes and/or mild hypertrophy of the articular process**
2	Narrowing of facet JSW (<2mm) and moderate osteophytes and/or moderate hypertrophy of the articular process and/or mild subarticular bone erosions
3	Narrowing of facet JSW (<2mm) and large osteophytes and/or severe hypertrophy of the articular process and/or severe subarticular bone erosions and/or subchondral cysts

Facet lumbar 2

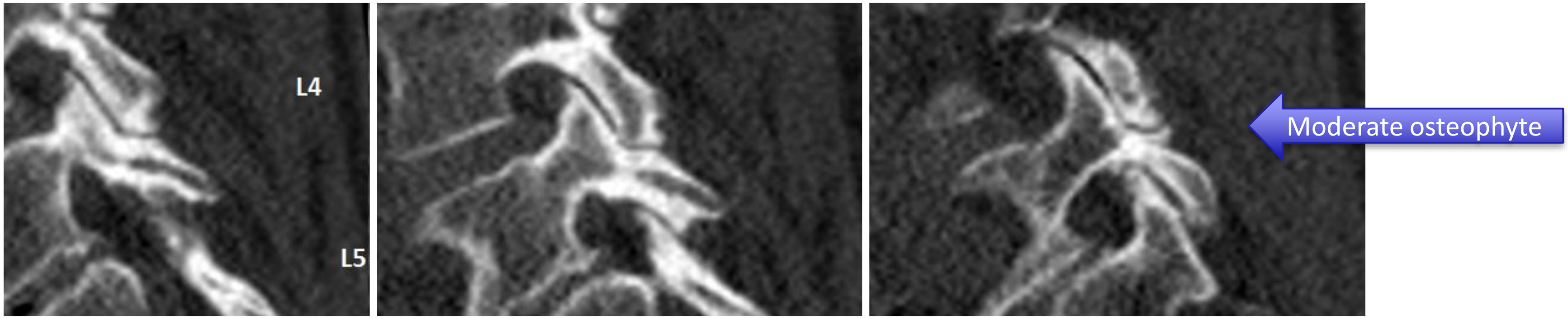

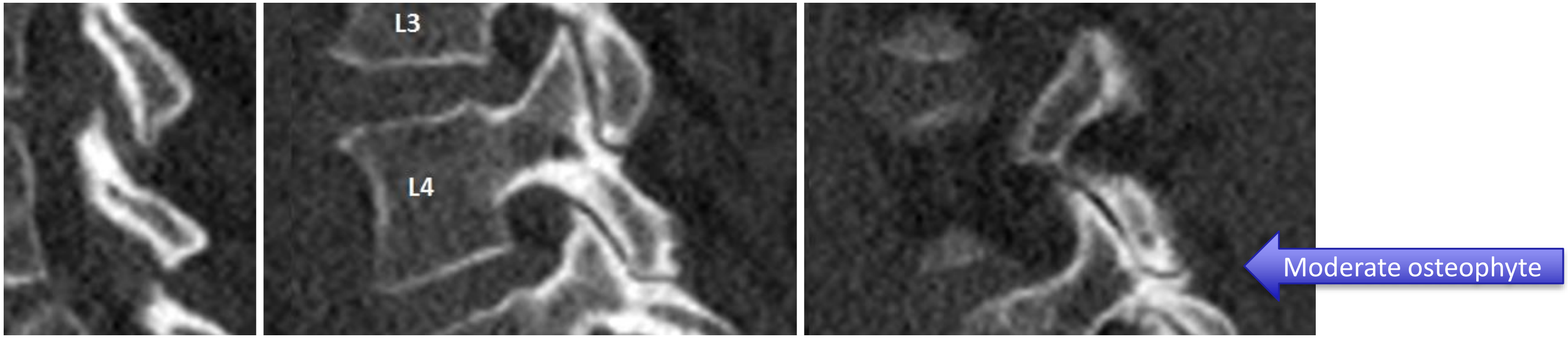

Facet	
0	Normal facet JSW (2-4mm)
1	Narrowing of facet JSW (<2mm) and small osteophytes and/or mild hypertrophy of the articular process
**2**	**Narrowing of facet JSW (<2mm) and moderate osteophytes and/or moderate hypertrophy of the articular process and/or mild subarticular bone erosions**
3	Narrowing of facet JSW (<2mm) and large osteophytes and/or severe hypertrophy of the articular process and/or severe subarticular bone erosions and/or subchondral cysts

Facet lumbar 3

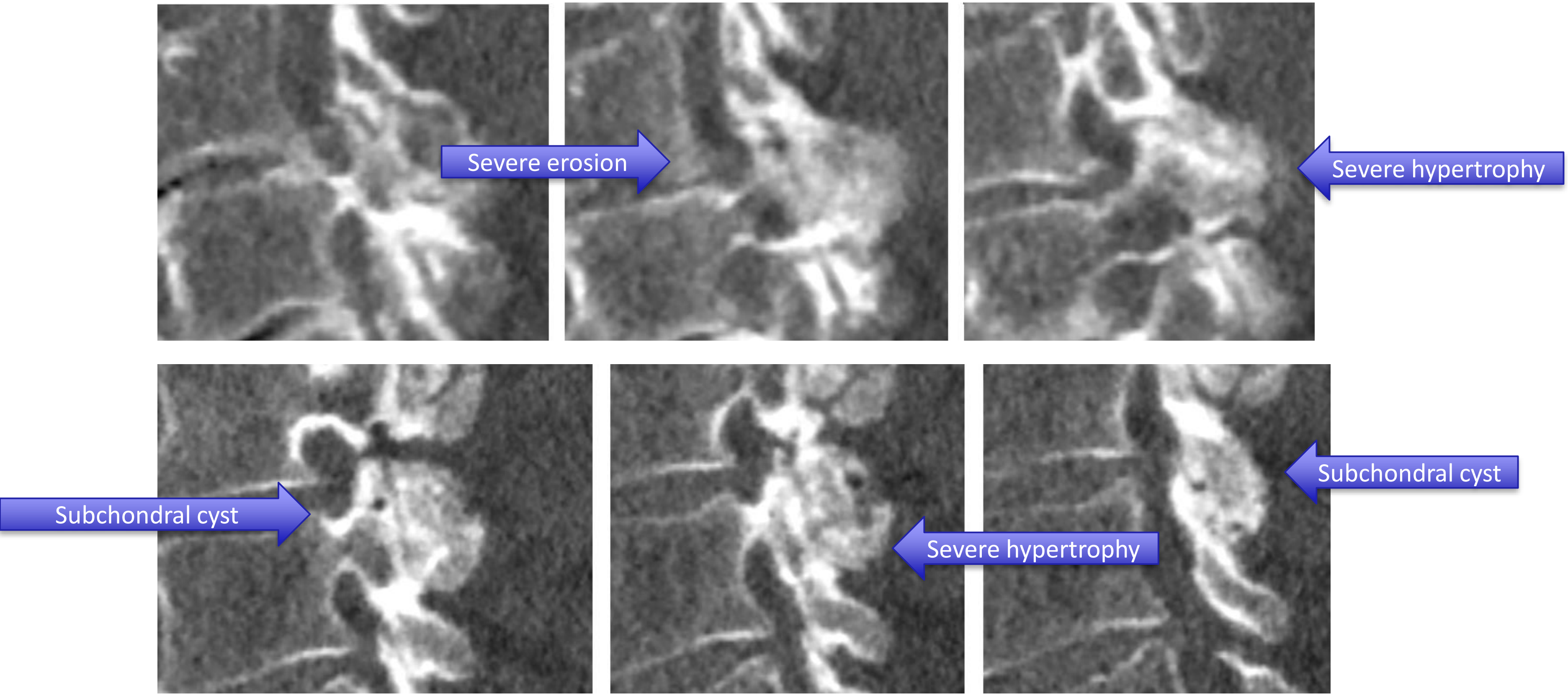

Facet	
0	Normal facet JSW (2-4mm)
1	Narrowing of facet JSW (<2mm) and small osteophytes and/or mild hypertrophy of the articular process
2	Narrowing of facet JSW (<2mm) and moderate osteophytes and/or moderate hypertrophy of the articular process and/or mild subarticular bone erosions
**3**	**Narrowing of facet JSW (<2mm) <u>and</u> large osteophytes and/or severe hypertrophy of the articular process and/or severe subarticular bone erosions and/or subchondral cysts**

Facet lumbar 3

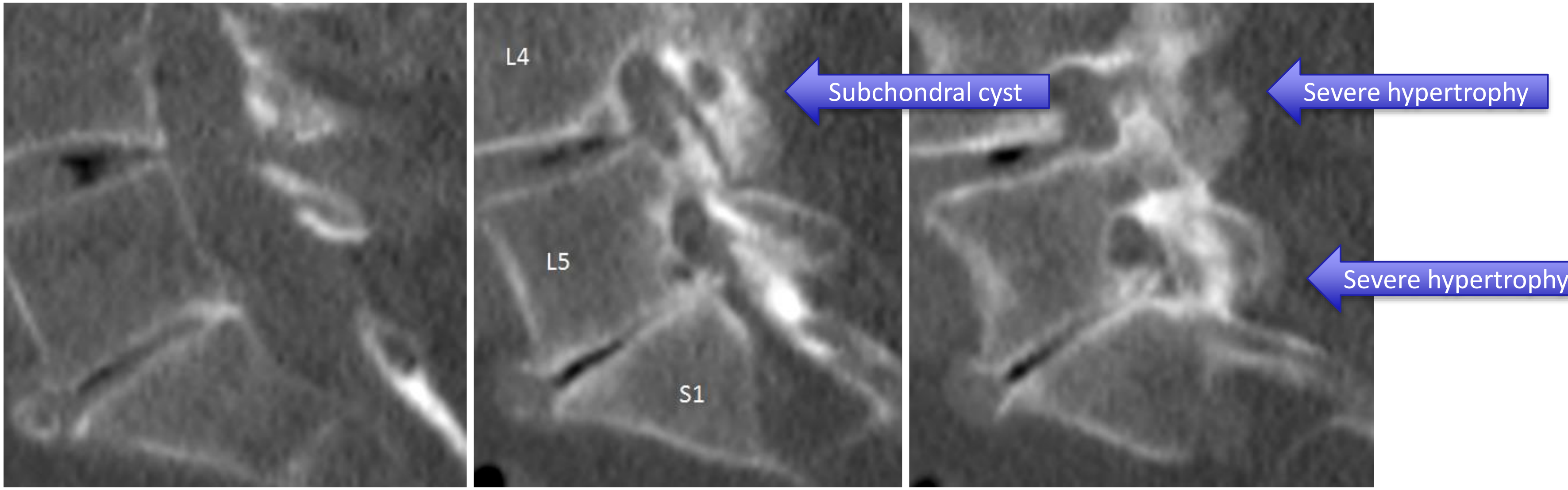

Facet	
0	Normal facet JSW (2-4mm)
1	Narrowing of facet JSW (<2mm) and small osteophytes and/or mild hypertrophy of the articular process
2	Narrowing of facet JSW (<2mm) and moderate osteophytes and/or moderate hypertrophy of the articular process and/or mild subarticular bone erosions
**3**	**Narrowing of facet JSW (<2mm) <u>and</u> large osteophytes and/or severe hypertrophy of the articular process and/or severe subarticular bone erosions and/or subchondral cysts**

Hip joint

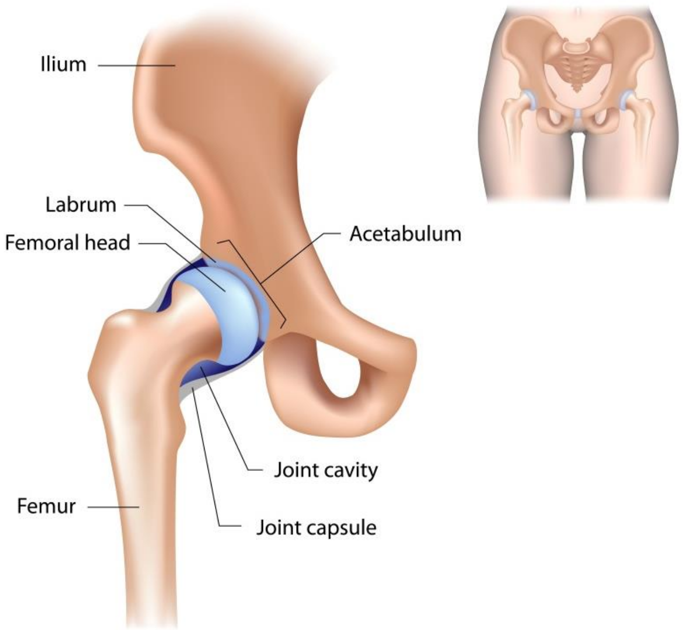

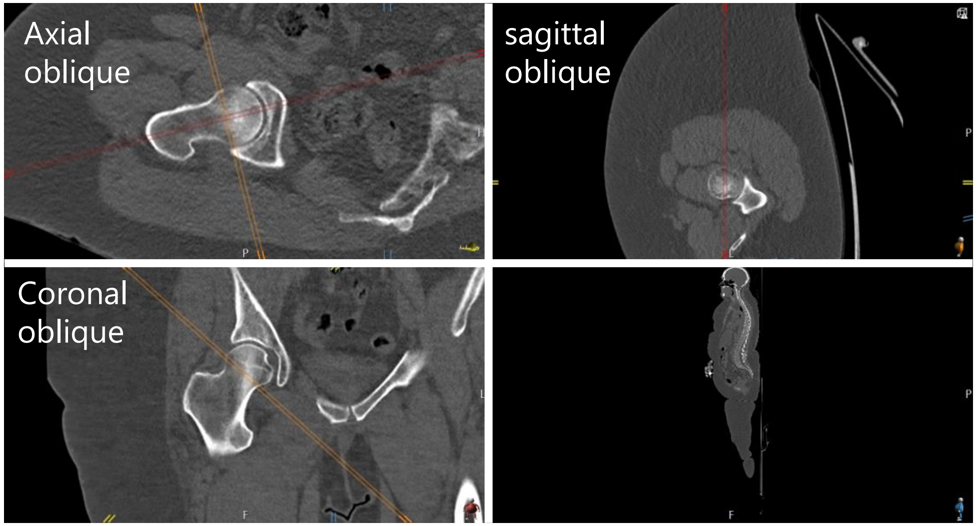

	Hip joint
Predominant view	Coronal oblique and axial oblique
Settings	Multiplanar view, minimal slice thickness, bone window
Sections	Left and Right
Grading	3 grades per section<br>Joint Space Narrowing (JSN)<br>Osteophytes (OST)<br>Cysts (CYST)
**JSN**	**Definition**
0	No JSN (<10% surface within 1.5mm)
1	Possible JSN (10-30% surface within 1.5mm)
2	Definite JSN (30-60% surface within 1.5mm)
3	Obliterated JS (>60% surface within 1.5mm)
**OST**	**Definition**
0	No osteophyte
1	Possible / small osteophyte
2	Moderate osteophyte (≤ 5 mm)
3	Large osteophyte (> 5 mm)
**CYST**	**Definition**
0	No subchondral cysts or criteria grade 1 not met
1	Single cyst, maximal diameter > 5mm or Multiple cysts, maximal diameter ≤ 5 mm in same region

Illustration from Shutterstock

Views Hip

- Start by zooming axial, coronal and sagittal slices on hip joint

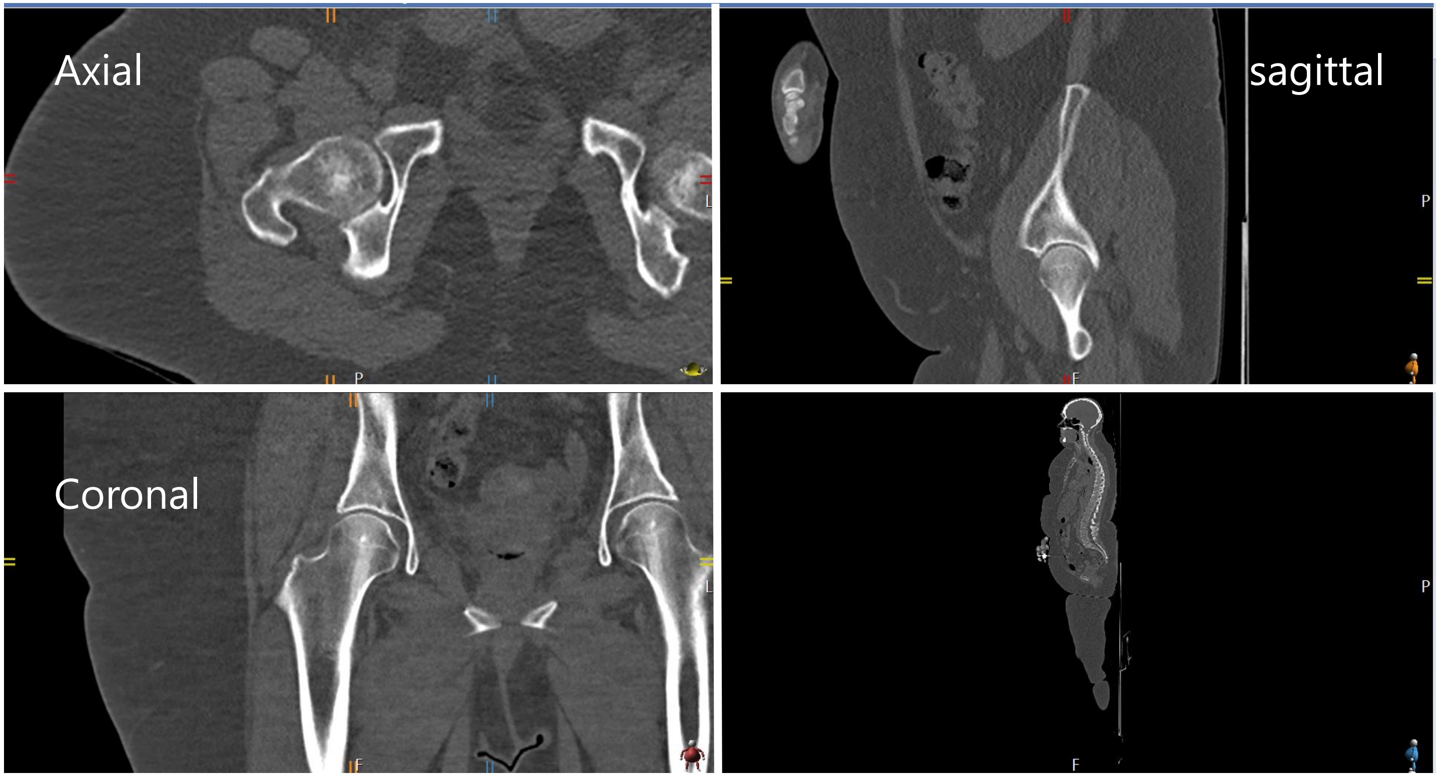

Views Hip

- Firstly, adjust the axial plane to be in line with the femoral shaft

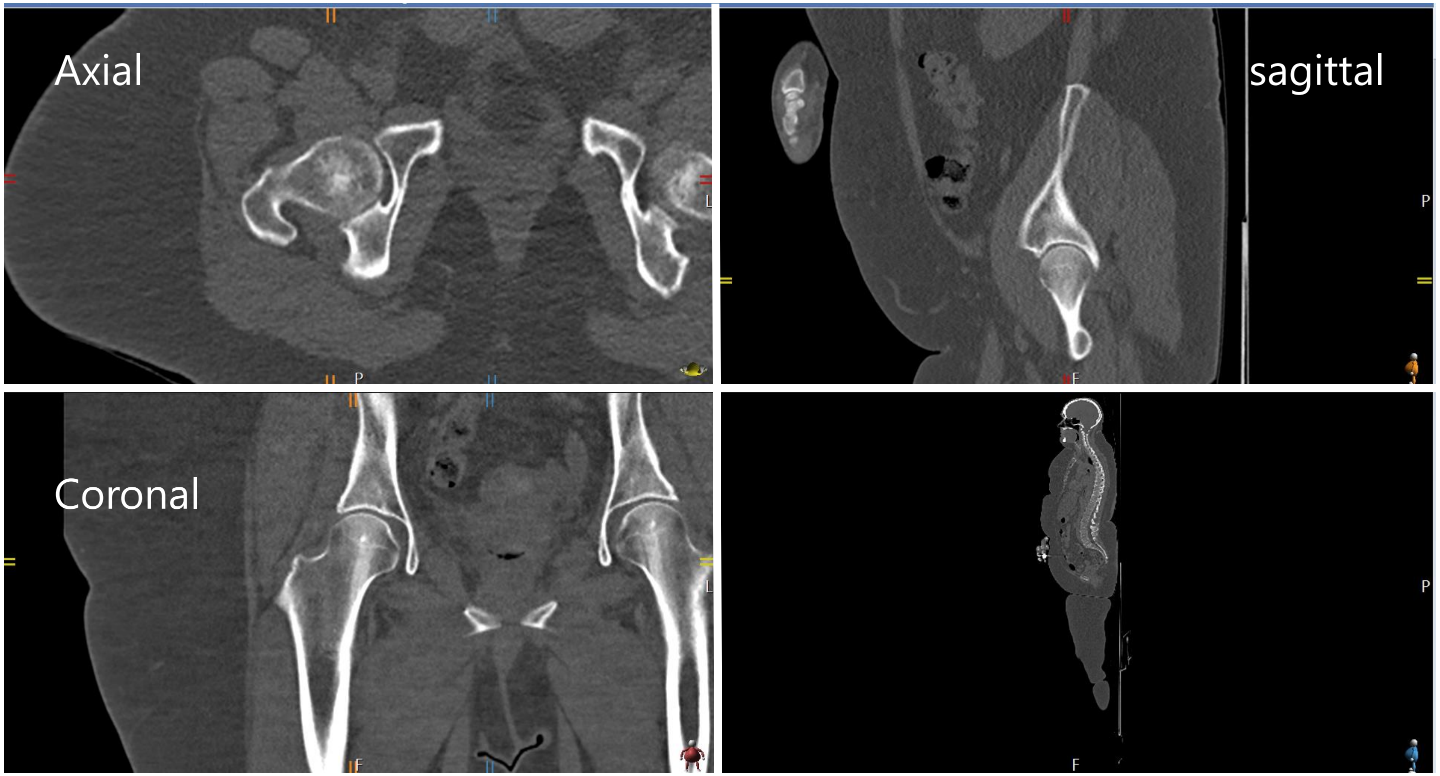

Views Hip

- Secondly, adjust the coronal and sagittal plane to be in line with the femoral shaft

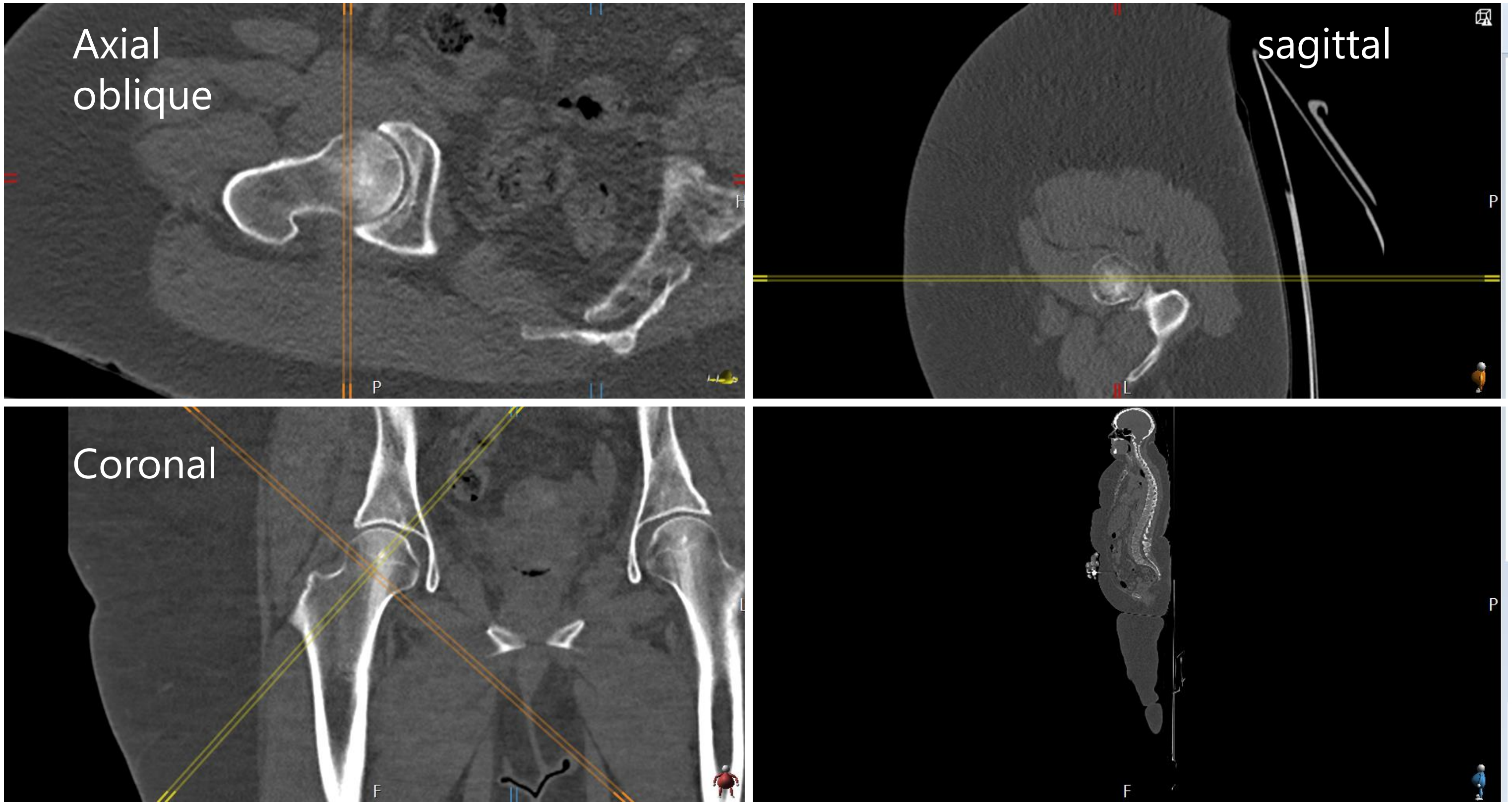

Views Hip

- These oblique views will be used for all scores of the hip (osteophytes, cysts and JSW)
  - Minimal slice thickness

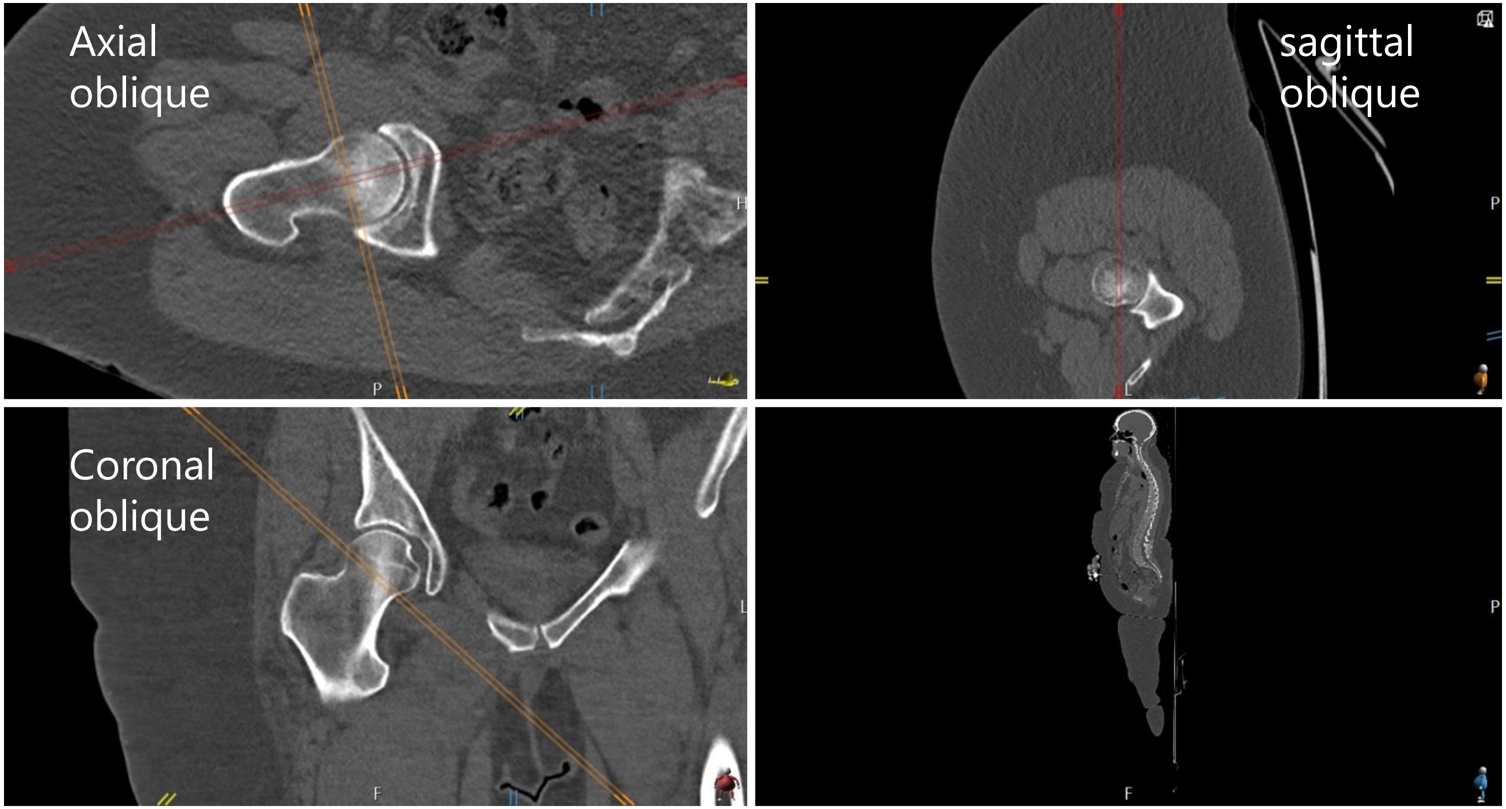

Joint Space Width hip

- Gravity and external rotation may somewhat narrow the inferoposterior joint space.

JSN	Definition
0	No JSN (<10% surface within 1.5mm)
1	Possible JSN (10-30% surface within 1.5mm)
2	Definite JSN (30-60% surface within 1.5mm)
3	Obliterated JS (>60% surface within 1.5mm)

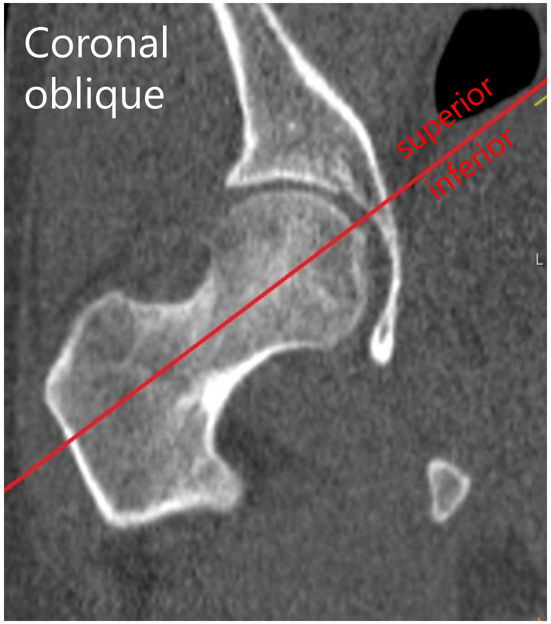

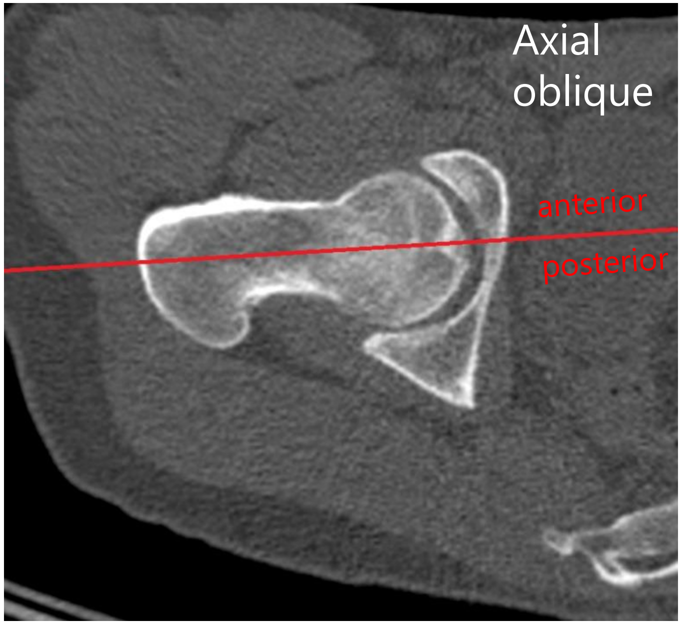

Hip JSN 0

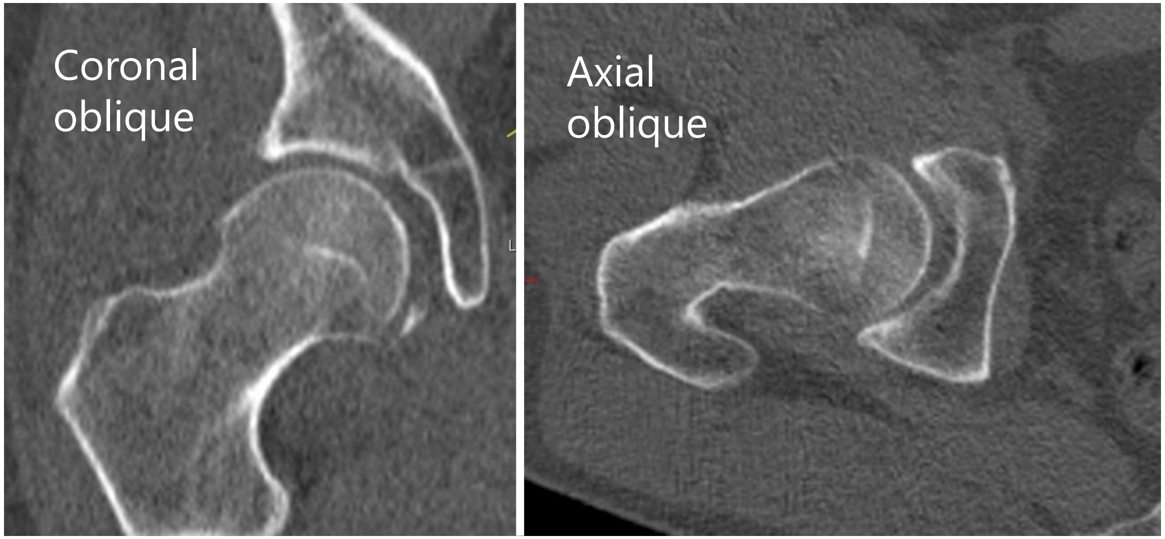

JSN	Definition
**0**	**No JSN (<10% surface within 1.5mm)**
1	Possible JSN (10-30% surface within 1.5mm)
2	Definite JSN (30-60% surface within 1.5mm)
3	Obliterated JS (>60% surface within 1.5mm)

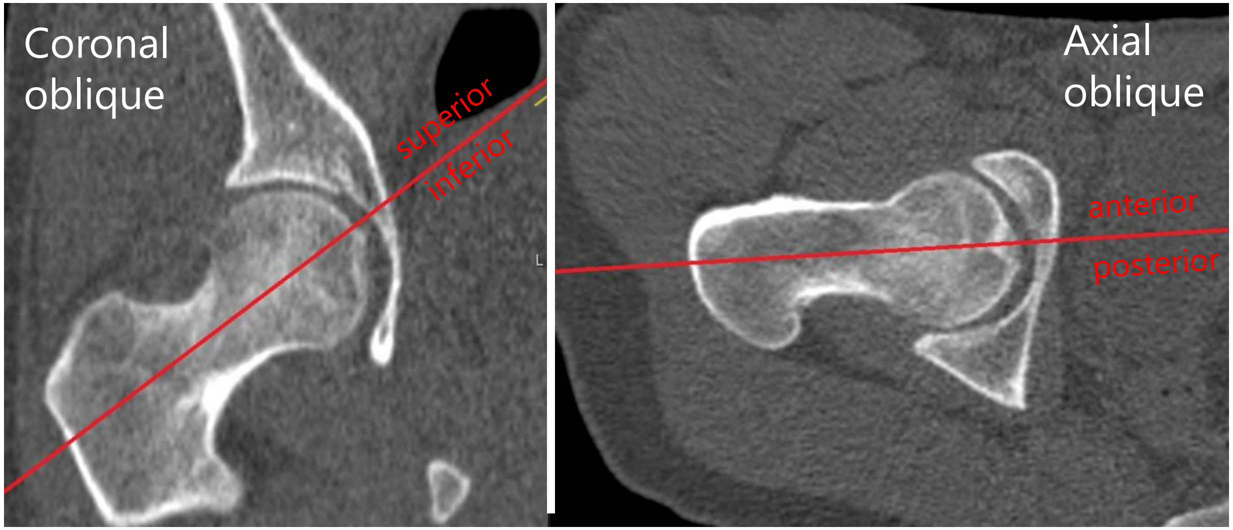

Hip JSN 1

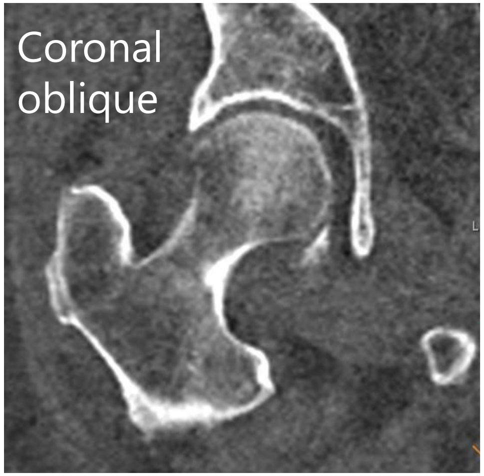

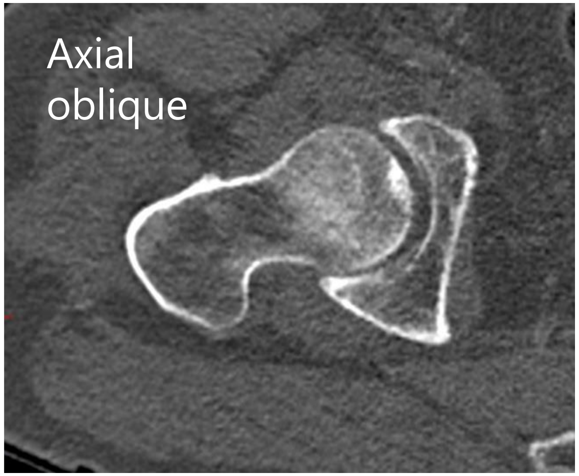

JSN	Definition
0	No JSN (<10% surface within 1.5mm)
**1**	**Possible JSN (10-30% surface within 1.5mm)**
2	Definite JSN (30-60% surface within 1.5mm)
3	Obliterated JS (>60% surface within 1.5mm)

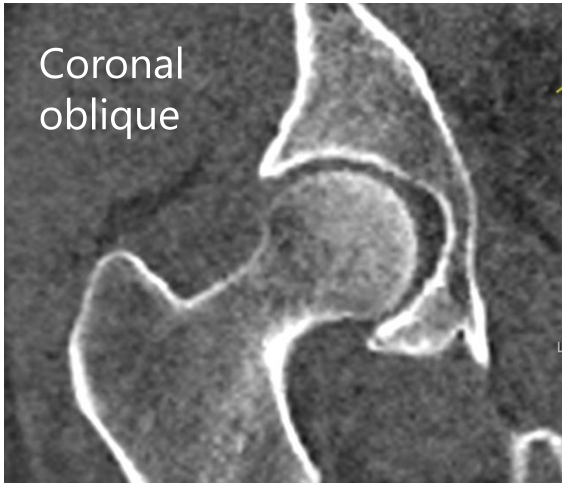

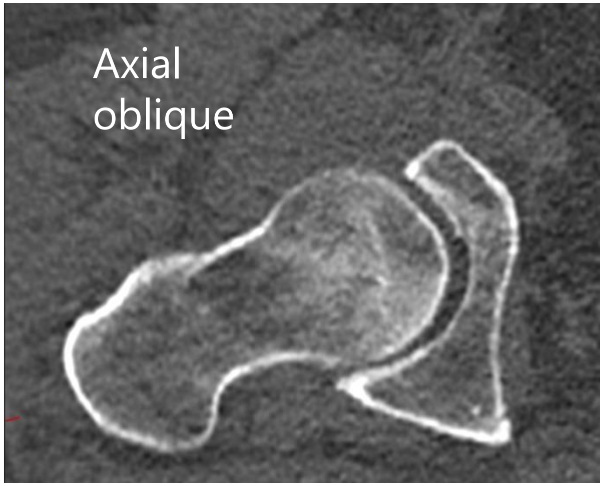

Hip JSN 2

JSN	Definition
0	No JSN (<10% surface within 1.5mm)
1	Possible JSN (10-30% surface within 1.5mm)
**2**	**Definite JSN (30-60% surface within 1.5mm)**
3	Obliterated JS (>60% surface within 1.5mm)

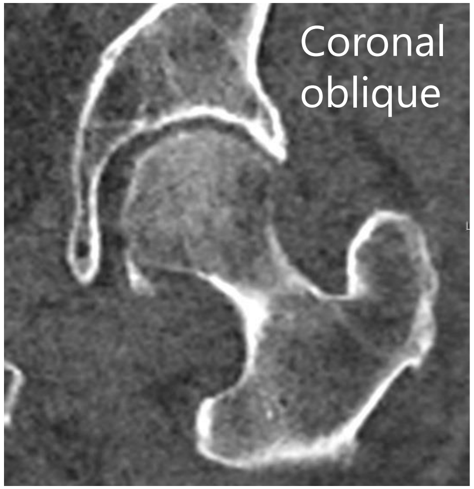

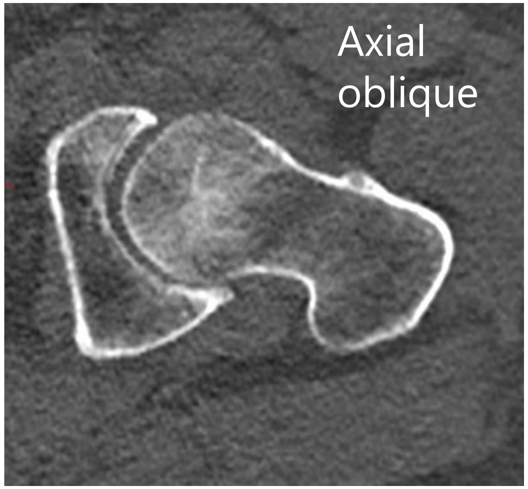

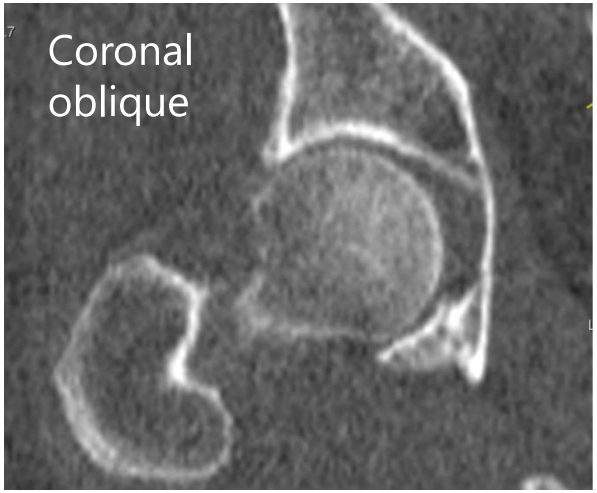

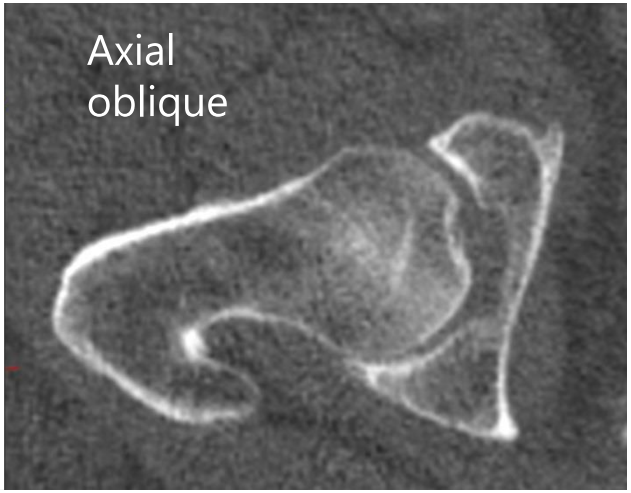

Hip JSN 3

JSN	Definition
0	No JSN (<10% surface within 1.5mm)
1	Possible JSN (10-30% surface within 1.5mm)
2	Definite JSN (30-60% surface within 1.5mm)
**3**	**Obliterated JS (>60% surface within 1.5mm)**

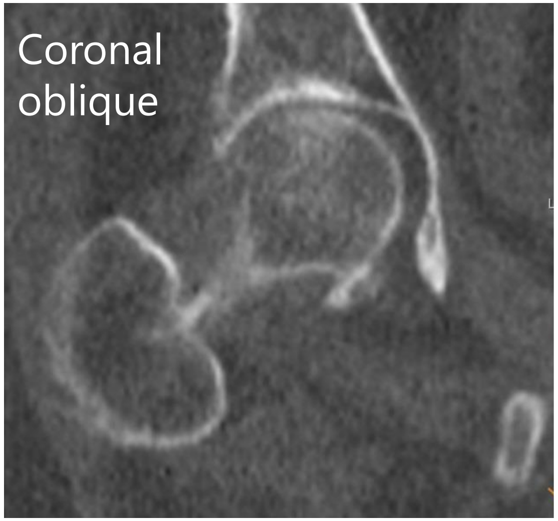

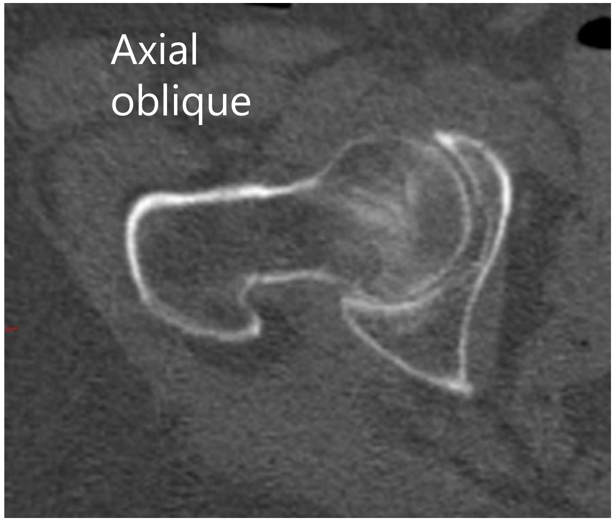

Hip Osteophytes

Image modified from Turmezei et al. 2014
“Severity mapping of the proximal femur: a new method for assessing hip osteoarthritis with computed tomography.”

Only scored in the femur, not in the acetabulum.

Mostly seen on the superior, posterior or inferior head neck junction

Osteophyte should be visible in at least 2 slices

Reaction area on the anterior portion of the head-neck junction of the femur are not considered osteophytes

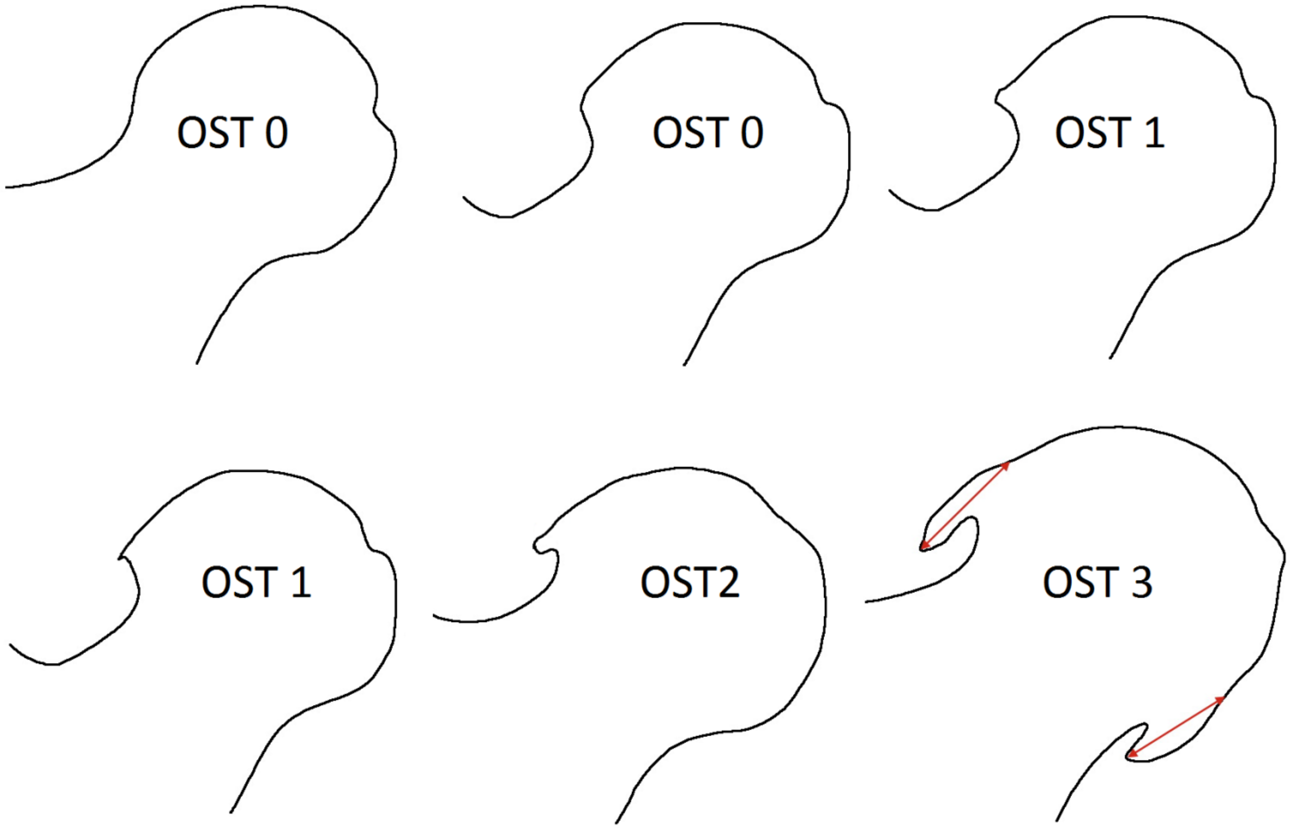

OST	Definition
0	No osteophyte
1	Possible / small osteophyte
2	Moderate osteophyte (≤ 5 mm)
3	Large osteophyte (> 5 mm)

Hip OST 0

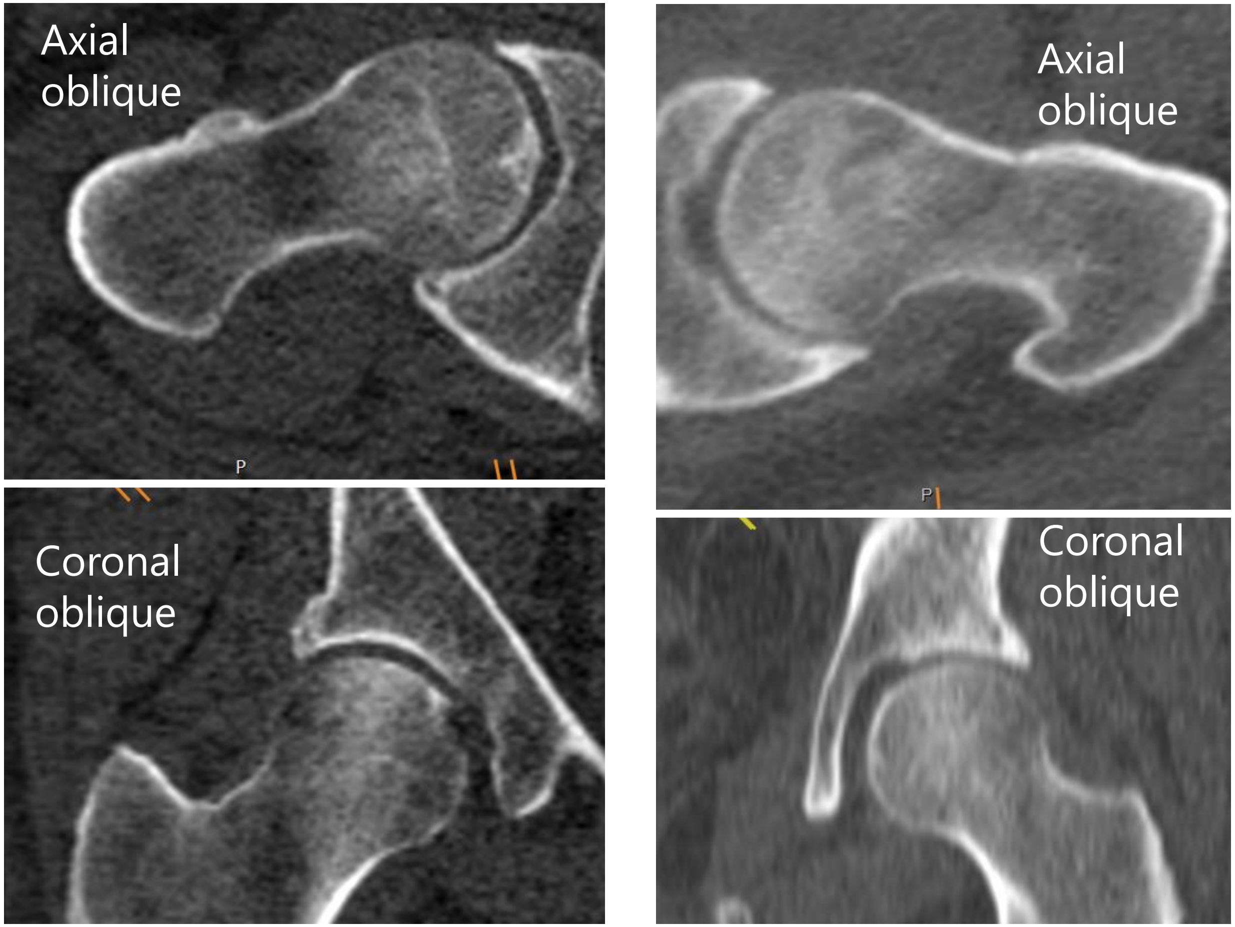

OST	Definition
**0**	**No osteophyte**
1	Possible / small osteophyte
2	Moderate osteophyte (≤ 5 mm)
3	Large osteophyte (> 5 mm)

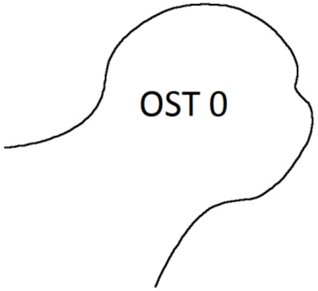

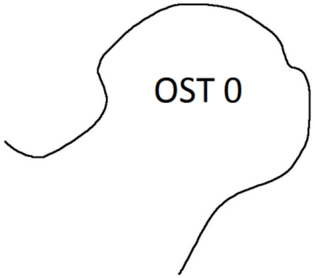

Hip OST 1

OST	Definition
0	No osteophyte
**1**	**Possible / small osteophyte**
2	Moderate osteophyte (≤ 5 mm)
3	Large osteophyte (> 5 mm)

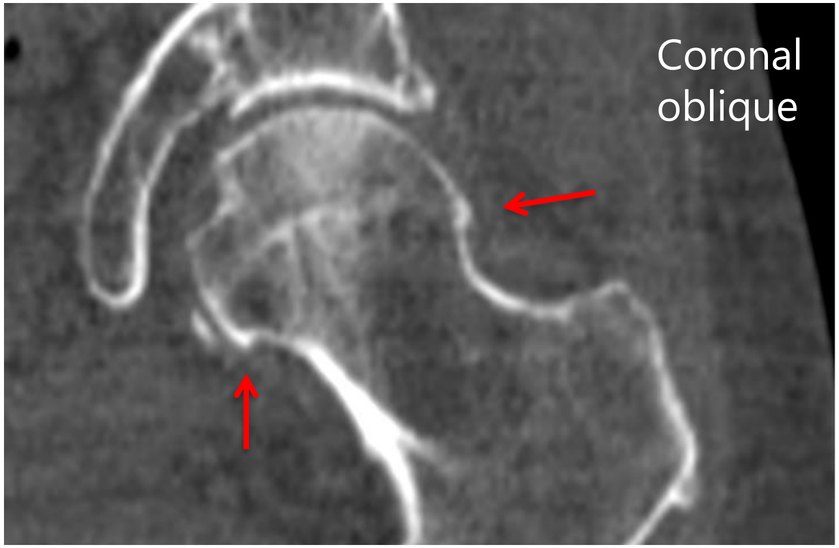

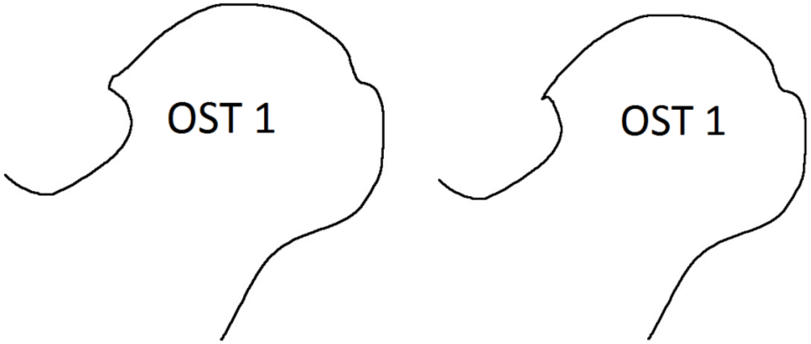

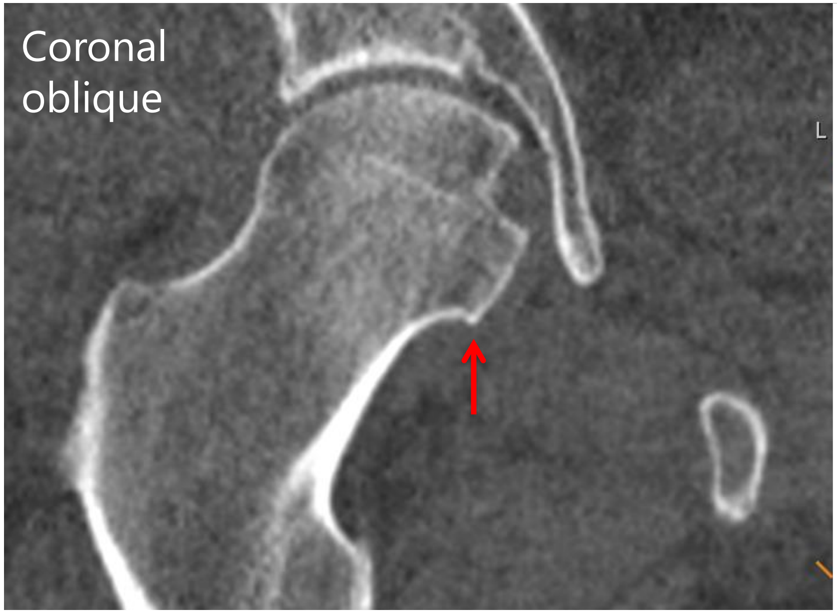

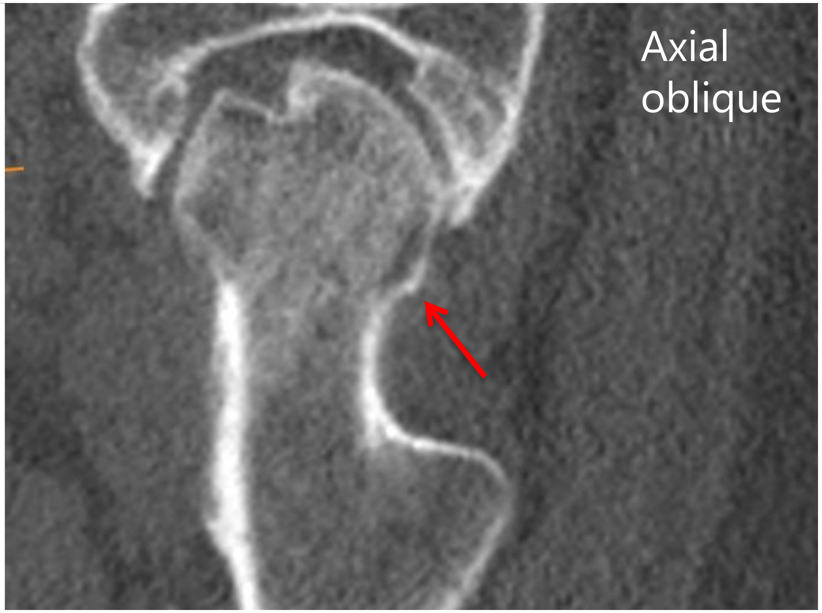

Hip OST 2

OST	Definition
0	No osteophyte
1	Possible / small osteophyte
**2**	**Moderate osteophyte (≤ 5 mm)**
3	Large osteophyte (> 5 mm)

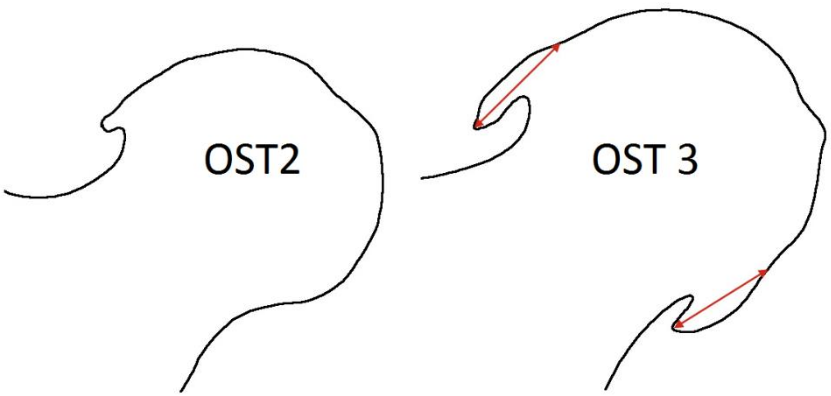

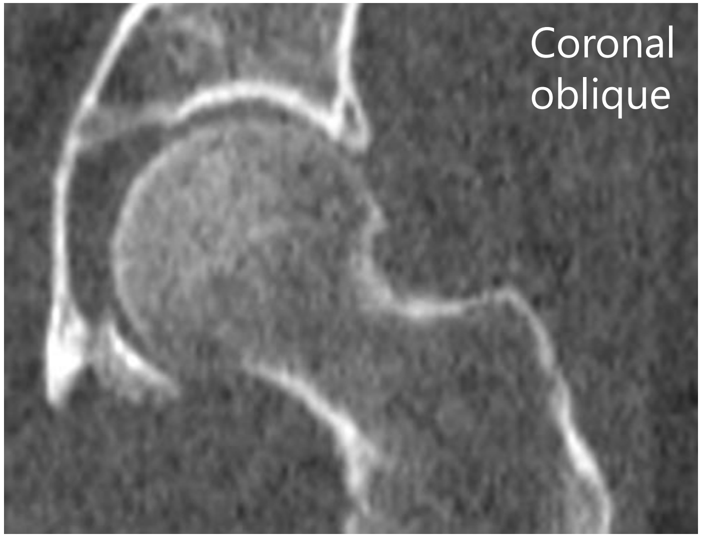

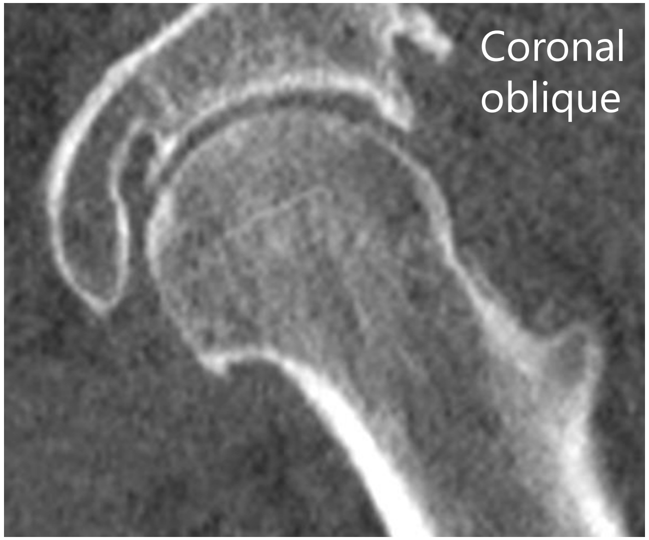

Hip OST 3

OST	Definition
0	No osteophyte
1	Possible / small osteophyte
2	Moderate osteophyte (≤ 5 mm)
**3**	**Large osteophyte (> 5 mm)**

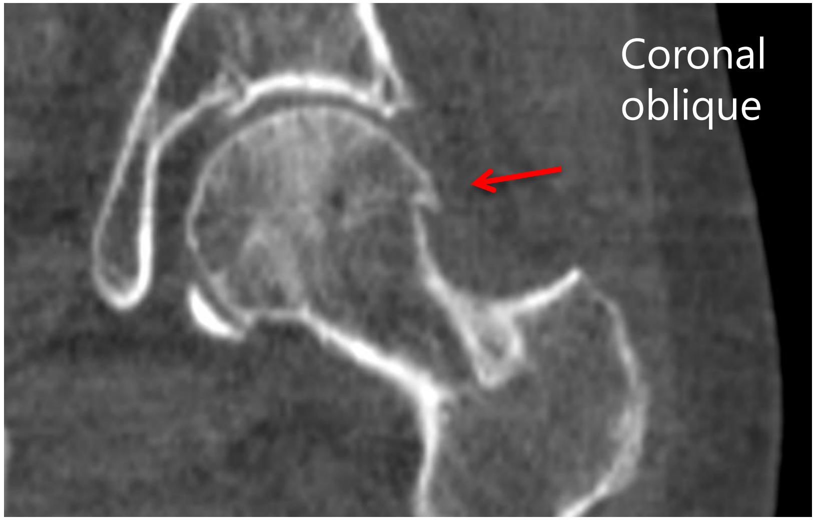

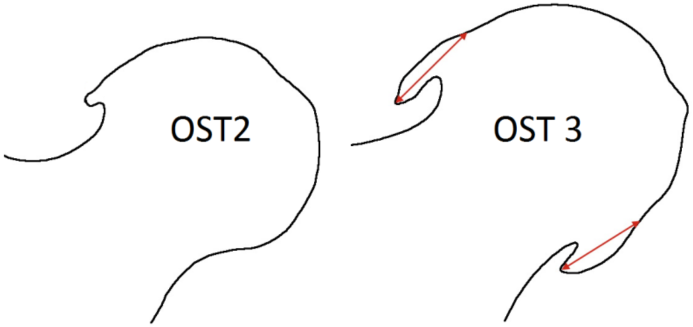

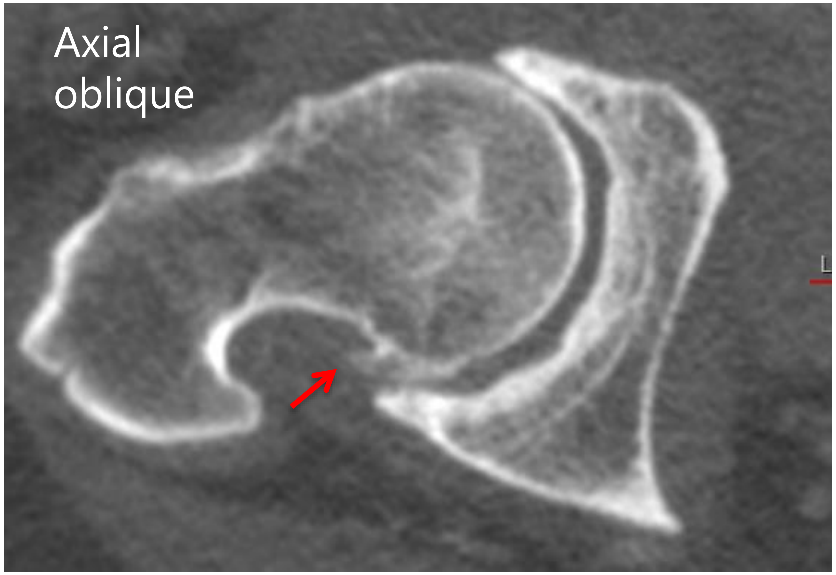

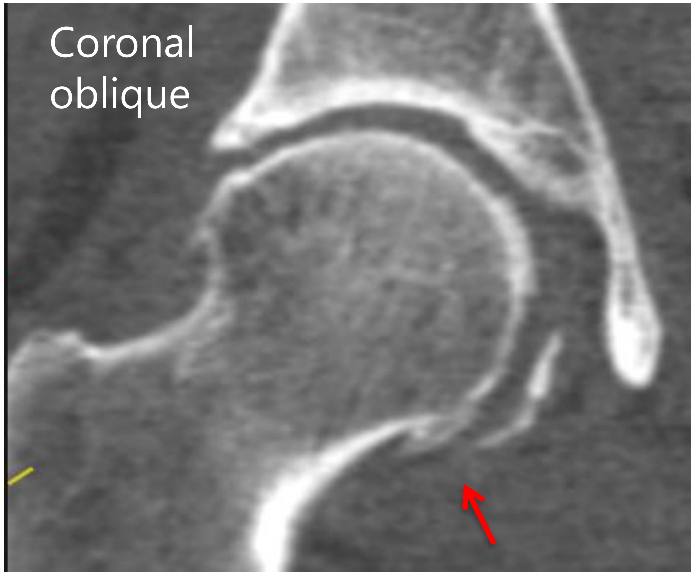

Hip Cysts

- Cysts are scored as present or not
  - Cyst should be present in the subchondral bone
  - Reactional cyst should not be scored

Cyst	Definition
0	No subchondral cysts or criteria grade 1 not met
1	Single cyst, maximal diameter > 5mm or<br>Multiple cysts, maximal diameter ≤ 5 mm in same region

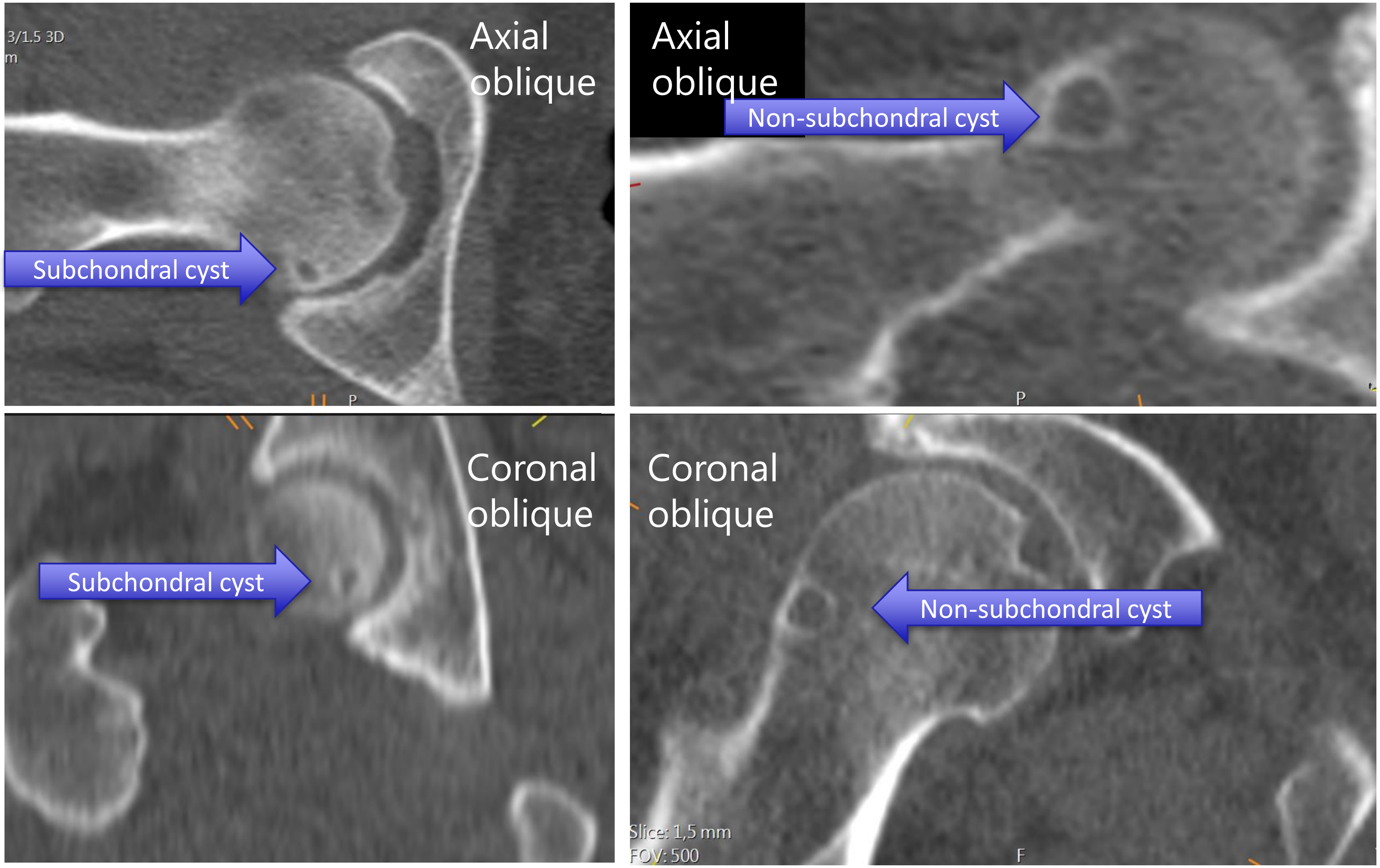

Hip OA total score

- Joint space narrowing 0-3
- Osteophyte score 0-3
- Cyst 0-1 +
- Total 0-7

- Grade 0 0-1
- Grade 1 2-3
- Grade 2 4-5
- Grade 3 6-7

Tibiofemoral joint (Knee)

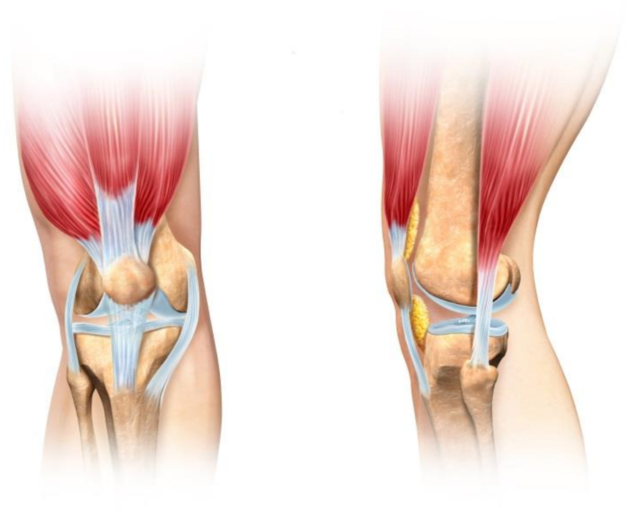

Rotate the MPR so that the sagittal plain runs trough the middle of the patella and the coronal plain is parallel to the posterior aspect of the condyles

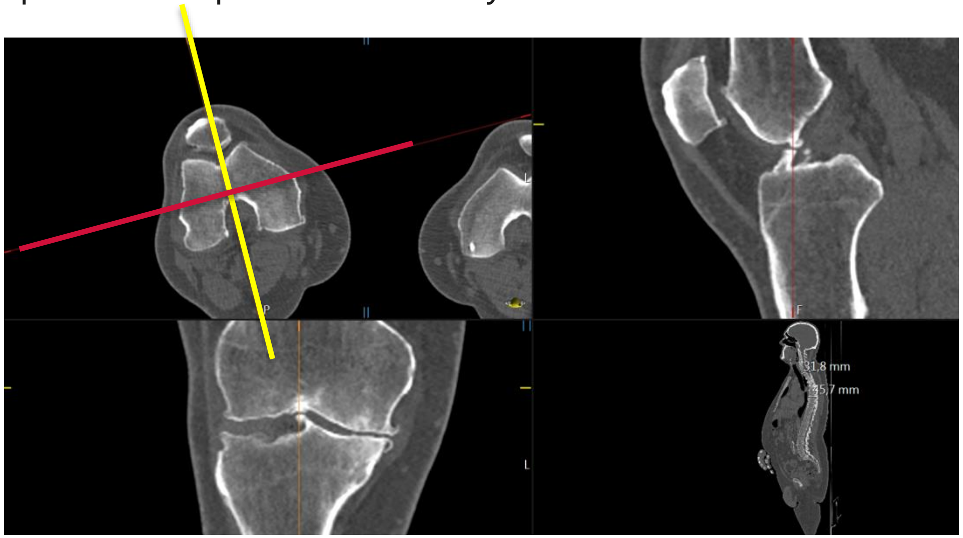

	Tibiofemoral joint (Knee)
Predominant view	Coronal, axial and sagittal<br>Oblique if needed
Settings	Multiplanar view, minimal slice thickness, bone window
Sections	Left and Right
Grading	3 grades per section<br>Joint Space Narrowing (JSN)<br>Osteophytes (OST)<br>Cysts (CYST)
**JSN**	**Definition**
0	No joint space narrowing
1	Possible joint space narrowing
2	Definite joint space narrowing
3	Obliterated joint space
**OST**	**Definition**
0	No osteophyte
1	Possible / small osteophyte
2	Moderate osteophyte (≤ 5 mm)
3	Large osteophyte (> 5 mm)
**CYST**	**Definition**
0	No subchondral cyst
1	Presence of subchondral cyst

Illustration from GettyImages

Tibiofemoral JSN 0

JSN	Definition
**0**	**No joint space narrowing**
1	Possible joint space narrowing
2	Definite joint space narrowing
3	Obliterated joint space

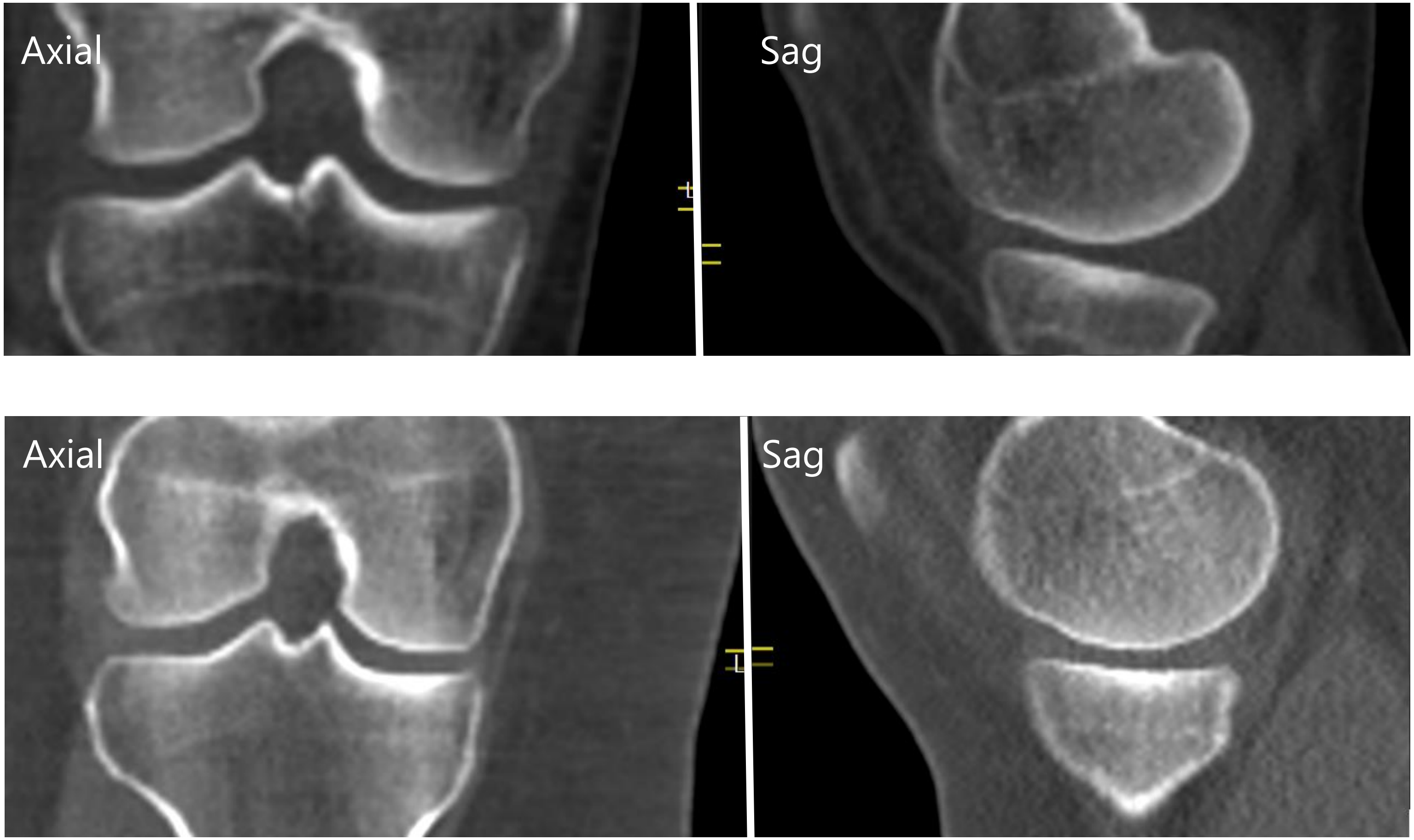

Tibiofemoral JSN 1

JSN	Definition
0	No joint space narrowing
**1**	**Possible joint space narrowing**
2	Definite joint space narrowing
3	Obliterated joint space

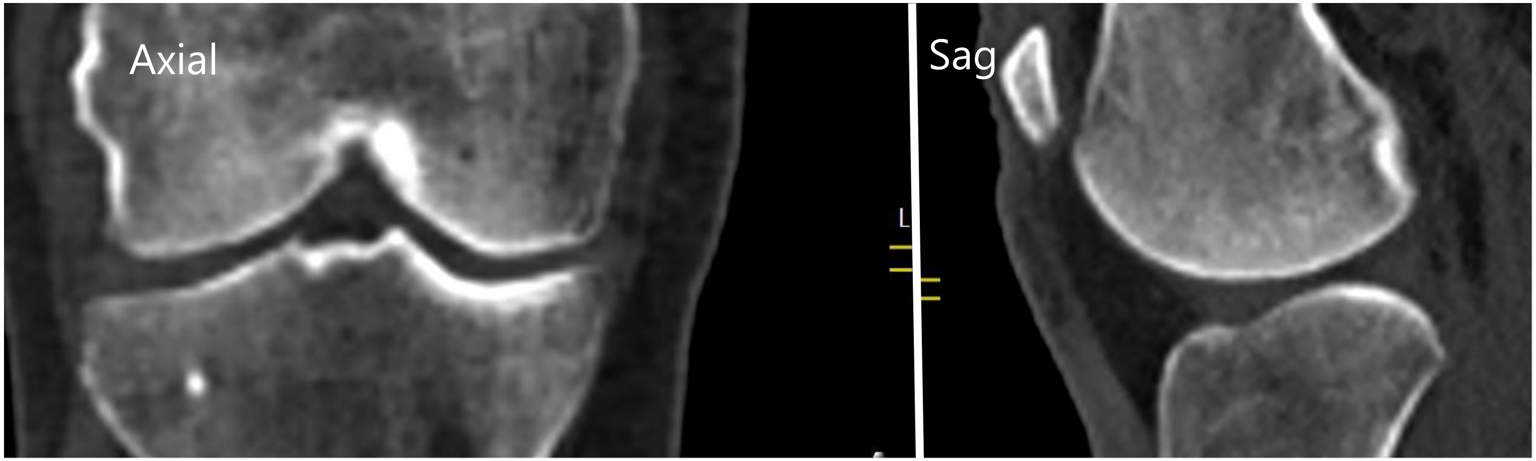

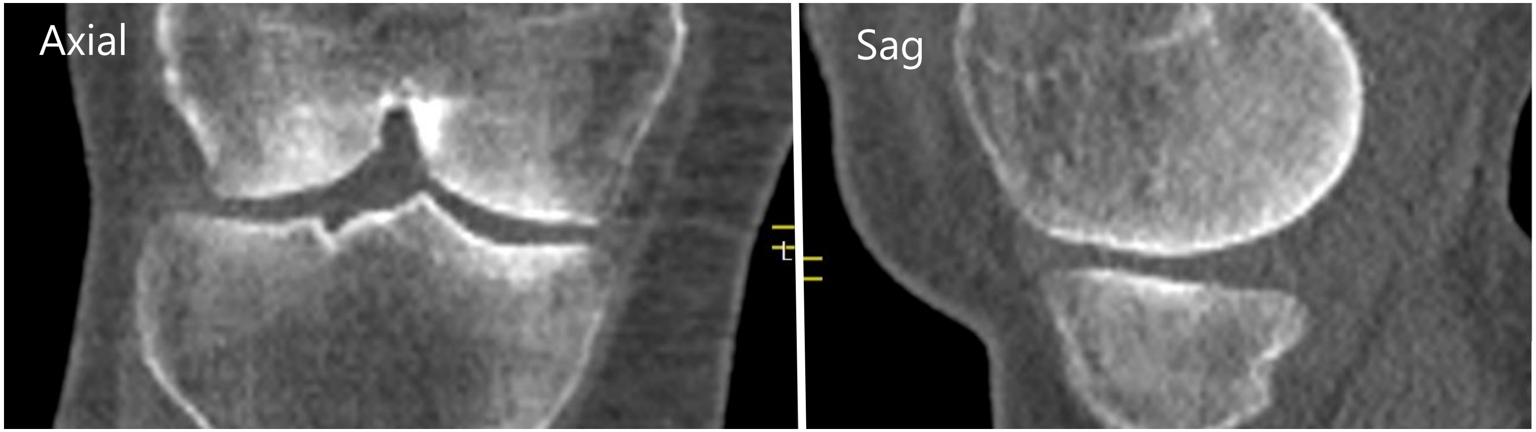

Tibiofemoral JSN 2

JSN	Definition
0	No joint space narrowing
1	Possible joint space narrowing
**2**	**Definite joint space narrowing**
3	Obliterated joint space

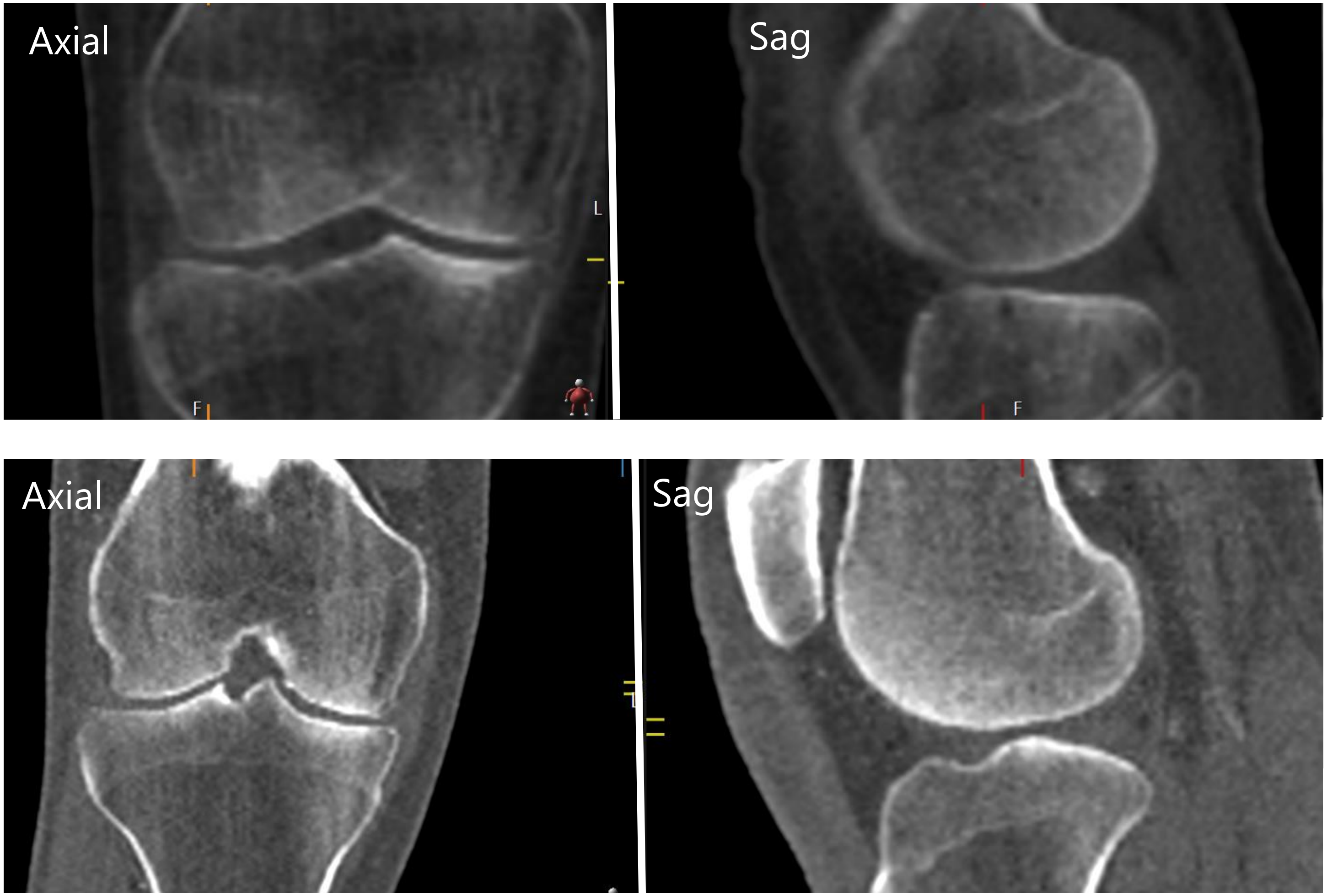

Tibiofemoral JSN 3

JSN	Definition
0	No joint space narrowing
1	Possible joint space narrowing
2	Definite joint space narrowing
**3**	**Obliterated joint space**

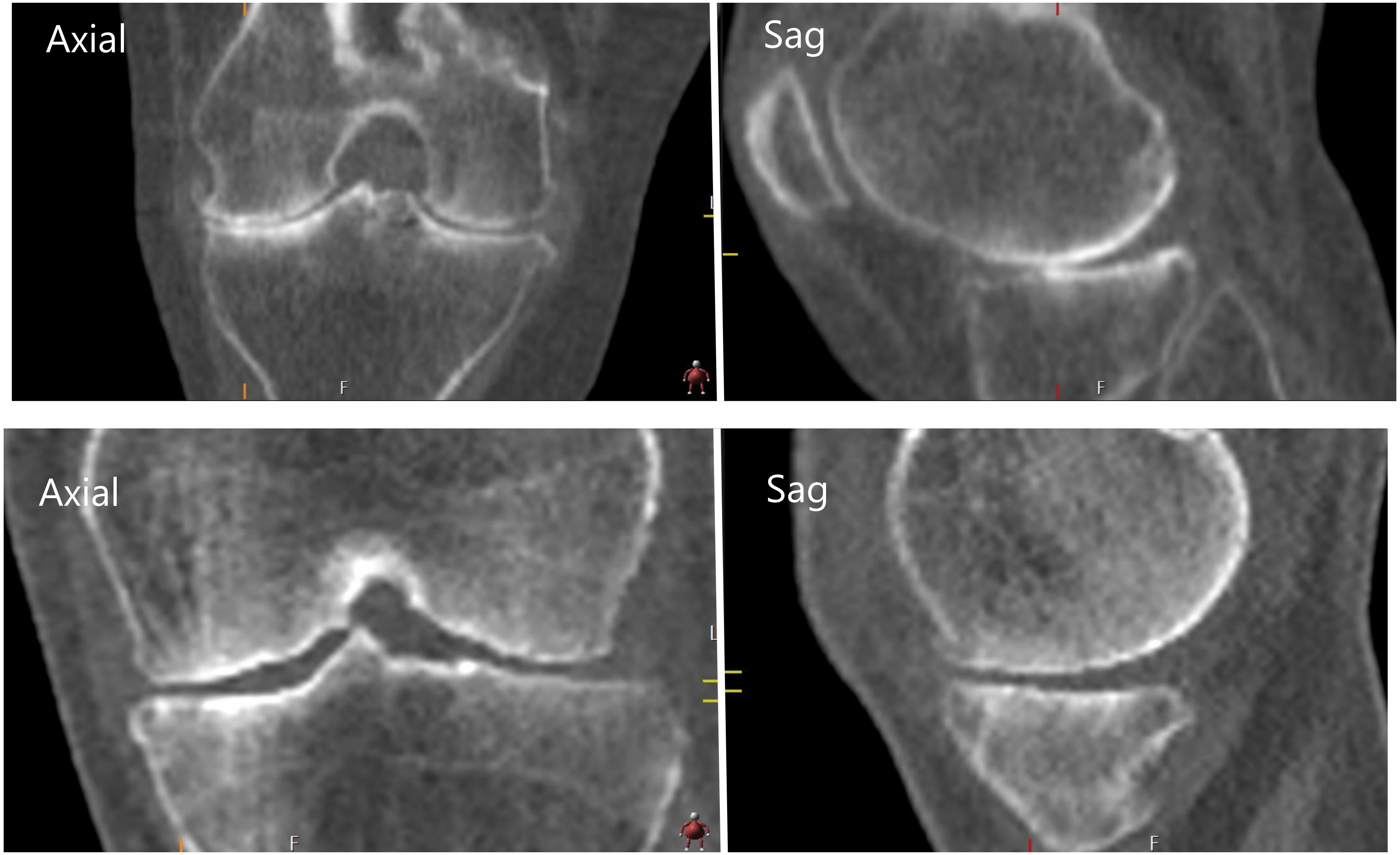

Tibiofemoral Osteophytes 1

OST	Definition
0	No osteophyte
**1**	**Lipping / small osteophyte**
2	Clear moderate osteophyte
3	Clear large osteophyte

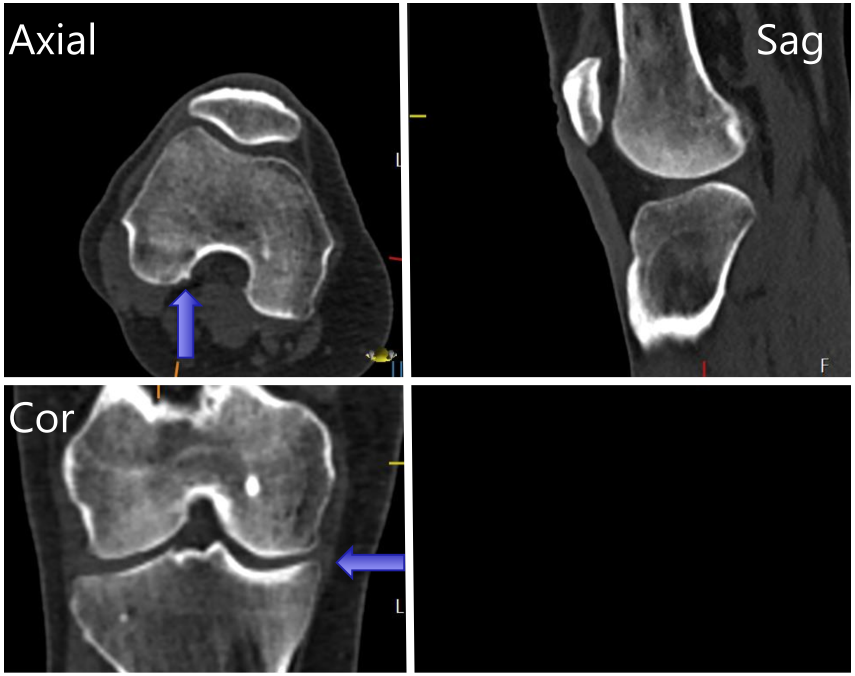

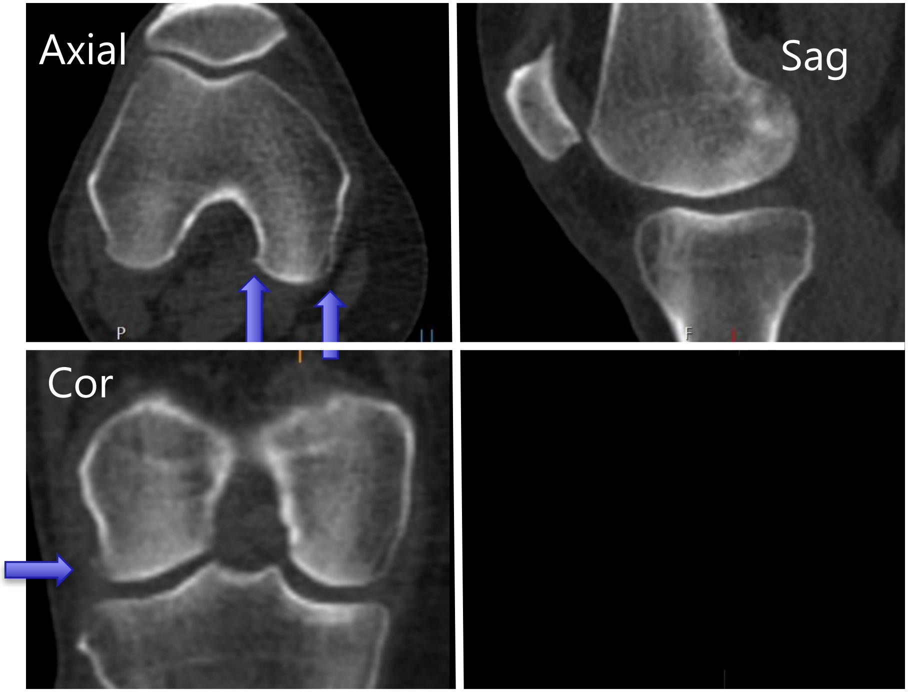

Tibiofemoral Osteophytes 2

OST	Definition
0	No osteophyte
1	Lipping / small osteophyte
**2**	**Clear moderate osteophyte**
3	Clear large osteophyte

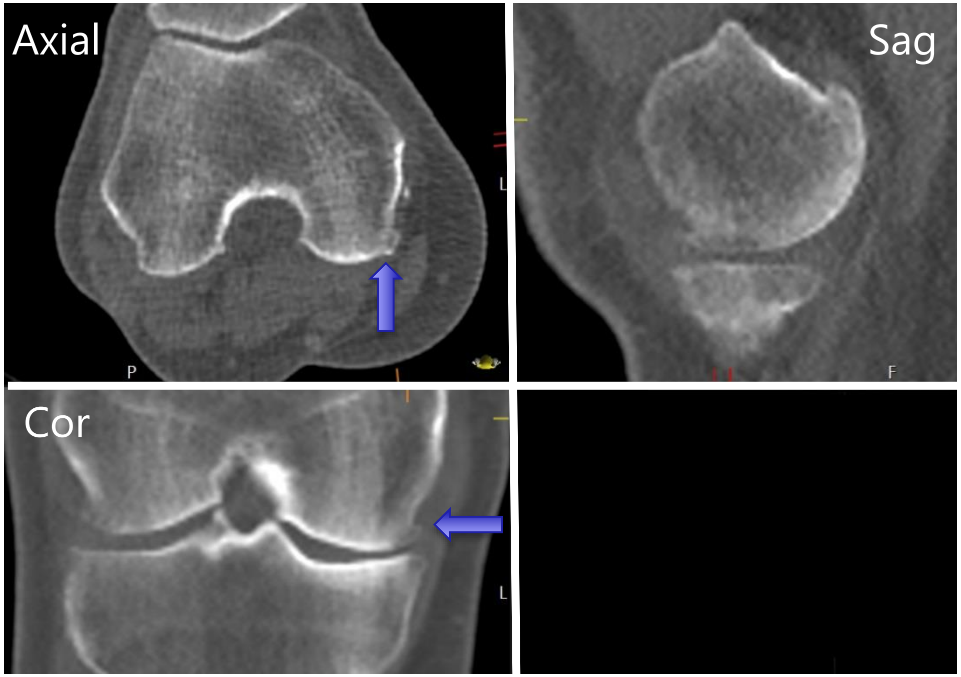

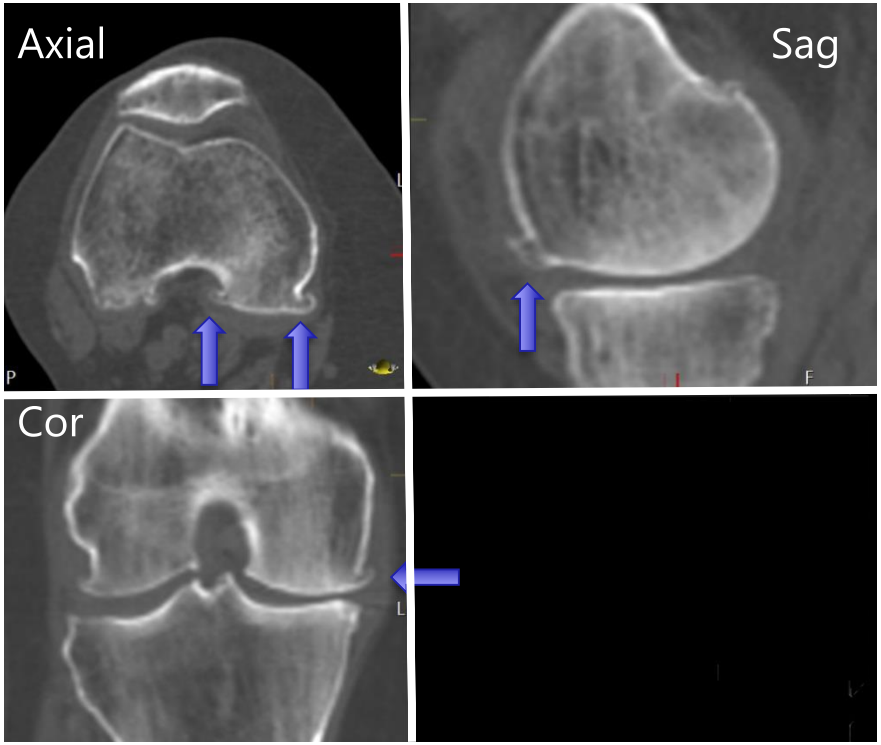

Tibiofemoral Osteophytes 3

OST	Definition
0	No osteophyte
1	Lipping / small osteophyte
2	Clear moderate osteophyte
**3**	**Clear large osteophyte**

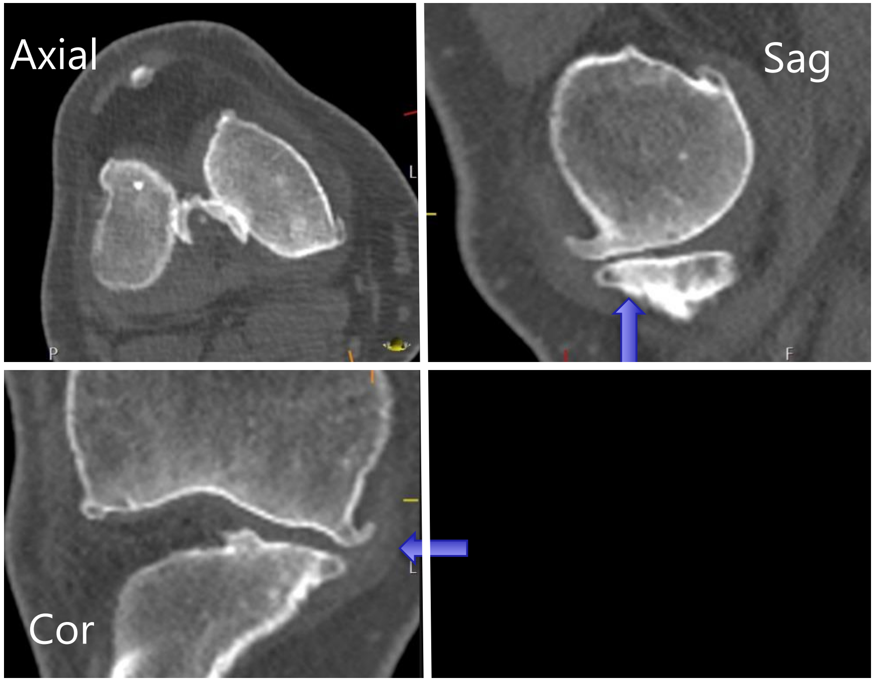

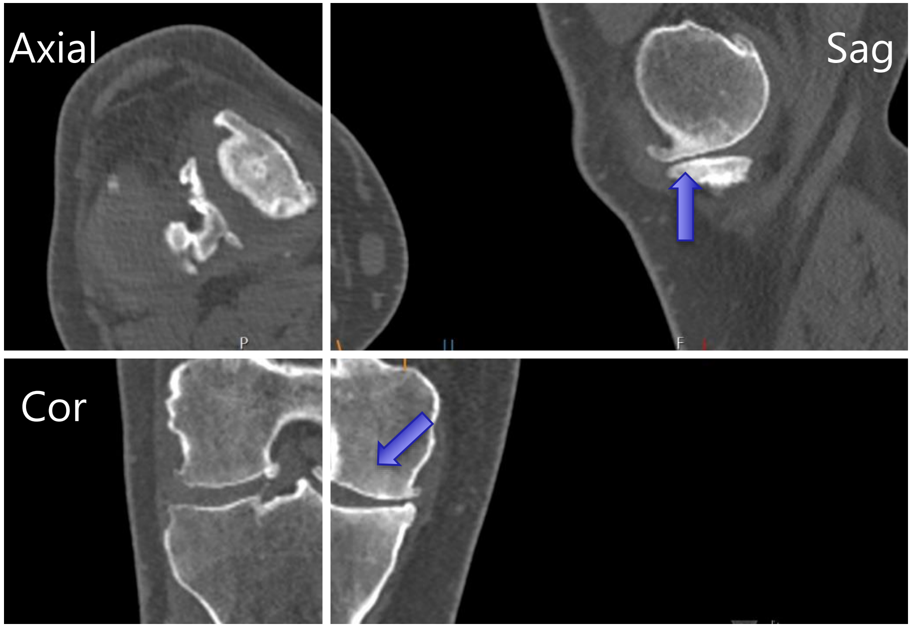

Tibiofemoral Subchondral Cyst 1

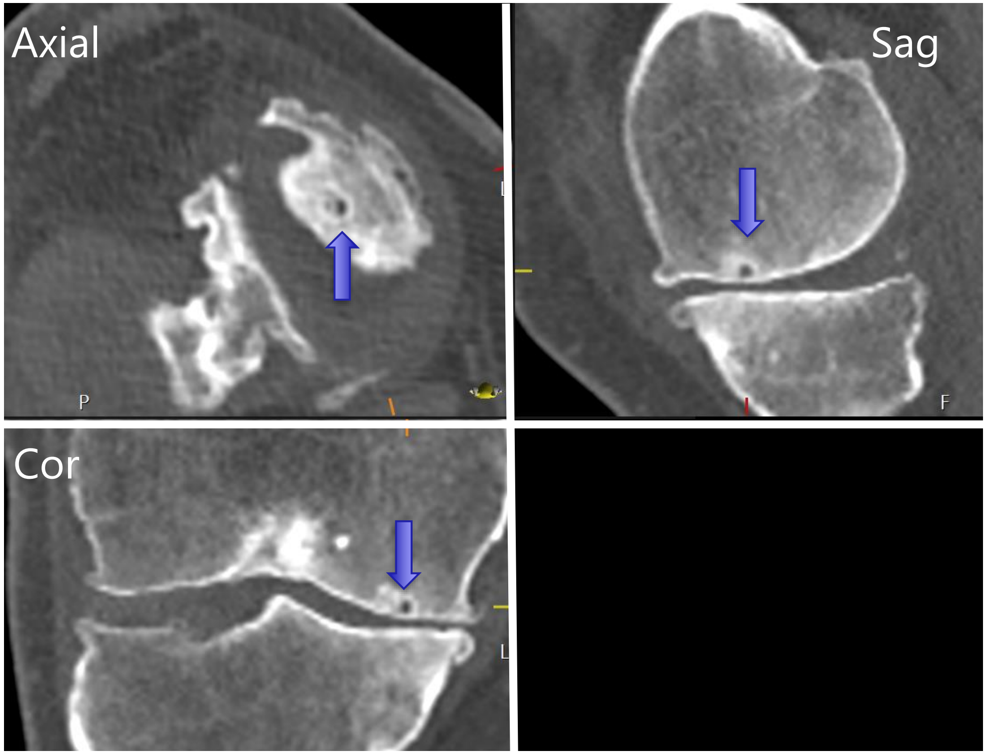

CYST	Definition
0	No subchondral cyst
**1**	**Presence of subchondral cyst**

Tibiofemoral OA total score

• Joint space narrowing	0-3
• Osteophyte score	0-3
• Cyst	0-1 +
• Total	0-7

• Grade 0	0-1
• Grade 1	2-3
• Grade 2	4-5
• Grade 3	6-7

Patellofemoral joint (PF)

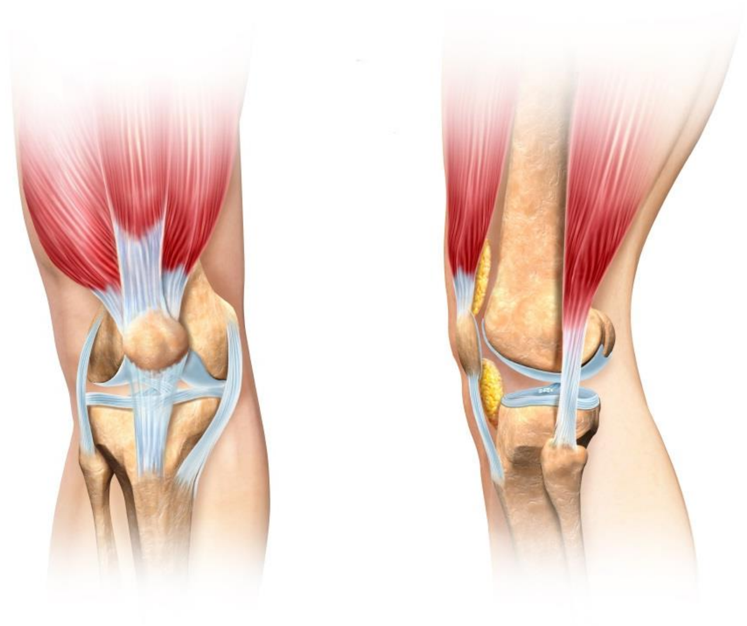

	Patellofemoral joint (Knee)
Predominant view	Axial and sagittal
Settings	Multiplanar view, minimal slice thickness, bone window
Sections	Left and Right
Grading	1 grades per section
**PF**	**Definition**
0	No osteophytes or sclerosis
1	Small osteophyte/lipping, but no sclerosis
2	Moderate osteophytes and mild sclerosis
3	Large osteophytes, (near) boney contact and definiete sclerosis

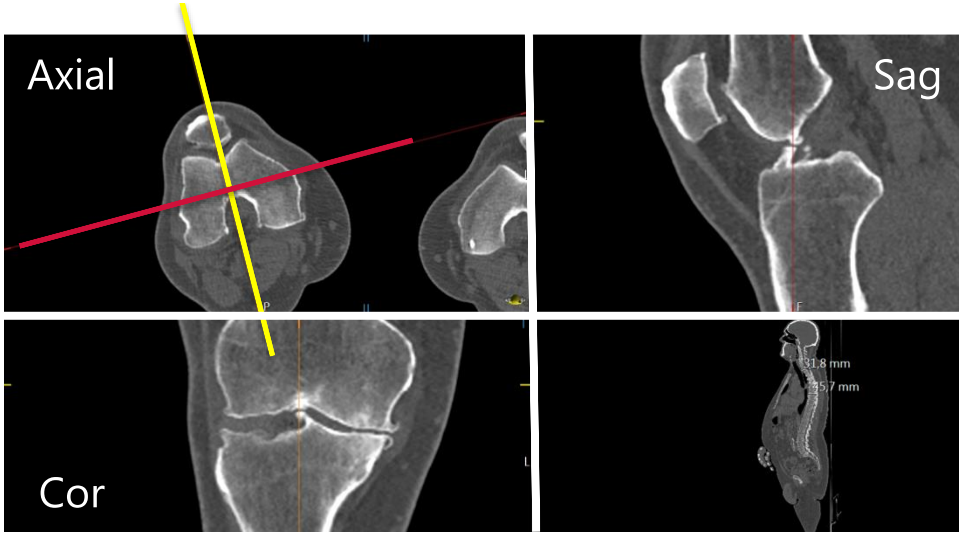

Rotate the MPR so that the sagittal plain runs trough the middle of the patella and the coronal plain is parallel to the posterior aspect of the condyls

Illustration from GettyImages

PF 0

PF	Definition
**0**	**No osteophytes or sclerosis**
1	Small osteophyte/lipping, but no sclerosis
2	Moderate osteophytes and mild sclerosis
3	Large osteophytes, (near) boney contact and definiete sclerosis

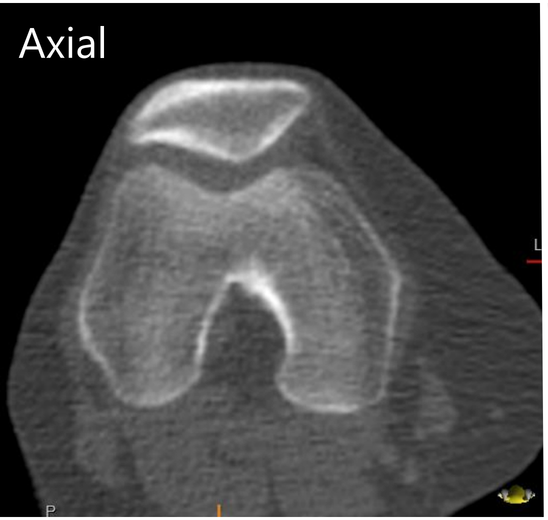

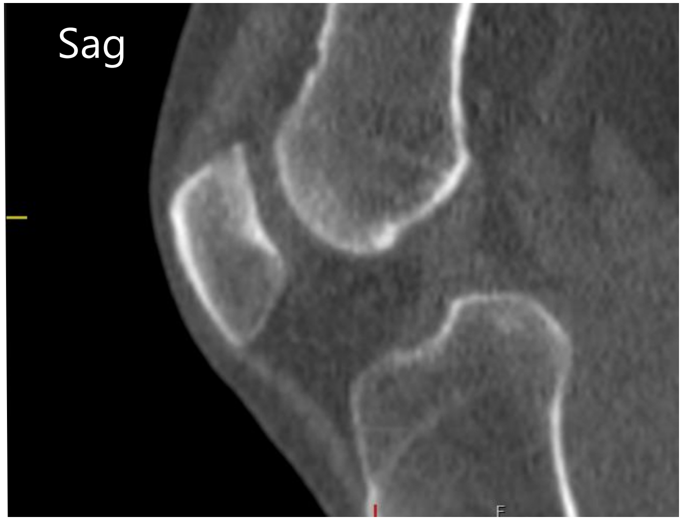

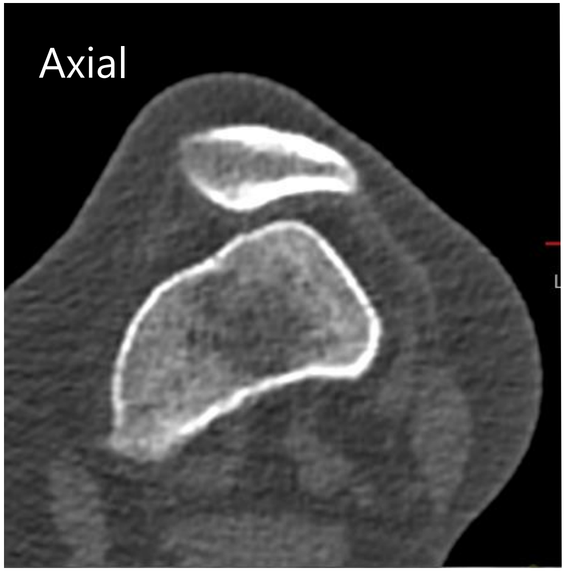

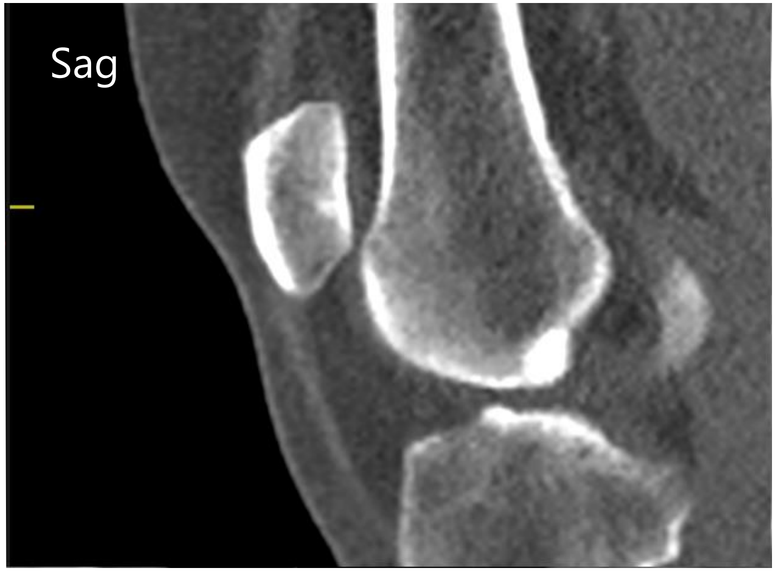

PF 1

PF	Definition
0	No osteophytes or sclerosis
**1**	**Small osteophyte/lipping, but no sclerosis**
2	Moderate osteophytes and mild sclerosis
3	Large osteophytes, (near) boney contact and definiete sclerosis

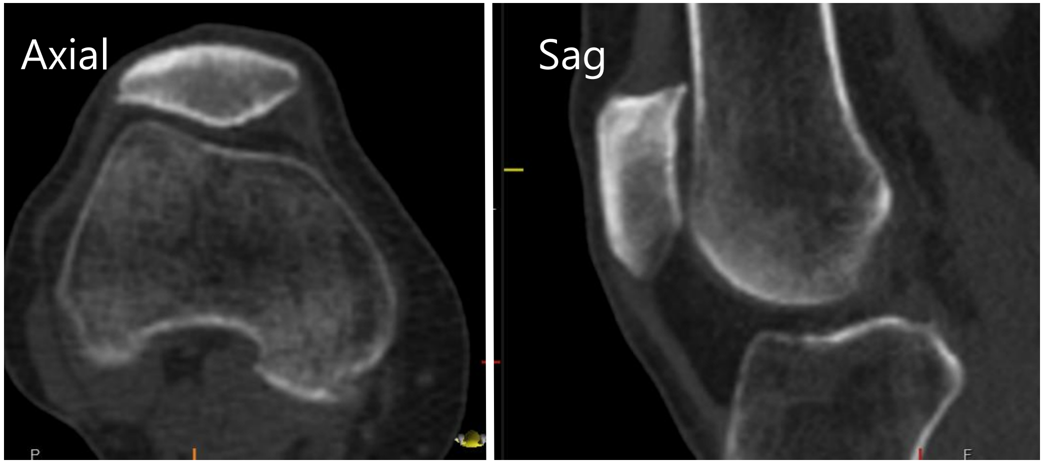

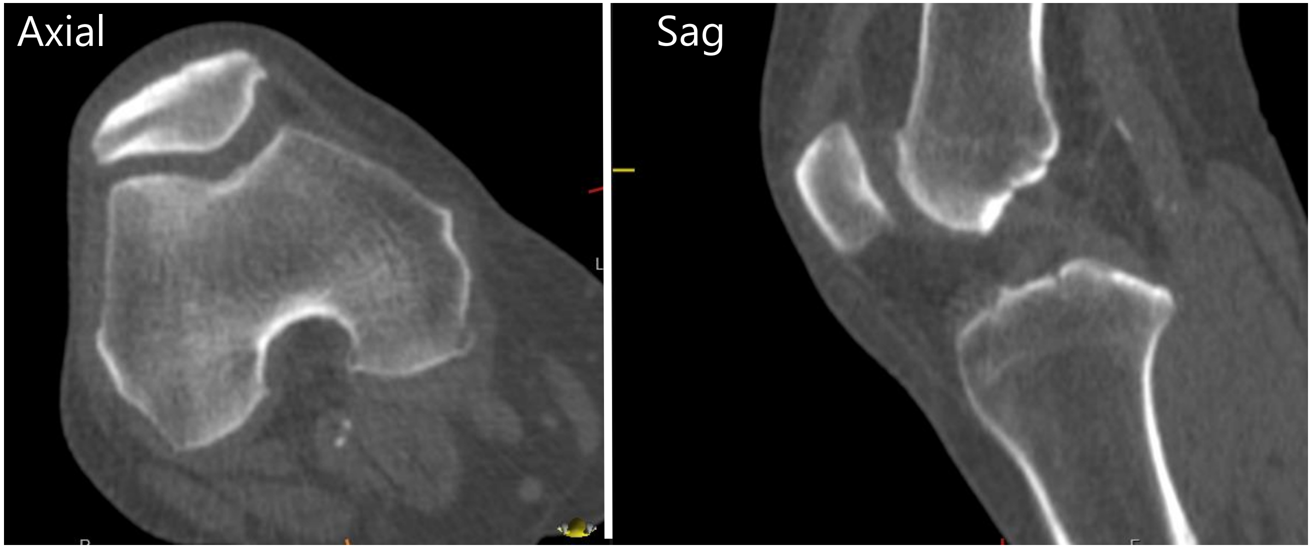

PF 2

PF	Definition
0	No osteophytes or sclerosis
1	Small osteophyte/lipping, but no sclerosis
**2**	**Moderate osteophytes and mild sclerosis**
3	Large osteophytes, (near) boney contact and definiete sclerosis

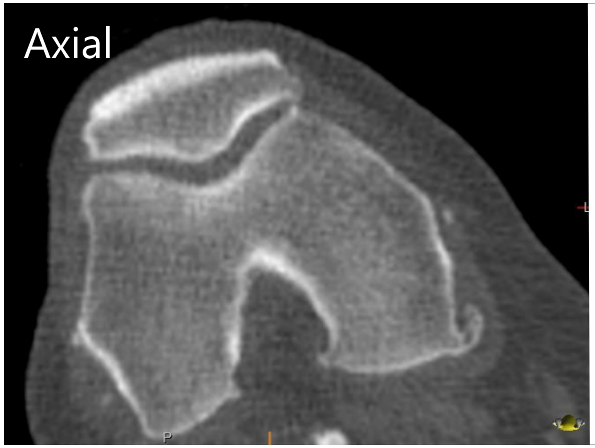

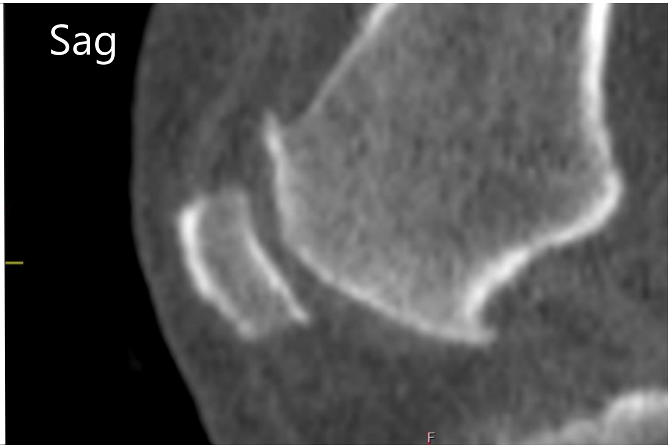

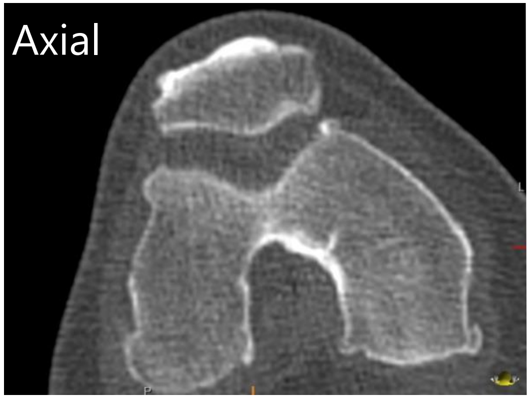

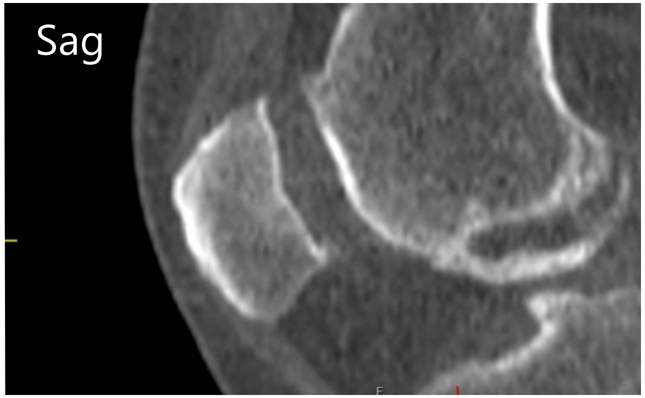

PF 3

PF	Definition
0	No osteophytes or sclerosis
1	Small osteophyte/lipping, but no sclerosis
2	Moderate osteophytes and mild sclerosis
**3**	**Large osteophytes, (near) boney contact and definiete sclerosis**

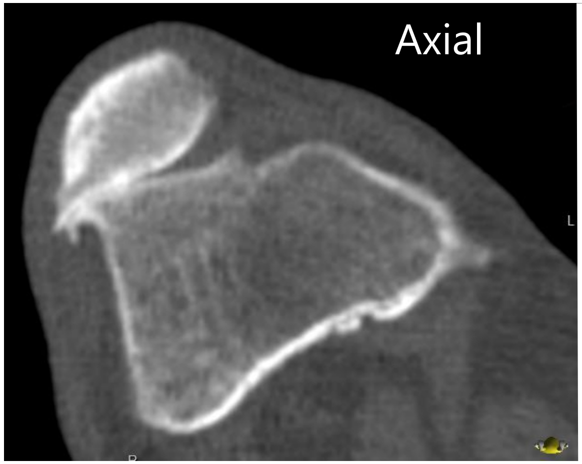

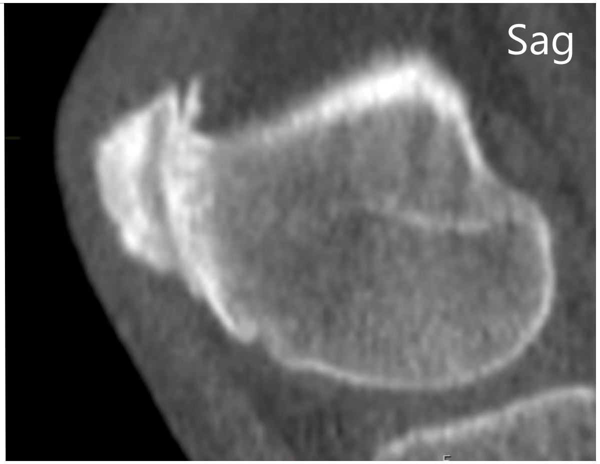

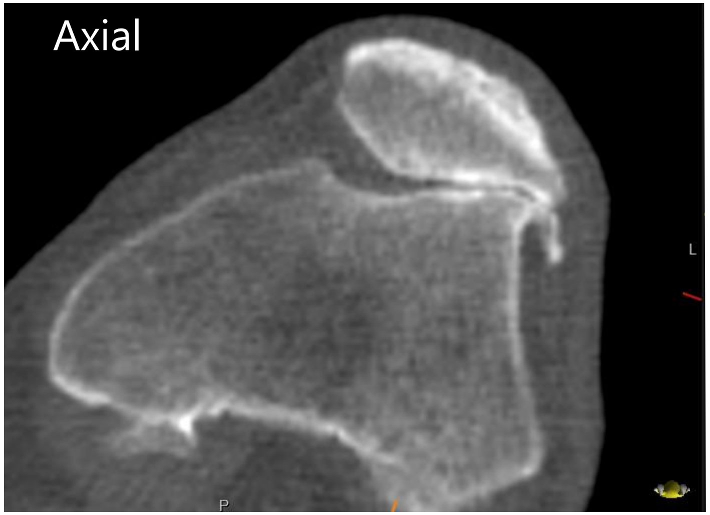

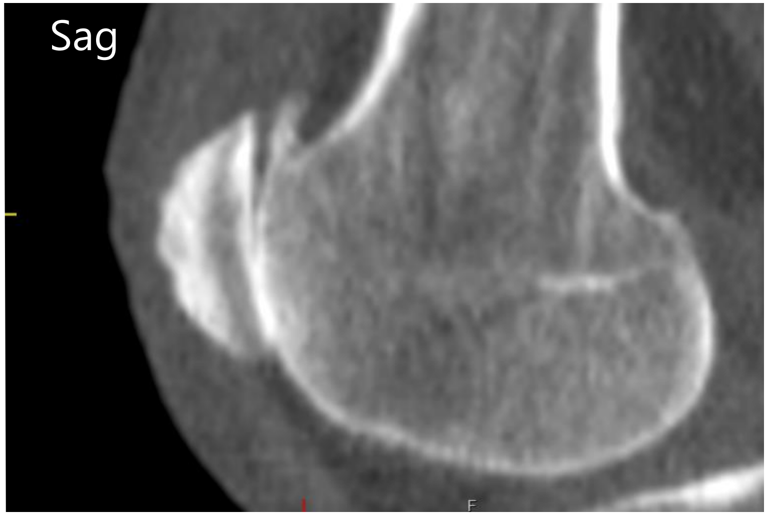

Talocrural joint (Ankle)

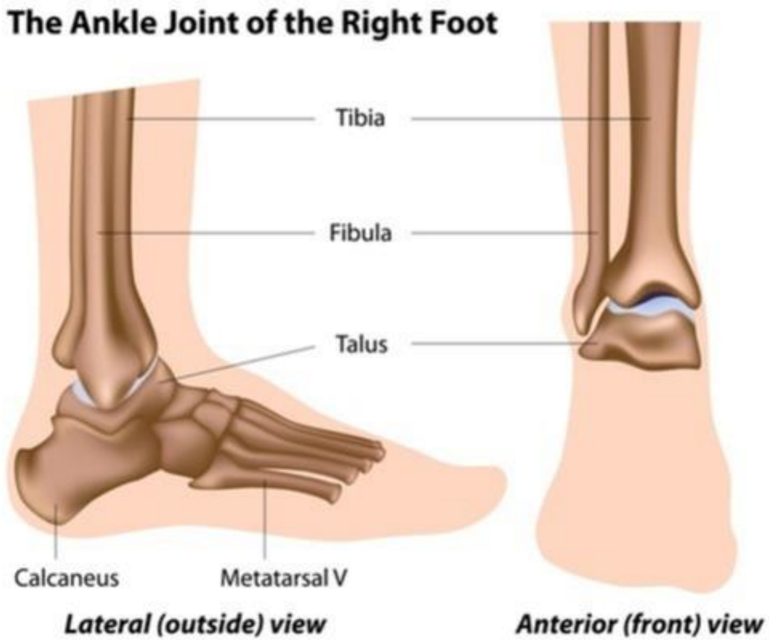

	Talocrural joint (Ankle)
Predominant view	Coronal, axial and sagittal<br>Oblique if needed
Settings	Multiplanar view, minimal slice thickness, bone window
Sections	Left and Right
Grading	Single grade per section
**Ankle**	**Definition**
0	No clinical evidence of OA; joint space integrity fully intact
1	Mild; osteophyte formation/lipping, possible joint space narrowing
2	Moderate; joint space narrowing evident, obvious osteophyte formation and some sclerosis/cystic changes
3	Severe; near absence of joint space, severe osteophyte/cyst formation, deformity of bone

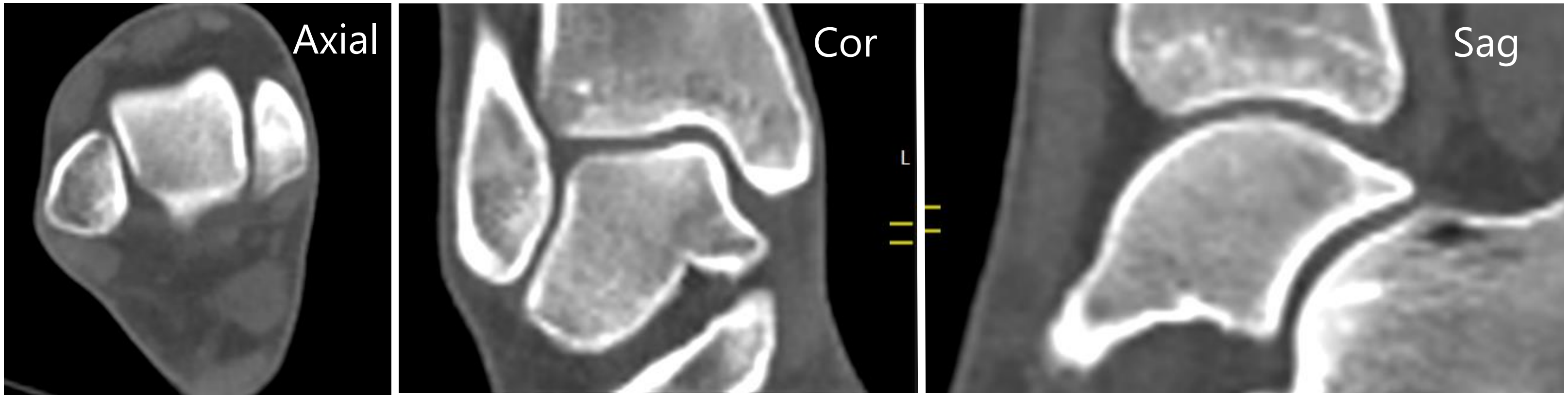

Illustration from Shutterstock
Score described by Cohen et al. 2015

Ankle 0

Ankle	Definition
**0**	**No clinical evidence of OA; joint space integrity fully intact**
1	Mild; osteophyte formation/lipping, possible joint space narrowing
2	Moderate; joint space narrowing evident, obvious osteophyte formation and some sclerosis/cystic changes
3	Severe; near absence of joint space, severe osteophyte/cyst formation, deformity of bone

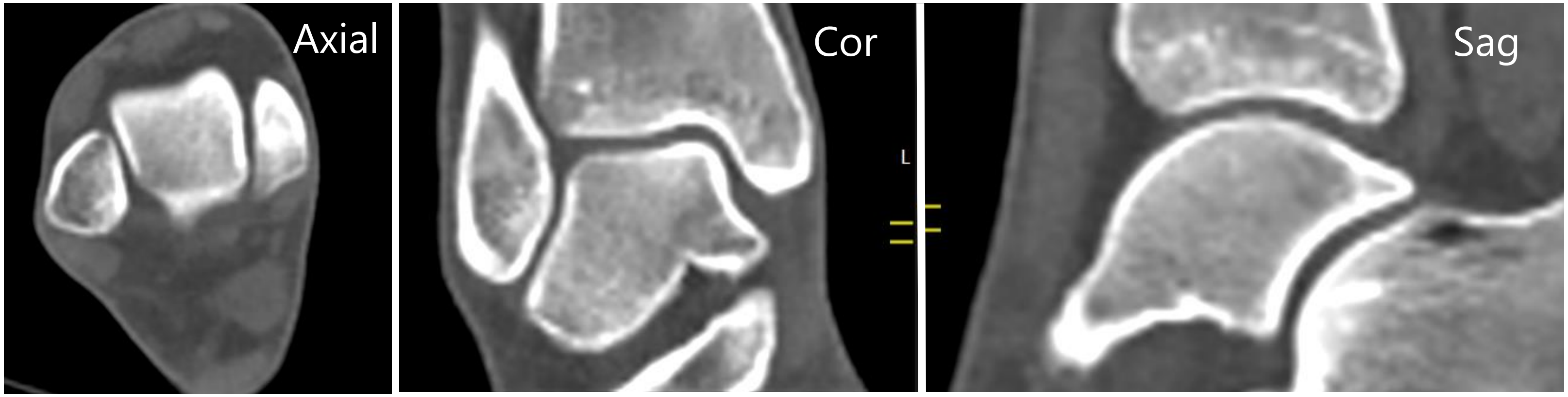

Ankle 1

Ankle	Definition
0	No clinical evidence of OA; joint space integrity fully intact
**1**	**Mild; osteophyte formation/lipping, possible joint space narrowing**
2	Moderate; joint space narrowing evident, obvious osteophyte formation and some sclerosis/cystic changes
3	Severe; near absence of joint space, severe osteophyte/cyst formation, deformity of bone

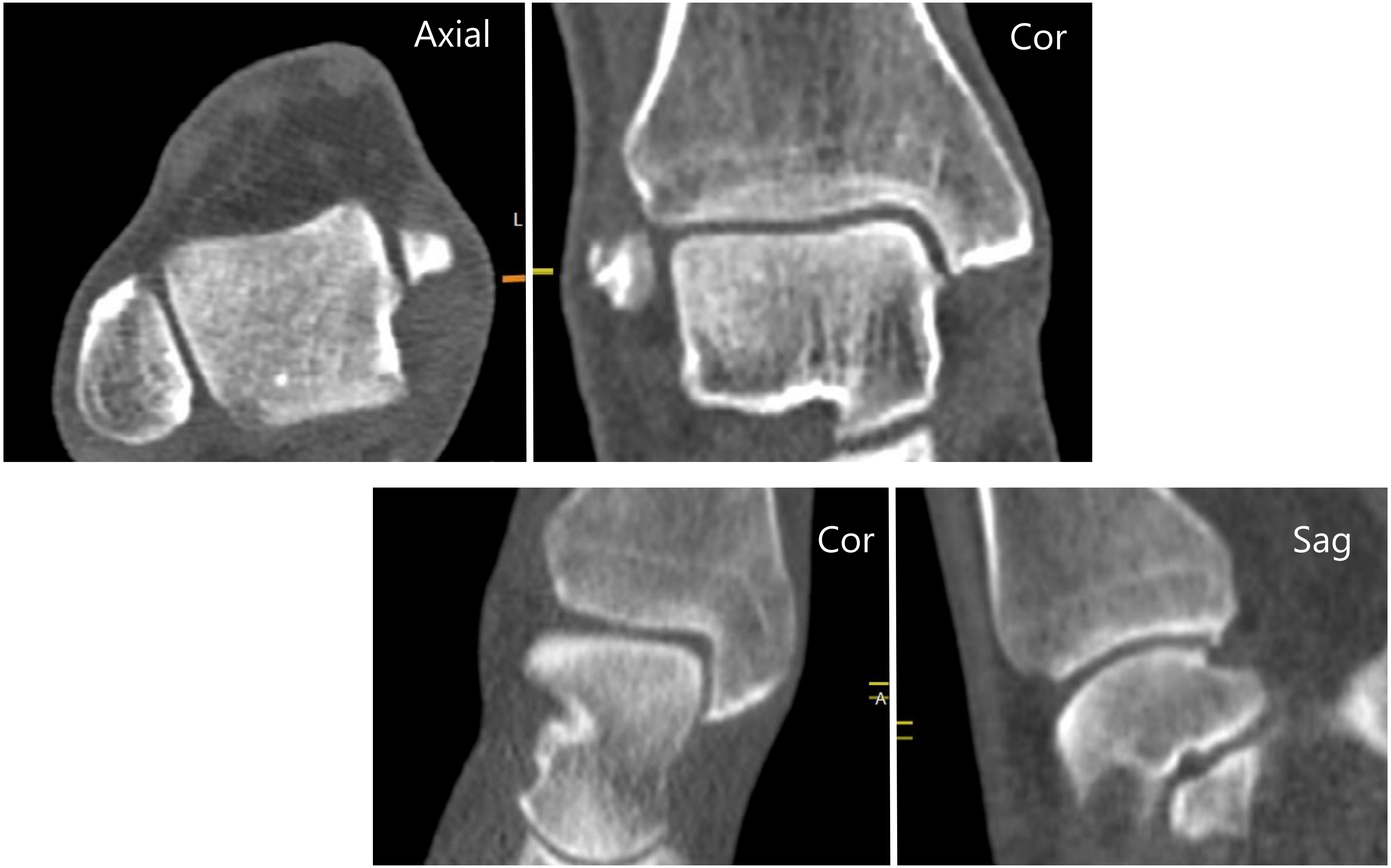

Ankle 2

Ankle	Definition
0	No clinical evidence of OA; joint space integrity fully intact
1	Mild; osteophyte formation/lipping, possible joint space narrowing
**2**	**Moderate; joint space narrowing evident, obvious osteophyte formation and some sclerosis/cystic changes**
3	Severe; near absence of joint space, severe osteophyte/cyst formation, deformity of bone

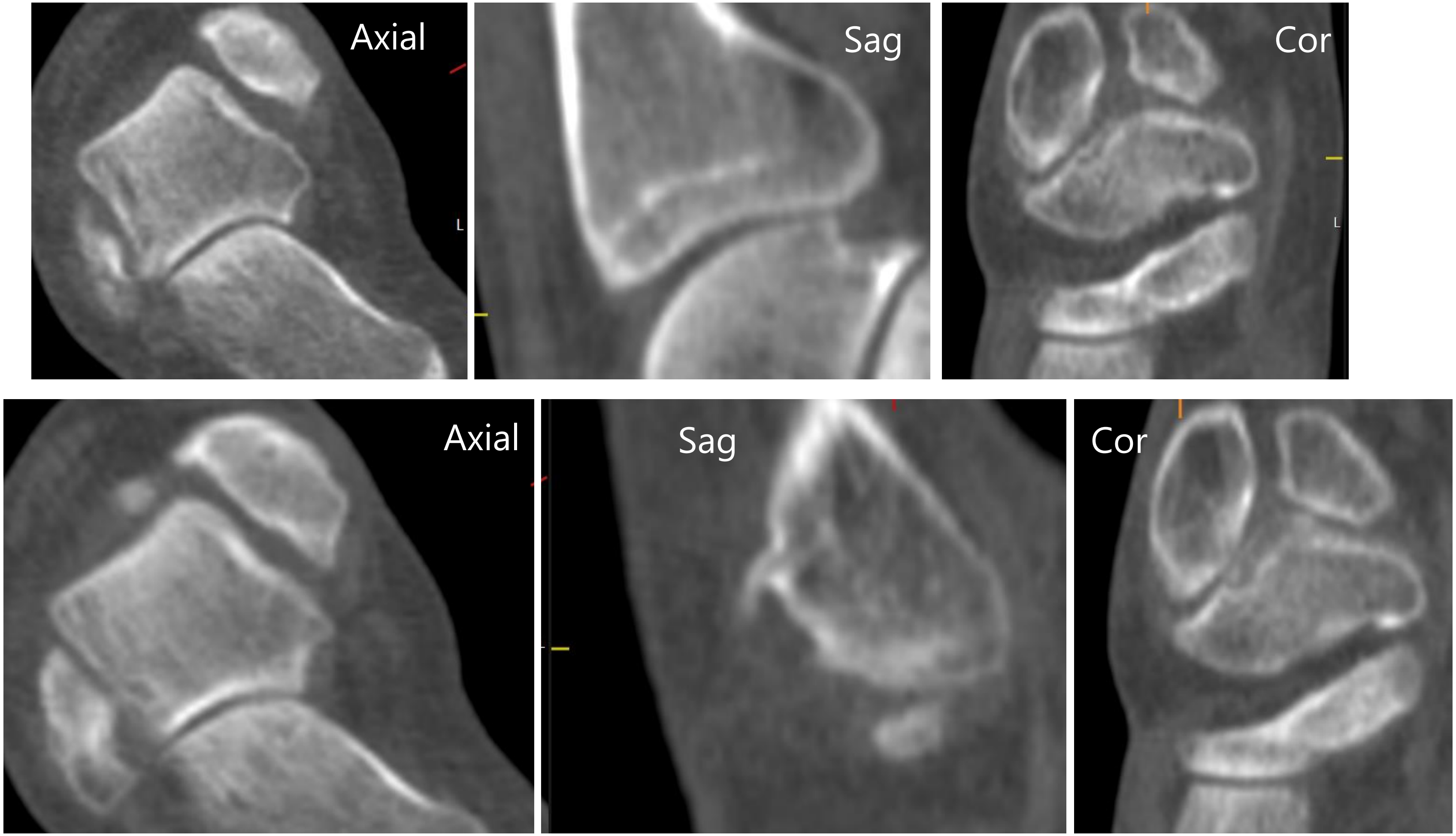

Ankle 3

Ankle	Definition
0	No clinical evidence of OA; joint space integrity fully intact
1	Mild; osteophyte formation/lipping, possible joint space narrowing
2	Moderate; joint space narrowing evident, obvious osteophyte formation and some sclerosis/cystic changes
**3**	**Severe; near absence of joint space, severe osteophyte/cyst formation, deformity of bone**

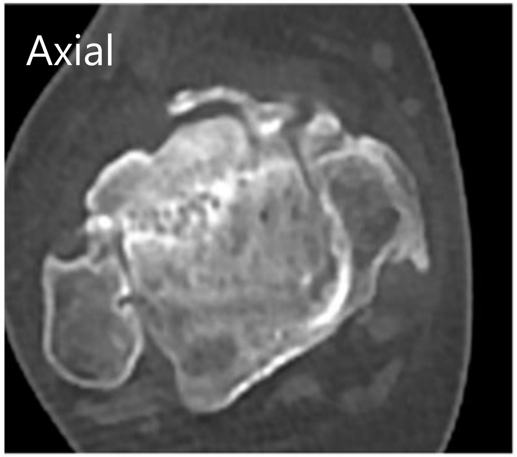

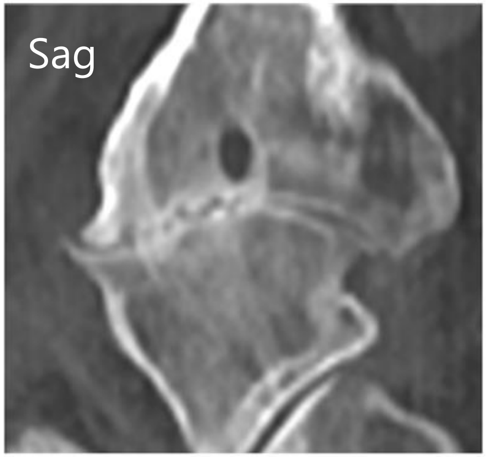

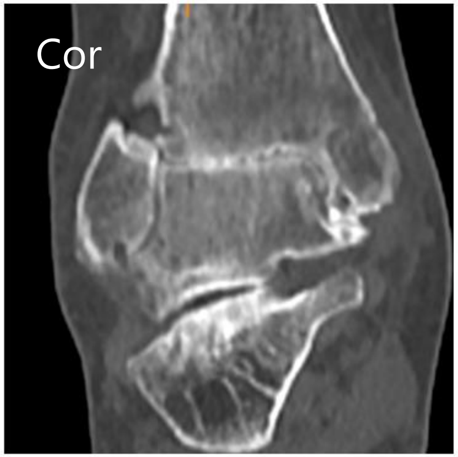

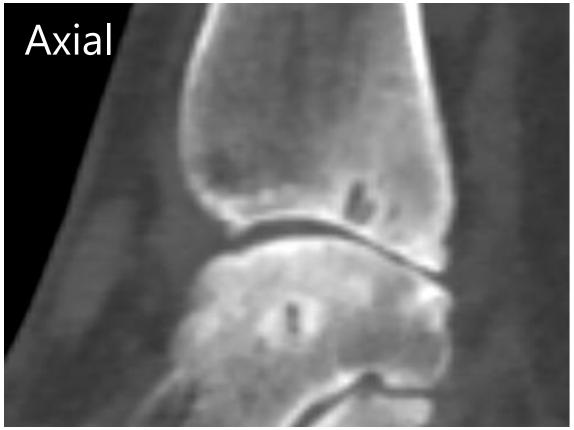

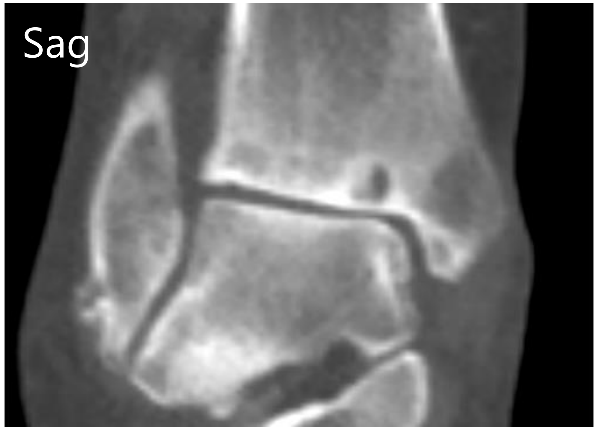

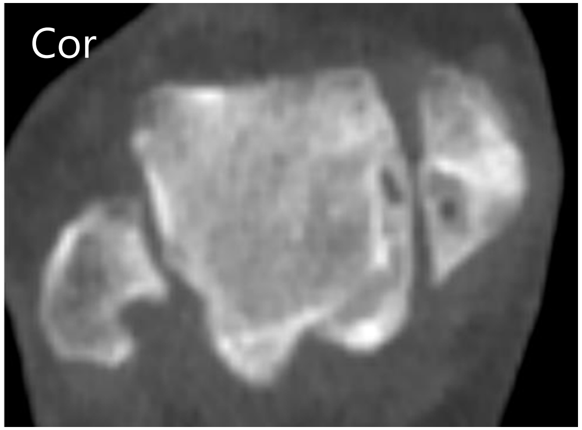

Supplement: Supplementary file 1 [file jpm-11-00005-s001.pdf]
